# Pivotal mental states

**DOI:** 10.1177/0269881120959637

**Published:** 2020-11-11

**Authors:** Ari Brouwer, Robin Lester Carhart-Harris

**Affiliations:** Centre for Psychedelic Research, Imperial College London, London, United Kingdom

**Keywords:** Stress, serotonin, psychedelic, spiritual experience, psychosis

## Abstract

This paper introduces a new construct, the ‘pivotal mental state’, which is defined as a hyper-plastic state aiding rapid and deep learning that can mediate psychological transformation. We believe this new construct bears relevance to a broad range of psychological and psychiatric phenomena. We argue that pivotal mental states serve an important evolutionary function, that is, to aid psychological transformation when actual or perceived environmental pressures demand this. We cite evidence that chronic stress and neurotic traits are primers for a pivotal mental state, whereas acute stress can be a trigger. Inspired by research with serotonin 2A receptor agonist psychedelics, we highlight how activity at this particular receptor can robustly and reliably induce pivotal mental states, but we argue that the capacity for pivotal mental states is an inherent property of the human brain itself. Moreover, we hypothesize that serotonergic psychedelics hijack a system that has evolved to mediate rapid and deep learning when its need is sensed. We cite a breadth of evidences linking stress via a variety of inducers, with an upregulated serotonin 2A receptor system (e.g. upregulated availability of and/or binding to the receptor) and acute stress with 5-HT release, which we argue can activate this primed system to induce a pivotal mental state. The pivotal mental state model is multi-level, linking a specific molecular gateway (increased serotonin 2A receptor signaling) with the inception of a hyper-plastic brain and mind state, enhanced rate of associative learning and the potential mediation of a psychological transformation.

## Introduction

*One way of explaining quantum change experiences is that they represent a kairos, a turning point in the life journey where major change simply must occur because the person in unable or unwilling to continue in his or her present course. It is a point of desperation, a breaking point where ‘something has to give’ – and it does.*

(Miller and C’de Baca, 2001, *Quantum Change*)

Psychological transformation (defined here as rapid, marked and enduring psychological change, where ‘psychological’ refers to perception, cognition and action or behaviour) has been the focus of previous psychological and philosophical texts (Miller and C’de Baca, 2001; Paul, 2014) as well as influential therapeutic programmes (Wilson and Cohen, 2015), but it has received surprisingly little formal scientific investigation and past definitions have been vague. This paper aims to rectify this by proposing a multi-level, biologically informed, context-dependent and process-based approach to the phenomenon. Advancing previous work, we introduce a potentially useful new construct, the pivotal mental state (PiMS). Focusing on psychological transformation as a *process* is a simple but important aspect of our approach that enables us to offer a potential explanation for how transformative experiences (experiences mediating psychological transformation) can manifest into extremely divergent outcomes, such as positively life-changing spiritual breakthrough versus descent into a potentially life-long psychotic illness. More concretely, we propose that however divergent the nature of the transformations themselves, many can be traced to somewhat consistent triggering conditions, with chronic stress being a primer and acute stress a trigger. In what follows, we highlight striking similarities in the conditions of induction, neuropharmacology, neurobiology and psychology of transformative experiences. We demonstrate that these similarities are most compelling when one focuses on the states *preceding* and *mediating* psychological transformations, but become obscured when one selectively attends to the *products* of the transformations themselves.

**Figure 1.**
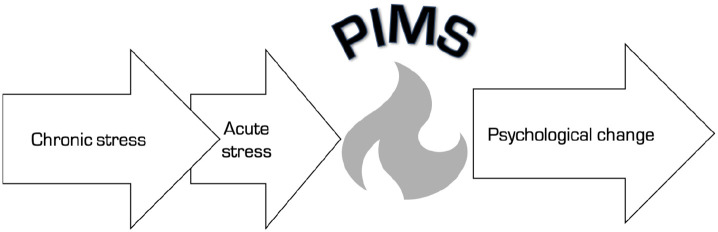
Process-based representation of the pivotal mental state (PiMS). Chronic stress is the primer and acute stress is the trigger of a PiMS, which functions as a mediating state for rapid psychological change. Defining properties of PiMSs: 1. Elevated cortical plasticity. 2. Enhanced rate of associative learning. 3. Elevated capacity to mediate psychological transformation.

In the same way that traumatic experiences can trigger post-traumatic stress disorder (PTSD) or post-traumatic growth (Tedeschi, 1999), we hypothesize that intense periods of psychological crisis can serve to kindle conditions for major, potentially lasting, psychological change, pivotable either towards illness or wellness (Figure 1). This process will later be linked to the phenomenon of bifurcation as described in dynamical systems theory (Kielhöfer, 2011). PiMSs share many parallels with so-called ‘quantum change’ experiences, which have been defined as ‘v*ivid, surprising, benevolent, and enduring personal* [psychological] *transformation*[s]’ (Miller and C’de Baca, 2001). As will become clear, however, we offer a more precise *state* (as opposed to *outcome*) focused, neurobiologically grounded definition of PiMSs that links their hypothesized neuropharmacology and systems-level neurophysiology with their phenomenology. Our broad definition of PiMSs is that they are *transient, intense hyper-plastic mind and brain states, with exceptional potential for mediating psychological transformation*. We sharpen this definition by suggesting three key identifying criteria: (a) *elevated cortical plasticity*, (b) *an enhanced rate of associative learning* and (c) *a unique capacity to mediate psychological transformation*.

The study of the nature and causes of PiMSs and their close association with psychological transformation is a central focus of this review. Our approach is multi level and integrative, addressing the neurobiology, pharmacology and physiology of PiMSs as well as their psychology. In keeping with popular contemporary psychological perspectives (Hayes, 2019; Hayes et al., 2013) as well as the biopsychosocial approach to mental health (Engel, 1977), we are mindful of the essential role played by *context* in shaping the quality and influence of PiMSs. It is our view that the principle of qualifying the longer-term impact of PiMSs by the context(s) or ‘relational frames’ (Hayes, 2019) in which they arise subside and potentially recur is vital for resolving the wellness versus pathology paradox, namely whether positive or negative changes in mental health follow from a PiMS.

**Figure 2.**
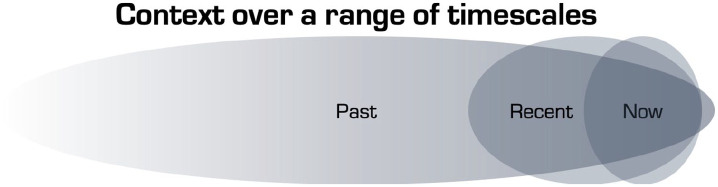
Schematic that acknowledges the breadth of timescales over which contextual factors can influence one’s current state. The boundaries between the three timeframes are arbitrary but one may think of ‘past’ in terms of, for example, an enriched versus adverse childhood or any past trauma but it can also include what is inherited (i.e. genetically encoded). The ‘recent’ past might include such things as a recent relationship breakdown or a bereavement, ongoing cultural or social crisis, or recent success or cause for celebration. What is happening in one’s immediate environment (i.e. ‘now’) might be easier to observe, describe and manipulate but is not necessarily the most potent influencer of one’s present state. This schematic is intended to acknowledge the temporal range of contextual influences that can impinge on the quality and outcome of a pivotal mental state (PiMS).

Unlike past literature on quantum change, our PiMSs model *does not* favour positive outcomes over negative, speaking to an essential context and relational dependency, where context refers to both biological (e.g. polygenic) predisposition as well as the individual’s immediate and remote environmental context. Thus, the term ‘context’ is used here in an extended biopsychosocial and temporal way (Figure 2).

If we are correct that context plays a key role in shaping the outcome of a PiMS, an important implication is that contextual factors need to be treated with special attention and therapeutic care (where possible) if an individual’s psychological wellbeing is to be safeguarded, whenever a PiMS arises or seems imminent (Carhart-Harris et al., 2018c; Hartogsohn, 2016; Hayes, 2019; Johnson et al., 2008; Leary et al., 1963). This said, we recognize that certain emergent PiMSs – such as manic psychotic states – may, however, be particularly difficult to manage via the manipulation of current environmental context alone, particularly during the active PiMS itself. Thus, we do not advocate for environmental contextual management as an exclusive therapeutic strategy and neither do we argue against pharmacological intervention.

The psychopharmacology of PiMSs takes centre stage in this article. We place special focus on the serotonin system and its 2A receptor (5-HT2AR) subtype in particular, which has been shown to be particularly implicated in biology x environment interactions (Chang et al., 2017; Dressler et al., 2016; Fiocco et al., 2007; Jiang et al., 2016; Jokela et al., 2007; Lebe et al., 2013; Mellman et al., 2009; Parade et al., 2017; Salo et al., 2011). Various acute stressors appear to be reliable and robust inducers of serotonin release (Adell et al., 1997; Amat et al., 1998, 2005; Bastani et al., 2017; Beitia et al., 2005; Bland et al., 2003; Cohen et al., 2015; Ferres-Coy et al., 2013; Fuenmayor and Garcia, 1984; Fujino et al., 2002; Gardner et al., 2005; Hale et al., 2011; Higuchi et al., 2019; Ishida et al., 1997; Ishiwata and Greenwood, 2018; Johnson et al., 2005; Keeney et al., 2006; Kelly et al., 2011; Li et al., 2015; Myers and Beleslin, 1971; Nakajima et al., 2009; Neugebauer, 2020; Paul et al., 2011; Rex et al., 2005; Yoshioka et al., 1995) and different types of stressor, such as sleep deprivation (Elmenhorst et al., 2012; Maple et al., 2015; Zhao et al., 2019), hypoxia (Anju et al., 2011), chronic tryptophan depletion (Cahir et al., 2007), inflammation (Zhang et al., 2001), tonic pain (Kupers et al., 2009), repeated forced swim (Takao et al., 1995), repeated shock (Dwivedi et al., 2005), administration of stress hormones (Jitsuiki et al., 2000; Kuroda et al., 1992, 1994), amygdala stimulation (Kalynchuk et al., 2006), time-dependent stress (Harvey et al., 2003), novelty stress (Aloyo and Dave, 2007), maternal separation (Benekareddy et al., 2010, 2011; Godar et al., 2019; Vazquez et al., 2000; although see Ohta et al., 2014), isolation rearing (Preece et al., 2004; Rilke et al., 1998), isolation housing (Günther et al, 2008; Schiller et al., 2003; although see Bibancos et al., 2007; Schiller et al., 2003) and social defeat (Berton et al., 1997; McKittrick et al., 1995; although see Visser et al., 2014) all appear to upregulate 5-HT2AR expression, particularly in the cerebral cortex (Anju et al., 2011; Benekareddy et al., 2010, 2011; Berton et al., 1997; Cahir et al., 2007; Dwivedi et al., 2005; Elmenhorst et al., 2012; Godar et al., 2019; Günther et al, 2008; Harvey et al., 2003; Jitsuiki et al., 2000; Kalynchuk et al., 2006; Kupers et al., 2009; Kuroda et al., 1992, 1994; Maple et al., 2015; McKittrick et al., 1995; Ossowska et al., 2001; Preece et al., 2004; Rilke et al., 1998; Takao et al., 1995; Vazquez et al., 2000; Zhao et al., 2019; see also Table 1), which is well known to be massively expanded in humans.

**Table 1.** The 5-HT2AR mediates responses to various types of stress.

Stress	Type	Finding	Citations
Cognitive stress	Chronic stress Anxiety Depression Neuroticism Panic Trauma Suicide	***Upregulated 5-HT2AR binding or expression***	**Amidfar et al., 2017; Anisman et al., 2008; Bhagwagar et al., 2006; Dwivedi et al., 2005; Fernandes et al., 1997; Frokjaer et al., 2008; Meyer et al., 2003; Ossowska et al., 2001; Pandey et al, 2002; Shelton et al., 2009; Stanley and Mann, 1983; Takao et al., 1995; Turecki et al., 1999**
		*Contradictory or negative findings*	Chaouloff et al., 1994; Mann et al., 2019; Steinberg et al., 2019; Xu et al., 2016
		***5-HT2AR-mediated responses***	**Buchanan et al., 2015; Chang et al., 2017; Chaouloff et al., 1994; Jaggar et al., 2017; Lee et al., 2007; Magalhaes et al., 2010; Mellman et al., 2009; Miller et al., 2016; Xiang et al., 2019**
		*Contradictory or negative findings*	Jaggar et al., 2017; Peričić, 2003; Xu et al., 2016; Yamada et al., 1995
Social stress	Social defeat Social isolation	***Upregulated 5-HT2AR binding or expression***	**Benekareddy et al., 2011; Berton et al., 1997; Godar et al., 2019; Günther et al., 2008; McKittrick et al., 1995; Preece et al., 2004; Rilke et al., 1998; Vazquez et al., 2000**
		*Contradictory or negative findings*	Bibancos et al., 2007; Ohta et al., 2014; Schiller et al., 2003; Visser et al., 2014
		***5-HT2AR-mediated responses***	**Beig et al., 2009; Benekareddy et al., 2010, 2011; Brotto et al., 1998; Clinard et al., 2015; Geyer et al., 1999; Gorzalka et al., 1998; Rillich and Stevenson, 2018; Sakaue et al., 2002; Sinh and Ootsuka, 2019; Wright et al., 1991**
		*Contradictory or negative findings*	None found
Physiological stress	Inflammation Hypoxia Pain Food restriction Sleep deprivation Temperature regulation	***Upregulated 5-HT2AR binding or expression***	**Anju et al., 2011; Cahir et al., 2007; Dwivedi et al., 2005; Elmenhorst et al, 2012; Kupers et al., 2009; Maple et al., 2015; Zhao et al., 2019**
		*Contradictory or negative findings*	Cahir et al., 2007
		***5-HT2AR-mediated responses***	**Abbott et al., 1996; Billac and Nichols, 2019 (review); Buchanan et al., 2015; Courteix et al., 2018 (review); da Silva Soares et al., 2019; Dean et al., 2019; Flanagan et al., 2019a, 2019b; Mazzola-Pomietto et al., 1995; Nau et al., 2013; Nisijima et al., 2001; Pawlyk et al., 2006; Seyrek et al., 2010; Sinh and Ootsuka, 2019; Yu et al., 2008; Zuo et al., 2019**
		*Contradictory or negative findings*	Courteix et al., 2018 (review); Zamfir et al., 1992

Accumulating evidence implicates the 5-HT2AR system in relation to cognitive, social and physiological stressors. In the ‘citations’ column are citations of studies that found increased 5-HT2AR binding, protein or mRNA expression in response to a variety of relevant stressors (in **bold)**. Contradictory or negative findings are listed directly below. In the rows entitled ‘5-HT2AR-mediated responses’ we cite studies that have reported evidence of 5-HT2AR mediated responses to, and sensitization in response to, relevant stressors. It is worth noting this table is not intended to be exhaustive but does cover stressors that have been closely associated with 5-HT2AR signaling and PiMS-related outcomes.

5-HT2AR: serotonin 2A receptor.

Social isolation and defeat also reliably sensitize behavioural responses to 5-HT2AR agonists (Benekareddy et al., 2010; Brotto et al., 1998; Gorzalka et al., 1998; Sakaue et al., 2002; Sood et al., 2018; Wright et al., 1991; see also Table 1). The role of stress and the 5-HT2AR system has been the focus of two recent review papers (Carhart-Harris and Nutt, 2017; Murnane, 2019). Supplementing these findings, direct agonism of the 5-HT2AR via psychedelic drugs can sometimes induce psychological states exhibiting phenomena that mimic those seen in extreme stress states, for example, enhanced associative learning and a significant capacity for mediating psychological transformation (Briere et al., 2015; Hefferon et al., 2009; Joëls et al., 2006), as can occur via traumatic encounters. Psychedelics have also been shown to increase the release of stress hormones (Alper, 1990; Calogero et al., 1989; Dos Santos et al., 2012; Hasler et al., 2004; Owens et al., 1991; Schmid et al., 2015; Strajhar et al., 2016; see Schindler et al., 2018 for review).

We highlight how certain natural inducers of stress such as social isolation, starvation, atypical breathing, sleep deprivation, extreme body temperature and pain can be intentionally manipulated for the purposes of personal and/or spiritual development, presumably by promoting endogenous psychedelic-like signaling and we review several cross-cultural, historical and modern examples of such manipulation (Camporesi, 1988; Farré-i-Barril, 2012; Garrett et al., 2011; Grof and Grof, 2010; Janssen et al., 2016; Macmillan, 2013; Naor and Mayseless, 2017).

In keeping with principles of bidirectional translation (Jia, 2016), research with psychedelic (‘mind-manifesting’) drugs can inspire research into the pharmacology and phenomenology of endogenously occurring PiMSs, which, in turn, helps inspire hypotheses on the function of 5-HT2AR signaling, including how it relates to mental illness and its treatment, such as via psychedelic therapy (Carhart-Harris and Goodwin, 2017), plus other methods of manipulating PiMSs for psychotherapeutic ends (Fosha, 2000; Grof and Grof, 2010; Kuijpers et al., 2007). All classic serotonergic psychedelic drugs have direct agonist properties at the 5-HT2AR (Nichols, 2016), and the key role this particular receptor plays in mediating their signature behavioural effects is supported by: (a) affinity-by-potency relationships in animals and humans (Glennon et al., 1984; Rasmussen et al., 1986; Sadzot et al., 1989); (b) a plethora of antagonist pre-treatment studies (e.g. Preller et al., 2017; Quednow et al., 2012; Vollenweider et al., 1998); and (c) evidence of a 5-HT2AR occupancy by subjective effects relationship (Madsen et al., 2019). A wealth of evidence now exists that, via their action at the 5-HT2AR (Madsen et al., 2019), psychedelics, at relevant dose ranges, reliably trigger conditions conducive to psychological transformation (Carhart-Harris and Goodwin, 2017).

Thus, identifying the 5-HT2AR as a key trigger site for inducing PiMSs, we propose that psychedelics hijack the same neurochemical mechanisms that are engaged during, and likely exist for, situations where a hyper-plastic state and associated psychological change is felt as needed. Developing an understanding of endogenously occurring PiMSs can thus shed light on the evolutionary function of brain 5-HT2AR, as well as the action of psychedelics themselves. We propose that the mechanisms underlying PiMSs have evolved to aid *rapid and deep learning* in situations of perceived or actual existential threat or crisis for the ultimate purpose of catalyzing psychological *change* when (perceived) circumstances demand this. Somewhat consistent ideas have been expressed in the past (Jaynes, 1976), and more recently in two separate reviews (e.g. Carhart-Harris and Nutt, 2017; Murnane, 2019), but are more fully developed here.

We conclude this article by restating the principle that although PiMSs may be associated with major psychological change, the quality of such change is neither consistent nor pre-determined. We propose that the surrounding context and relational frame in which a given PiMS occurs is a vital determinant of how it manifests (Hayes et al., 2013). Thus, we end this paper by discussing how engineering of optimal contextual frames (as far as this is possible), including not just the containing environment for the PiMS itself but also prior intentions and integration work after the event (Carhart-Harris et al., 2018c; Kornfield, 2001) can enable PiMS-related psychotherapy to be delivered most safely and effectively.

We focus on psychedelic therapy as a prototypical PiMS-focused intervention but there are other relevant examples. Indeed, PiMS-focused therapeutic work can be viewed as a more fundamental therapeutic approach than psychedelic therapy, with the latter merely representing one (particularly potent) example (Carhart-Harris, 2018a). We believe that a growing appreciation of PiMSs could inspire a healthy pivot in mental healthcare and research, more firmly towards the biopsychosocial model (Engel, 1977). Psychedelic therapy, and the PiMS-based model more generally, are quintessentially ‘biopsychosocial’ (Deacon, 2013) as they recognize how social, psychological and biological factors interact in bidirectional, synergistic ways to determine health and illness. The PiMS model purports to explain how biopsychosocial synergies can be harnessed for the delivery of improved mental healthcare. Such improvements are needed if the significant burden of mental illness is to be properly addressed (World Health Organization, 2017). Elevating an awareness of PiMSs as key states of mind and brain, with a heightened potential for mediating lasting change, may have implications for the scientific study of a range of PiMSs-related approaches, such as ‘breathwork’ (Grof and Grof, 2010), meditation (Kuijpers et al., 2007) and accelerated psychotherapies (Fosha, 2000), which should serve to develop their shared validity.

Before beginning our detailed review of relevant literature pertaining to the PiMSs construct, it feels necessary to flag some of its complications early on. For example, it is an implication of the ‘outcome agnosticism’ of the PiMS model that these states be capable of fomenting iatrogenic outcomes, that is, a worsening or triggering of psychopathology (Erritzoe et al., 2017; Strassman, 1984), if the surrounding context is negative, for example, as is often the case in the aetiology of psychosis (Varese et al., 2012b) and perhaps even more plainly, PTSD (Kilpatrick et al., 2003). This matter is highly relevant to inappropriate and/or unsupported psychedelic drug use, as well as malpractice in any kind of paediatric, pastoral or healthcare sector where individuals exhibiting elevated brain plasticity are implicated (Carhart-Harris et al., 2018c; Kornfield, 2001; Schlosser et al., 2019). A wealth of evidence from psychedelic drug, child psychology and mental health research can be cited to highlight the importance of contextual factors, such as: (a) preparedness, (b) intentions and expectations, (c) inter-personal trust/therapeutic alliance, (d) community support and (e) other forms of psychological integration, for safeguarding against harm and enhancing positive therapeutic outcomes (Carhart-Harris et al., 2018c; Kessler et al., 2010).

We also wish to recognize the stabilizing influence that certain implicit assumptions can have on one’s mental state, even if they are symptomatic of mental illness. One should therefore be mindful of potential risks entailed by destabilizing such beliefs, either via the direct action of psychedelic drugs or other means; see Letheby (2016) for a relevant discussion paper. Delusional beliefs are a good example of psychologically stabilizing, but plainly pathological, beliefs. However, we also highlight evidence supporting a role for destabilization as mechanism of therapeutic change (Olthof et al., 2019). Themes of psychological flexibility (Zhang et al., 2018) and experiential acceptance (Rochefort et al., 2018; Watts et al., 2017) are also relevant here.

Relatedly, the position that PiMSs are ripe mediators of psychological transformation, whether towards pathology or away from it, could be viewed as unjustly dismissive or neglectful of the important contribution made by factors such as polygenic predisposition (Davis et al., 2016) and early life adversity (Szyf, 2013; Varese et al., 2012b) in shaping mental health. Such apparent neglect is not intentional, and indeed we consider the highlighted factors part of the overall contextual/relational frame shaping the onset and outcome of a PiMS, where context is a phenomenon that stretches over a broad timescale (Figure 2). Although the recent and current context surrounding a PiMS might be easier to manipulate in favour of positive outcomes, more remote contextual factors such as polygenic predisposition or childhood trauma could still be accounted for, for example, a high polygenic risk for psychotic symptoms might contraindicate psychedelic therapy and thus be used to inform screening for such therapy.

**Figure 3.**
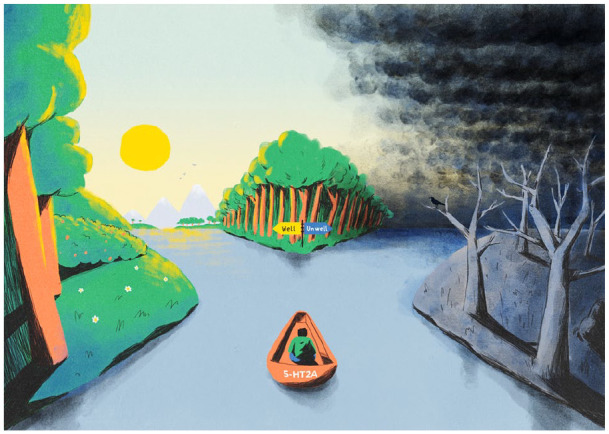
Use of the ‘fork in the river analogy’ to communicate the nature and effect of pivotal mental states (PiMSs). The boat, named ‘5-HT2A’, represents the molecular gateway leading to the occurrence of a PiMS, that is, entry into a hyper-plastic state in which the likelihood of major psychological change is enhanced, and the nature of that change is especially context dependent. The fork in the road reflects a bifurcation and thus can be expected to fall under the principles of bifurcation theory more generally (Kielhöfer, 2011); the details of this are beyond the scope of this article.

A fork in the road or river analogy is often used to reflect a bifurcation process, namely a ‘cross-roads’, where the trajectory of a system can rapidly destabilise and complexify at a point of bifurcation (Kielhöfer, 2011). In the schematic below (Figure 3), the analogy is used in relation to the PiMS. Contextual factors can be viewed as biasing currents in the river influencing particular outcomes, in the same way that cambers or slopes bias outcomes along a solid path. Briefly, bifurcation theory describes the occurrence of sudden changes in the trajectory of a system. Typically, energy introduced into a system causes the destabilization of a previously dominant trajectory or state (e.g. represented by the single path that precedes the fork). The critical destabilization creates new potential states or trajectories that the system can enter or follow. For simplicity, ‘wellness’ and ‘illness’ are presented here as discrete binary states but we recognize that this is an oversimplification and that mixed features are also possible, for example symptoms consistent with post-traumatic *stress* and *growth* can co-exist or ‘flip-flop’ after a traumatic episode, and similarly, mixed euphoric and dysphoric states can occur in manic psychoses. The unifying principle, however, is that *change*, whatever its nature, is more likely after such pivotal events.

## Pivotal mental states and their divergent outcomes

Throughout this paper we argue that PiMS can mediate divergent outcomes that strongly depend on the surrounding context in which they arise. Here we apply this principle to a classic controversy in psychology and psychiatry: namely, the relationship between spiritual experiences and psychosis. It has long been noted that certain spiritual and early and acute psychotic experiences exhibit similar features such as anomalous self-experience, magical thinking and perceptual aberrations (Baldacchino, 2016; Buckley, 1981; Cangas et al., 2008; Crespi et al., 2019; Grof and Grof, 1989; Hunt, 2000, 2007; Jackson, 1997; Jaynes, 1976; Luhrmann, 2017; Lukoff, 1985, 2007, 2018; Murray et al., 2012; Parnas and Henriksen, 2016; Perry, 1977; Polimeni, 2018; Powers and Corlett, 2018; Ross and McKay, 2018; Willard and Norenzayan, 2017). All three phenomena are reliably induced by 5-HT2AR agonist psychedelics (Carhart-Harris, 2007; Carhart-Harris and Friston, 2010; Kraehenmann et al., 2017; Letheby, 2016; Millière, 2017; Nour et al., 2016), thus implying their relationship to a more fundamental state – the 5-HT2ARR-mediated PiMS.

**Figure 4.**
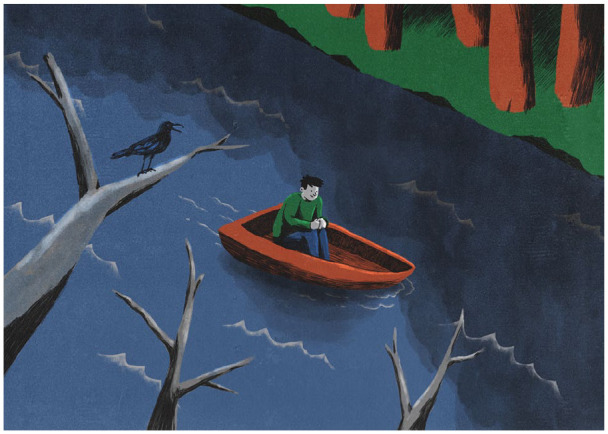
How an outcome-biased or outcome-focused perspective can obscure the role of a common root state preceding a bifurcation that led to the outcome. Although a particularly stark outcome may manifest (e.g. the development of a psychotic episode or disorder), the implication is that this may not have been an inevitability and measures can be taken to either shepherd the pivotal mental state (PiMS) in a particular direction or even seek to avoid it altogether, for example, in cases where there are polygenic vulnerabilities.

According to the model presented here, the framing of psychosis as pathological and spiritual experience as psychologically beneficial, obscures their common relationship to the PiMS (Jackson, 1997). Indeed, a negative or positive *outcome* is the paramount criterion for distinguishing between mystical and psychotic experience, respectively (DeHoff, 2015; James, 2003 [1902]). Moreover, scales used to measure trait schizotypy and spirituality also contain items relating to social and emotional adjustment, behaviour and appearance (Davidson et al., 2016; Garssen et al., 2016; Raine, 1991; Raine et al., 1994), which capture divergent responses to ego-disturbance, perceptual aberration and magical thinking (Crespi et al., 2019). It is our view that certain psychoses and spiritual or religious conversions share a common heritage in the PiMS. We also argue that this connection is often overlooked because of a tendency to selectively focus on outcomes rather than the processes that lead up to them (Figure 4). The mediational states themselves are often relatively brief and intense, but they can also be more protracted. We describe them as ‘pivotal’ based on the principle that a consistent root state can mediate strongly divergent outcomes, such as spiritual or religious epiphany or conversion versus the acquisition of a psychotic delusion. The Oxford definition of a pivot (as a noun) is a central point or person from which a mechanism turns or oscillates (*Oxford English Dictionary*, pivot, def. 1., n.d.) and the adjective ‘pivotal’ is defined as ‘[a thing] of crucial importance in relation to the development or success of something else’. We therefore use the adjective here in reference to the principal properties and function of PiMSs, that is, a heightened ability to mediate major psychological change. We propose that a focus on the root state preceding a given outcome should reveal the presence of the defining properties of PiMSs.

In this paper, we use a recent definition of stress as ‘*the body’s multi-system response to any challenge that overwhelms, or is judged likely to overwhelm, selective homeostatic response mechanisms’* (Day, 2005). The cited paper is a useful reference for reviewing alternative definitions. Discussing different definitions of stress is beyond the remit of the present paper but it is relevant to note that most definitions appear to agree that stress is a multi-level response to an apparent threat to an organism’s present state. This consistent definition of stress is useful, as it acknowledges how stress can engage adaptive mechanisms – such as heightened plasticity – that is, the quality of being more shapeable (*Oxford English Dictionary*, plasticity entry, n.d.). Appreciating the link between stress and adaptability can help us understand the etiological and evolutionary function of PiMSs. As we show below, stress is a particularly robust and reliable primer, trigger and potentiator of PiMSs (van der Steen et al., 2017).

**Figure 5.**
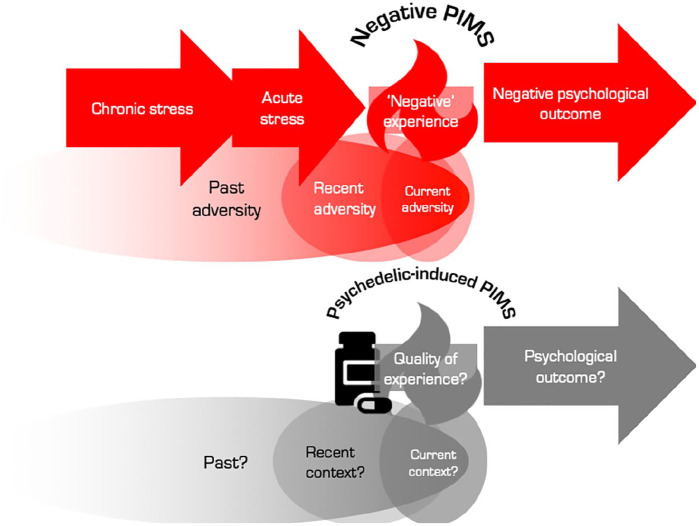
The upper process is perhaps the most typical scenario for a naturally occurring pivotal mental state (PiMS) but also the worst possible scenario as far as long-term mental health is concerned. It is the scenario that, we believe, most often accompanies the development of a psychosis. The lower process can be described as ‘outcome agnostic’ in the sense that the quality of the PiMS itself and its subsequent impact on mental health is contingent on the surrounding context and relational frame into which is arises. If the recent, current and post-PiMS context can be shaped favourably, then one can expect the longer-term impact of the PiMS to be favourable also. However, more remote contextual factors (e.g. polygenic make-up or a history of trauma) are, understandably, harder to manipulate and may, on occasion, merely signal that a PiMS could be especially risky, for example, in terms of its outcomes. This said, it is entirely plausible that naturally occurring PiMS can yield favourable psychological outcomes.

Stress is typically construed of and felt as an aversive phenomenon and recognizing this can help us understand why naturally arising PiMSs often lead to negative outcomes (Figure 5). For example, if a PiMS is not expected and the experience is protracted, then the net effect of this is likely to be distressing (Rekhi et al., 2019; van der Steen et al., 2017). Adverse conditions are, almost by definition, stressful and classic examples such as unintentional social isolation, urban-environment-related stress, poor socioeconomic status and childhood adversity are likely to mediate and/or potentiate PiMS, and have all been significantly linked with psychotic disorders (Selten and Cantor-Graae, 2005, 2007; Selten et al., 2013, 2016). In contrast, spiritual experiences appear to be more likely to arise in positive environmental contexts, such as in nature (Anderson et al., 2018).

The bidirectional and often mutually reinforcing relationship between belief and affect is reflected in emotionally antithetical experiences of self-dissolution in spiritual versus psychotic experiences. In severe and enduring psychosis, self-fragmentation (Millière, 2017; Parnas and Henriksen, 2016) is often felt as invasive and torturous, whereas sensations of ‘mystical union’ or ‘inter-connectedness’ (Carhart-Harris et al., 2018b) appear to lie at the heart of positive experiences of ego-dissolution in naturally occurring (Stace, 1960) and psychedelic-induced spiritual experiences (Millière, 2017; Nour et al., 2016; Roseman et al., 2018). See Parnas and Henrikson (2016) for another detailed comparison of mystical and psychotic experience. We are mindful, however, that manic episodes can also have a euphoric quality and thus, euphoric states are not therapeutic themselves (Masters, 2010).

Increased emotional tone is a common feature of PiMSs that likely plays an important role in modulating their immediate nature and longer-term impact. Recent work with psychedelics has revealed that ‘emotional breakthrough’ is an important and distinct mediator of long-term positive outcomes after psychedelic therapy (Roseman et al., 2019). Relatedly, feelings of ‘awe’ in nature have been found to mediate improvements in wellbeing via nature exposure (Anderson et al., 2018). The active suppression of emotion is associated with the nature and severity of symptomatology in psychosis and PTSD (Laloyaux et al., 2016; Roemer et al., 2001; Tull et al., 2018) and the duration of negative affect (but not its intensity) has been found to predict negative long-term outcomes after psychedelic drug use (Carbonaro et al., 2016).

A logical explanation for the impact of intense emotion on long-term psychological outcomes from PiMSs is that the felt emotion modulates the nature of associations made during the pivotal state and intensifies their influence, allowing for beliefs and perspectives to be affected. In predictive processing terms, emotion can be thought of as prediction error modulating the ‘precision’ (inverse variance) of ‘posteriors’ or ‘priors’ (probability distributions in the Bayesian sense, i.e. the expected likelihood of a set of possibilities). Expressed in a way to serve readers’ intuition: at a high hierarchical level, the precision of a prior relates to one’s confidence in a given perspective or belief. If the affective tone of a PiMS is positive and intense, this may drive a ‘de-weighting’ or relaxation of a negatively held belief. For example, a negative (cognitive) bias characterizing a depressive disorder (Disner et al., 2011) would be felt less confidently if it were relaxed. However, if the affective tone accompanying a PiMS is intense, negative and unresolved, this may contribute to an instilling and/or reinforcement of negative beliefs – it would add precision to the (negative) belief (Roseman et al., 2019). See Carhart-Harris (2019) for a discussion of related themes.

Increased emotional tone coupled with hyper-plasticity and enhanced associative learning is a potent mix for moderating or reinforcing deeply held beliefs and perspectives. Symbolic associations may become increasingly oriented to one’s underlying emotional state, encouraging the formation of ‘affect-laden’ worldviews. Indeed, if emotion is intensified during a PiMS but its valence is unpredictable, it may explain how a consistent root state can mediate extremely divergent outcomes. The intensity, duration, psychological preparedness and degree of psychological resolution may all contribute to the nature and influence of emotion on the quality and impact of a PiMS (Haijen et al., 2018). Negative affect predominates in schizophrenic psychosis and is associated with delusions of persecution (Paolini et al., 2016) and general cognitive disorganization (Carrigan and Barkus, 2017). Conversely, positive affect predominates in western conceptions of spiritual experience (DeHoff, 2015; Hardy, 1979) as well as psychedelic drug experiences (Liechti et al., 2017), all of which usually feature some degree of positive context.

Manic states could be seen as a challenge to this rule, however. Relatedly, we recognise that persecutory and grandiose thinking and associated negative and positive mood states are not mutually exclusive and can exist in parallel and/or interchangeably, as exemplified by apocalyptic and religious delusions (Iyassu et al., 2014; Wessinger, 2000) and manic psychoses (Picardi et al., 2018). Perceptions of interconnectedness and self-transcendence in manic states bear resemblance to the phenomenological qualities of spiritual states, including those induced by psychedelics (e.g. see Carhart-Harris et al., 2013). Manic states are often triggered by stressful events and culminate in chronic psychotic disorders. Comorbidity of bipolar, schizophrenia and schizoaffective disorders reflects a shared polygenic vulnerability to psychotic symptoms (Cardno and Owen, 2014; Craddock et al., 2009; Laursen et al., 2009) and possibly, by extension, PiMSs. Context is rarely, if ever, either entirely ‘positive’ or ‘negative’ and the same is true for how it is received, that is, context is inherently relational and subjective. Moreover, we recognize the paradox that highly positively valanced mood states, for example euphoric states in manic psychoses or a psychedelic drug experience, do not naturally imply positive long-term outcomes for mental health. The phenomenon of ‘spiritual bypassing’ is relevant in this regard (Masters, 2010). For example, the zealous promotion of self/ego transcendence in the absence of subsequent psychological integration could be regarded as a (subtly) negative, or at least ‘imperfect’, context (Kornfield, 2001).

Psychotic episodes and spiritual experiences are both commonly preceded by a dissatisfaction with reality and one’s place within it (Hardy, 1979; James, 2003 [1902]; Miller and C’de Baca, 2001). In the prodrome to psychosis (i.e. a pre-psychotic period that precedes a conspicuous psychotic episode) an individual’s presentation may often resemble that of a depressive phenotype (Corcoran et al., 2011; DeVylder et al., 2014; Rietdijk et al., 2013). Indeed, an individual entering a PiMS (whether interpreted as psychotic, spiritual, both or neither) may perceive their world as unreal, thus spurring belief in a ‘meaningful’ but concealed other reality (James, 2003 [1902]: 149–155). One should be mindful how transition into a psychotic episode or spiritual experience can be construed as a ‘manic defence’ (Winnicott, 1935), that is, an escapist ‘flight from reality’. See again Masters (2010) on the topic of ‘spiritual bypassing’.

In longer prodromal states, a loss of interest in key activities and pursuits (e.g. education and vocation) and pleasures (e.g. food, sex, social interactions) can coexist with an emerging interest in supernatural paranormal, religious and ritual domains (Møller and Husby, 2000, 2003). Thoughts may begin to feel unrelated to the self and emotionally distant (Parnas and Handest, 2003). Individuals may find themselves examining their thoughts and behaviours from a third-person perspective (Nelson et al., 2016), as aware yet dissociated subjects (Parnas and Handest, 2003). A dissociation of subjective awareness from one’s body and/or thoughts evokes common religious/spiritual notions of the incorporeal soul, ‘true-self’ (Ātman) and ‘no-self’ (Anātman) (Parnas and Henriksen, 2016) as well as the related notions of ‘non-dual awareness’ (Josipovic, 2014) and the ‘unitive experience’ (Stace, 1960).

Many religious traditions place value on understanding the nature of the self, its relationship to the world and its transformation. One potentially relevant theme here is ‘salvation’, which, outside of theological contexts, has been defined as a ‘preservation from destruction or failure’ and ‘deliverance from danger or difficulty’ (*Merriam-Webster*, salvation entry, def. 3., n.d.). Thus, the ‘salvific’ and/or adaptive *function* of extraordinary personal experiences has historically been framed in a religious way and religion may function to ease human suffering via other plausible mechanisms. For example, rituals may mitigate anxiety and facilitate healing (Csordas and Lewton et al., 1998; Lang et al., 2015) and absolutist religious beliefs may protect against a basal existential uncertainty. Thus, religion and non-denominational spirituality (whether secular or otherwise) offer potentially useful frameworks/belief systems from which to positively frame experiences that in other contexts might be readily construed as pathological.

## Serotonin, stress and the 5-HT2AR

### Serotonin, coping and adaptation

Serotonin is an endogenous monoamine found throughout the body, particularly in the gastrointestinal system (Gershon, 2013), lungs (Castro et al., 2017) and, to a lesser extent, the central nervous system. Despite its more modest prevalence in the brain, serotonin neurotransmission is known to play an important modulatory role in several key aspects of mind and behaviour, including brain development (Azmitia, 2001), mood (Garcia-Garcia et al., 2017), cognition (Meneses, 1999) and sleep (Jouvet, 1999). Serotonin is a particularly complex neuromodulator, with a broad range of receptor subtypes (i.e. at least 14 different subtypes have been identified to date (Hoyer, 2019), some of which have opposing functions on activation (Andrade, 2011; Araneda and Andrade, 1991)). Previous attempts at a unifying model of the function of brain serotonin have tended to focus on its role in moderating anxiety states (Charney et al., 1987; Deakin and Graeff, 1991; Piszczek et al., 2015) as well as impulsivity/impatience and aggression (Brown and Linnoila, 1990; Fairbanks et al., 2001; Fonseca et al., 2015; McDannald, 2015; Miyazaki et al., 2012, 2014, 2018; Ranade et al., 2014). The most reliable inducers of 5-HT release appear to be stress (Fujino et al., 2002), pain (Harvey et al., 1975) and uncertainty (Miyazaki et al., 2018), see Table 1. Thus, it has been proposed that serotonin’s ‘serenic’ effects (Olivier and Mos, 1990), particularly via non-5-HT2AR (i.e. most notably postsynaptic 5-HT1A receptors in stress circuitry), may be a perceived as an adaptive response to adversity, for example, aiding a type of resilience one might call ‘fortitude’, ‘passive coping’ or an enhanced ability to endure adversity and thus, ‘get by’ (Carhart-Harris and Nutt, 2017).

However, meeting stress with an intention to merely endure may not be an optimal long-term strategy. For example, efforts to suppress and thereby avoid, stress may not be conducive to the revision of (potentially problematic) internal models, such as those linked to cognitive biases in depression, for example. Thus, it seems reasonable to ask: does there not exist an alternative adaptive mechanism, sufficiently different to the stress avoidance/mitigation strategy just described (Carhart-Harris and Nutt, 2017; Wallace, 1956), perhaps one that becomes triggered when adverse conditions surpass a critical threshold of severity and/or chronicity (Dwivedi et al., 2005) such that mere endurance is not enough?

It has been proposed before that a principal function of the serotonin 2A receptor subtype is to induce a state of cortical hyper-plasticity conducive to major adaptive change (Carhart-Harris and Nutt, 2017). The present paper extends this previous work to highlight how chronic stress *primes* the serotonin (2A receptor) system for the elicitation of a PiMS: a hyper-plastic state in which prior assumptions are relaxed, enabling an enhanced sensitivity to potential new information, consistent with rapid and deep learning. In psychosis, this process may result in the maladaptive formation of delusional beliefs that nevertheless help make sense of a frightening world. In spiritual experiences, individuals may report sudden moments of clarity and insight (e.g. epiphanies) servicing positive self-development and renewed perspective (C’De Baca and Wilbourne, 2004; Miller and C’de Baca, 2001). Highly consistent themes can be found in reports of post-traumatic *growth* after recovery from psychosis and other severe conditions (Slade et al., 2019), near-death experiences (Geiger, 2009; Khanna and Greyson, 2015) and in cases of clinical breakthrough with psychedelic therapy (Roseman et al., 2019; Watts et al., 2017). See also Miller and C’de Baca (2001).

Addressing the following questions can help us develop our hypotheses on the role of stress and the 5-HT2AR in PiMSs: 1. Are certain stressors linked with the occurrence of states meeting the definition of a PiMS (e.g. incipient psychosis or spiritual experience)? 2. What types of stress and stressors appear to upregulate the 5-HT2AR system most robustly and reliably? 3. Does increased 5-HT2AR activity facilitate psychotic or spiritual states or traits? 4. Are 5-HT2AR-induced PiMSs associated with major psychological change or transformation? These questions will be addressed in the following sections, starting with an examination of various stressors that facilitate self-transformation and upregulate 5-HT2AR signaling.

### Cognitive stress and the 5-HT2AR

A perceived lack or loss of control is a well-known cause of stress (Steptoe and Poole, 2016) and a potential transdiagnostic factor in a variety of mental illnesses (McEvoy and Mahoney, 2011). Perceived uncontrollability of thoughts correlates with intensification of pathology in obsessive compulsive disorder (OCD) and schizophrenia (García-Montes et al., 2006). In general, negative thoughts are more likely to be misattributed to sources other than the self, reflecting less felt ownership (Swiney and Sousa, 2013). Abnormal meta-cognition (‘thinking about thinking’) is a characteristic of psychosis (Sellers et al., 2017) and schizotypal individuals are also significantly more likely to show abnormal metacognition (Chan et al., 2015). The schizotypal mind may relieve (or reward) itself by hypothetically solving matters of uncertainty via escape into fantasy or delusion, akin to the relief served by compulsive rituals in OCD. A similar function may be served by ritual in religion (Lang et al., 2015). Indeed, the over-weighting of priors (excessive ‘precision’, or inverse variance, synonymous with excessive confidence), particularly in response to perceived uncertainty and loss of control, may be a transdiagnostic feature of psychological suffering (Boswell et al., 2013; Carhart-Harris, 2019; McEvoy and Mahoney, 2011), namely the proposed (defensive) function of many symptoms of psychopathology may be to mitigate uncertainty through the over-weighting of beliefs and/or excessive reinforcement of certain specific behaviours (Boswell et al., 2013; Carhart-Harris, 2019; Carhart-Harris and Friston, 2019).

Neuroticism and depression, indicative of chronic cognitive stress, regularly precede and coexist with psychotic disorders (Corcoran et al., 2011; DeVylder et al., 2014; Lönnqvist et al., 2009; Rietdijk et al., 2013; Van Os and Jones, 2001) and depression and despair also often precede religious experiences (Hardy, 1979). Most studies suggest that chronic stress upregulates 5-HT2AR binding and expression (Dwivedi et al., 2005; Fernandes et al., 1997; Ossowska et al., 2001; Takao et al., 1995; although see Xu et al., 2016) and the 5-HT2AR is implicated in physiological and behavioural responses to chronic stress in humans (Chang et al., 2017; Fiocco et al., 2007; Parade et al., 2017) and animal models (Jaggar et al., 2017; Xu et al., 2016; although see Jaggar et al., 2017). Various studies show upregulated 5-HT2AR transcript and protein expression in neuroticism (Frokjaer et al., 2008) and depression (Amidfar et al., 2017; Bhagwagar et al., 2006; Meyer et al., 2003; Shelton et al., 2009; although see Steinberg et al., 2019), particularly in relationship to dysfunctional attitudes (Baeken et al., 2014; Meyer et al., 2003) and suicide (Anisman et al., 2008; Pandey et al., 2002; Stanley and Mann, 1983; Turecki et al., 1999; although see Mann et al., 2019; Steinberg et al., 2019).

Given the close association between depression and PiMS-related outcomes (i.e. spiritual and psychotic experiences), it is natural to surmise that cognitive-stress-induced upregulation of 5-HT2AR expression may be an important biology x environment interaction through which both spiritual and psychotic experiences manifest via a subsequent increase in 5-HT2AR activation.

Consistent with the PiMS model, baseline neuroticism is associated with elevated cortical 5-HT2AR expression (Frokjaer et al., 2008), predicts thought disturbance, blackout and challenging experiences under psychedelics (Barrett et al., 2016, 2017; Lienert and Netter, 1996; although see Studerus et al., 2012) and yet may be reduced after psychedelic therapy (Erritzoe et al., 2018), presumably because of positive contextual manipulation during the acute hyper-plastic state, as well as afterwards. In relation to naturally occurring PiMSs, both uncontrollable stress (Amat et al., 2005; Bland et al., 2003) and punishment (Cohen et al., 2015; Faulkner and Deakin, 2014) are associated with 5-HT release (Amat et al., 2005; Bland et al., 2003; Cohen et al., 2015; Faulkner and Deakin, 2014).

Psychological trauma predisposes certain individuals towards dissociation, hallucination and other psychotic-like features (Kilcommons and Morrison, 2005; McCarthy-Jones and Longden, 2015; Varese et al., 2012a). There exists an especially high comorbidity between psychotic and PTSD symptoms (Grubaugh et al., 2011; Ng et al., 2016). The 5-HT2AR may mediate altered mind and brain functioning in relation to traumatic events. Consistently, 5-HT2AR variants have been associated with PTSD (Lee et al., 2007; Mellman et al., 2009), symptom severity in PTSD, and the degree of default mode network connectivity amongst people with PTSD (Miller et al., 2016), a network implicated in the action of psychedelics (Carhart-Harris et al., 2012a) that is rich in 5-HT2AR (Beliveau et al., 2017). Animal models implicate the 5-HT2AR in anxiety (Chaouloff et al., 1994; Magalhaes et al., 2010) and anxiety following exposure to trauma (Xiang et al., 2019). Indeed, traumatic experiences are likely to meet all three of the key criteria for a PiMS.

### Social stress and the 5-HT2AR

Social stress and other relevant factors such as urban stress, ethnic minority status (Kirkbride et al., 2012; Veling et al., 2008), migration, childhood trauma, poor cognitive aptitude and drug abuse all contribute to a sense of social defeat and have been linked with schizophrenia (Selten and Cantor-Graae, 2005, 2007; Selten et al., 2013, 2016; Zammit et al., 2010). Social defeat is a phenomenon relative to one’s immediate social surroundings and expectations for the future (Jones et al., 1993; Reininghaus et al., 2008), that is, one’s specific relational frame (Hayes et al., 2013). Social stress has also been linked with higher rates of religious engagement (Aydin et al., 2010; Friedman and Saroglou, 2010; Ghorpade et al., 2006). Loss of close social connections and social isolation have been linked with cognitive-perceptual abnormalities such as a hallucinated sensed presence (Geiger, 2009; Steffen and Coyle, 2010), and loneliness has also been linked with facets of magical thinking such as anthropomorphization (Bartz et al., 2016; Epley et al., 2008; Eyssel and Reich, 2013), which is a feature of religious belief (Guthrie and Guthrie, 1995). Perceived social disconnection, social isolation and social withdrawal regularly precede psychotic and spiritual experiences (Mishlove and Chapman, 1985; Seeman, 2017) and solitary confinement, conceived initially as a method of spiritual rehabilitation, often leads to perceptual distortions, hallucinations, cognitive deficits and paranoia (Haney, 2018). It is also worth highlighting that the classic 5-HT2AR agonist psychedelics quite reliably induce experiences of vivid sensed presence (e.g. Timmermann et al., 2019) as well as magical thinking more broadly (Carhart-Harris et al., 2014a; Kraehenmann et al., 2017).

In preclinical modelling studies, 5-HT2AR antagonism impairs acquisition of conditioned defeat (Clinard et al., 2015; Rillich and Stevenson, 2018) and suppresses hyperthermic response to social defeat (Beig et al., 2009; Sinh and Ootsuka, 2019), whereas agonist administration into the basolateral amygdala increases acquisition of conditioned defeat (Clinard et al., 2015). Acute social defeat increases 5-HT levels (Beitia et al., 2005; Gardner et al., 2005; Higuchi et al., 2019; Keeney et al., 2006; Paul et al., 2011), and region-specific increases in 5-HT2AR expression have been found in chronically subordinate animals (Berton et al., 1997; McKittrick et al., 1995). Acute social defeat has not been shown to upregulate 5-HT2AR expression (Visser et al., 2014) or produce phenotypes indicative of 5-HT2AR sensitization, such as that seen after recurring defeat (Hayashida et al., 2010). These observations are consistent with the present model, which acknowledges that the positive relationship between various stressors and 5-HT2AR upregulation is dependent on the chronicity and severity of that stress.

Maternal separation stress in rodents potentiates the effects of 5-HT2AR agonists (Benekareddy et al., 2010; Sood et al., 2018), and 5-HT2AR antagonists reduce maternal separation-induced anxiety (Benekareddy et al., 2011), aggression and bradycardia (Godar et al., 2019). Preliminary evidence also suggests that maternal separation upregulates 5-HT2ARs (Godar et al., 2019) and 5-HT2AR mRNA expression (Benekareddy et al., 2011; Vazquez et al., 2000; although see Ohta et al., 2014). Isolation rearing likewise seems to upregulate 5-HT2AR expression (Preece et al., 2004; Rilke et al., 1998) and potentiate the effects of 5-HT2AR agonists (Wright et al., 1991). Pre-pulse inhibition deficits displayed by isolation-reared animals are reduced by 5-HT2AR antagonism (Geyer et al., 1999)

The effects of isolation housing on 5-HT2AR expression is inconclusive and time variable, e.g., one study found that 5-HT2AR expression is decreased at 4 weeks (Schiller et al., 2003) whereas others found increases at 6 and 12 weeks (Günther et al., 2008; Schiller et al., 2003), while another found decreased 5-HT2AR mRNA expression at 6 weeks (Bibancos et al., 2007). More evidence is needed to clarify these time-dependent relationships and how they relate to 5-HT2AR mRNA versus protein expression. The effects of isolation on 5-HT2AR sensitization appears more straightforward, with isolation housing in mature animals potentiating 5-HT2AR agonist-induced wet dog shakes (Brotto et al., 1998) and head twitch (Sakaue et al., 2002).

### Physiological stress and the 5-HT2AR

Chronic inflammation, excitotoxicity, hypoxia, metabolic dysfunction, starvation, sleep deprivation and pain are all physiological processes linked to stress. Brain inflammation and excitotoxicity are putative risk factors for psychosis and likely contribute to neurodevelopmental abnormalities (e.g. reduced grey matter volume) in schizophrenia (Plitman et al., 2016; Watkins and Andrews, 2016; Zhang et al., 2016b). Various studies show that 5-HT2AR activation has neuroprotective and anti-inflammatory effects (Billac and Nichols, 2019; Fanibunda et al., 2019; Flanagan et al., 2019a, 2019b; Flanagan and Nichols, 2018; Nau et al., 2013; Yu et al., 2008) and inflammation is a common feature of stress-related disorders (Liu et al., 2017) including psychosis (Fraguas et al., 2019). The general neuroprotective and anti-inflammatory functions of the 5-HT2AR may explain why this receptor is upregulated by and mediates responses to so many stressors (Figure 3) and lends additional support to its candidacy as a therapeutic target (Flanagan and Nichols, 2018).

Hypoxia (oxygen deficiency) may be particularly relevant, as respiratory complications are associated with increased rates of psychosis (Kalucy et al., 2013; Partti et al., 2015). Hypoxia is also implicated in near-death experiences (NDEs) (Klemenc-Ketis et al., 2010; Van Lommel et al., 2001), ‘runners’ high’, high-altitude-induced psychosis (Hüfner et al., 2018), panic attacks (Klein, 1993; Roth et al., 2002) and spiritual practices and experiences (Nivethitha et al., 2017). Hypercapnia (increased CO_2_ in the bloodstream) may mediate the psychedelic-like effects of naturally occurring hypoxic conditions (Klemenc-Ketis et al., 2010). Indeed, there is a history of CO_2_ ‘carbogen’ therapy (Meduna, 1950) that overlaps in some regards with psychedelic therapy, such as with an emphasis on the induction of cathartic experiences.

Hypoxia upregulates cortical 5-HT2ARs (Anju et al., 2011) and the 5-HT2AR agonist psychedelic N,N-Dimethyltryptamine (DMT) exerts neuroprotective effects in cells exposed to hypoxic conditions, leading scholars to suggest endogenous DMT may be released as an adaptive response to physiological stress (da Silva Soares et al., 2019; Szabo et al., 2016). A recent study observed increased DMT levels in rat visual cortex following cardiac arrest (Dean et al., 2019), a finding that supports the hypothesized role of endogenous DMT in NDEs (Strassman, 2000; Timmermann et al., 2018). The specificity of this release needs to be considered, however, given that concentrations of 5-HT and other neurotransmitters are also massively increased during asphyxiation, cardiac arrest and hypercapnia and may compete with DMT at the 5-HT2AR (Johnson et al., 2005; Li et al., 2015; Nichols and Nichols, 2019). Stimulation of the 5-HT2AR may also precipitate psychotic symptoms during panic attacks (Galynker et al., 1996; Goodwin and Davidson, 2002; Masdrakis et al., 2017), which often involve a physiological disturbance (e.g. hypercapnia) coupled with cognitive misinterpretation (Clark, 1986; Vollmer et al., 2015). Preliminary evidence suggests the 5-HT2AR mediates CO_2_-induced arousal (Buchanan et al., 2015; Smith et al., 2018), a popular model of panic attack, as well as anxiety responses to corticotrophin-releasing factor receptor 1 stimulation (Magalhaes et al., 2010). That CO_2_ inhalation can induce panic or pleasant psychedelic-like experiences (Meduna, 1950), with activation of 5-HT2ARs likely playing a role, is supportive of the PiMS model, including its emphasis on the context dependency of outcomes.

Metabolic dysfunction and starvation are associated with psychotic phenotypes (Morylowska-Topolska et al., 2017; Pillinger et al., 2017; Prabakaran et al., 2004). Chronic tryptophan depletion (3 weeks) selectively increases cortical 5-HT2AR binding (Cahir et al., 2007). Fasting also increases serotonin-induced intracellular calcium cation concentration, a proposed correlate of 5-HT2AR function in the brain (Sudo et al., 1997), as stimulation of 5-HT2ARs induces intracellular calcium release (Raote et al., 2007). Increases in cortical brain-derived neurotrophic factor (BDNF), as well as the antidepressant and anti-inflammatory effects of fasting (Cui et al., 2018; Fond et al., 2013), are consistent with the effects of 5-HT2AR activation (Carhart-Harris et al., 2016a; Cui et al., 2018; Flanagan et al., 2019b; Jaggar and Vaidya, 2018). Acute fasting and intermittent religious fasting have been shown to increase 5-HT levels and metabolism (Bastani et al., 2017; Fuenmayor and Garcia, 1984; Ishida et al., 1997; although see Bubenik et al., 1992) whereas longer-term tryptophan depletion or starvation may decrease 5-HT levels (Cahir et al., 2007; Haider and Haleem, 2000). Agonists and antagonists of the 5-HT2AR reliably decrease and increase feeding, respectively (see Gorwood et al., 2018 for review).

There is some evidence of increased 5-HT2AR expression in overweight individuals (Erritzoe et al., 2008; although see Chaouloff et al., 1995) and reduced 5-HT2AR expression in anorexia nervosa (AN) (Audenaert et al., 2003; Bailer et al., 2004; Frank et al., 2002; Kaye et al., 2013; although see Bailer et al., 2007). That patients recovered from AN continue to display reduced 5-HT2AR expression (Kaye et al., 2013) might be interpreted as suggesting that decreased 5-HT2AR expression is an inherited trait marker of AN, but we suggest a compensatory long-term downregulation of 5-HT2ARs in AN could also occur in response to chronic overactivation of 5-HT2ARs associated with restricted feeding. Enduring but state-specific decreases in 5-HT2AR expression, as well as epigenetic modifications of the 5-HT2AR, have been observed in schizophrenia (Abdolmaleky et al., 2011; Cheah et al., 2017; Rasmussen et al., 2016); see below.

Sleep deprivation can serve as a model of psychosis and the relationship between sleep deprivation/disorders and psychosis is well established (Meyhöfer et al., 2017; Reeve et al., 2018; Waters et al., 2018). Sleep deprivation is associated with a rapid upregulation of 5-HT2AR expression in rodents (Maple et al., 2015; Zhao et al., 2019) and increased cortical 5-HT2AR binding in humans (Elmenhorst et al., 2012). Antagonists and agonists of the 5-HT2AR promote sleep and wakefulness, respectively (see Monti et al., 2018 for review), and both increases and decreases in 5-HT levels following sleep deprivation have been observed (see Menon et al., 2019 for review), although increased 5-HT levels may be more likely if combined with malnourishment (Alfaro-Rodríguez et al., 2006).

Severe and chronic pain and pain-processing abnormalities are associated with psychosis (Koyanagi and Stickley, 2015; Minichino et al., 2016). The function of 5-HT2AR is involved in nociception and antinociceptive response in peripheral tissue, spine and brain in ways that are too nuanced for generalizations or coverage here (Abbott et al., 1996; da Silva Soares et al., 2019; Seyrek et al., 2010; Zuo et al., 2019; see Cortes-Altamirano et al., 2018; Courteix et al., 2018 for review). Acute pain quite reliably increases 5-HT release (see Neugebauer, 2020 for review of serotonin modulation of pain) and increased 5-HT2AR binding in various cortical areas is associated with tonic pain ratings in humans (Kupers et al., 2009).

The 5-HT2AR also plays a role in body temperature regulation, with agonists reliably increasing core body temperature (Blessing and Seaman, 2003; Friedman and Hirsch, 1971; Klock et al., 1975; Liskow, 1971; Mazzola-Pomietto et al., 1995, Murakami et al., 1980; Pawlyk et al., 2006; Salmi and Ahlenius, 1998) and antagonists blocking this effect (Mazzola-Pomietto et al., 1995; Nisijima et al., 2001). Antagonism of the 5-HT2AR also reduces body temperature (Pawlyk et al., 2006) and blocks hyperthermic response to social defeat (Sinh and Ootsuka, 2019). Some evidence suggests that exposure to heat or cold increases 5-HT levels (Hale et al., 2011; Ishiwata and Greenwood, 2018; Kelly et al., 2011; Myers and Beleslin, 1971), but other studies found no relationship between temperature manipulations and brain 5-HT levels (Ishiwata et al., 2004; Nakagawa et al., 2016; Saito et al., 2005). We are not aware of any direct evidence showing that body temperature manipulation influences 5-HT2AR activity (Zamfir et al., 1992), but given the intentional manipulation of body temperature in spiritual and therapeutic practice (see below), there are logical reasons to suspect there may be a relationship.

Preliminary evidence suggests that decreased brain pH is an endophenotype for schizophrenia and bipolar disorders (see Hagihara et al., 2018 for review). Acidosis (low pH) could be caused by hypercapnia due to respiratory complications, high altitude, breathing techniques, as well as ketoacidosis associated with starvation, metabolic dysregulation and alcoholism. It has been proposed that brainstem and midbrain 5-HT neurons act as chemoreceptors sensitive to extracellular pH (Richerson, 2004; Teran and Richerson, 2020), potentially explaining how homeostatic imbalance caused by stress might generally engage the serotonergic system and elicit psychedelic-like subjective effects via heightened 5-HT2AR agonism. Experimentally exploring this relationship, for example by testing the capacity of a 5-HT2AR antagonist to block the effects of putative CO_2_-induced psychedelic-like experience (Meduna, 1950), would be a relevant and potentially fruitful future research avenue for the PiMS hypothesis.

The link between stress and psychosis is well established and consistent with the ‘diathesis-stress’ model of psychopathology in which pre-existing vulnerabilities (diathesis = predisposition) combine with stress to catalyse transition into illness (Belsky and Pluess, 2009). The causal link between stress and spiritual experience may be less obvious – but it is, in fact, supported by a wealth of evidence – as we shall see more clearly in the next section. In summary, a large variety of chronic and acute intense stressors upregulate and activate the 5-HT2AR and are associated with PiMS-relevant phenomena.

### Intentional stress-induced pivotal mental states?

Is it possible that humans have intuited how to hijack or ‘hack’ their own physiology for the purpose of self-development? Asceticism, or the withdrawal from sensory stimulation and dedication to a simple but disciplined lifestyle, has an ancient history of association with altered states of consciousness (Hof and Rosales, 2011; Kotler and Wheal, 2017; Wimbush and Valantasis, 2002). Our perspective is that self-manipulated and intended ‘stress’ leading to increased 5-HT2AR signaling and associated PiMSs is a relevant candidate mechanism here. Various evidence supporting this view is provided below.

Intentional social isolation, often in nature, is associated with spiritual and transformative experiences (Naor and Mayseless, 2017). Many religious narratives report that religious exemplars (e.g. Moses, Jesus, Mohammed, Siddhartha, Lao Tzu) sought extreme solitude – often in conjunction with the discovery of their benchmark philosophies. With regard to cognitive stressors, meditation on sin, guilt, death and suffering play a role in the spiritual exercises of various religious traditions (Giustarini, 2012; Loyola, 2007). Celibacy is another ascetic practice (Olson, 2008) that may create tension and stress.

Fasting, or extreme moderation of food intake, is a common religious practice (Diamond, 2003; Dugan, 1995; Eskildsen, 1998; Laidlaw, 2005; Sakr, 1975). Bouts of extreme fasting, such as those reportedly undertaken by Jesus in the wilderness or Prince Siddhartha (the ‘Buddha’) before enlightenment, often precede important spiritual or religious revelations. Spiritual ‘athletes’ practice sleep restriction, promote night-time and/or early morning spiritual exercises, and in extreme cases, physically disable themselves from lying down (Farré-i-Barril, 2012; Macmillan, 2013). Endurance activities, such as the epic kaihōgyō (1000 marathons in 1000 days) or sun dance (Rhodes, 1987; Spier, 1921), are sometimes used for spiritual development, as is self-inflicted bodily pain and self-mortification (Camporesi, 1988). Manipulations of respiration and body temperature, as evidenced by yogic breathing (Brown and Gerbarg, 2005) and the Native American sweat lodge (Garrett et al., 2011), also play roles in spiritual practices and ceremonies.

Modern therapeutic techniques such as breathwork (Brown and Gerbarg, 2005; Grof and Grof, 2010) and whole-body hyperthermia (Hanusch et al., 2013; Janssen et al., 2016) continue to reinforce the idea that physiological stress or dysregulation can lead to meaningful changes in mental states and have been found to interface with the serotonin system (Anju et al., 2011; Buchanan et al., 2015; Pawlyk et al., 2006). The cross-cultural and recurring practice of asceticism cannot be explained as a purely culturally contingent phenomenon. As reviewed by Singh (2018) and noted by Winkelman and White (1987), ‘shamans’ (or their cultural equivalents), in various societies, engage in ascetic practices such as social isolation, food, sleep and sex restriction. Combining multiple ascetic practices with meditation, prayer or ritual and a background of stress may (perhaps inadvertently) create ideal synergistic conditions for the natural upregulation of 5-HT2AR signaling (Alfaro-Rodríguez et al., 2006) and thus, the emergence of a PiMS.

A crucial distinction between ascetic practices and unintentional counterparts, such as social exclusion or involuntary celibacy, is that the ascetic retains a sense of control over the stressors and a willingness towards self-transformation. Although some ascetics may be predisposed towards harm avoidance in the domains of social interaction or sexual relationships, for example, a positive estimation of this behaviour m0ay be protective against the derogatory and persecutory evaluations so integral to psychosis. This raises the important question of whether ascetic behaviour directly drives the occurrence of PiMSs, or whether the onset of PiMS biases a person towards social withdrawal, celibacy, starvation, as well as other behaviours associated with asceticism. As is often the case with such ‘chicken and egg’ type questions, the dilemma may be solved by invoking bidirectional causality.

While acknowledging the relevant causal pathways may operate bidirectionally and be mutually reinforcing, additional evidence for ascetic-like behaviour being causative of PiMSs is provided by secular uses of stress to induce altered states. For example, militaries regularly subject new recruits to psychosocial and physiological stressors in efforts to break down and reform the individual into a community-minded soldier (McGurk et al., 2006). It is also debated whether various forms of torture involving sleep deprivation, social isolation and pain may render an individual more suggestible to the planting and/or imprinting of new/false beliefs and memories (Bloche, 2016; O’Mara, 2015). Indeed, it seems logical, given the already cited associations between stress and the 5-HT2AR, that such torture techniques would elevate brain plasticity via upregulation and activation of 5-HT2ARs (Ly et al., 2018). It is therefore relevant and noteworthy that a significant history exists of combining torture, coercion and interrogation techniques with the administration of psychedelic drugs such as LSD (Lee and Shlain, 1992), which are known to promote suggestibility (Carhart-Harris et al., 2015).

Returning to the question at the beginning of this section, there are good reasons to surmise that, at some given period in our evolutionary development, a perhaps universal intuition arose amongst humans that physiological manipulation can induce hyper-plastic mind and brain states; a realization that has since been exploited throughout the ages for purposes of self-development and spiritual or religious growth (Kotler and Wheal, 2017). In the next section, we will begin to address in more detail some candidate physiological and neuropharmacological processes that are likely to serve an important causal and/or mediational role in the induction of PiMSs. Given the special reliability with which they can induce PiMSs, we focus on the action of psychedelic drugs.

## Psychedelics, pivotal mental states and the 5-HT2AR

### Psychedelics, psychopathology and spiritual experiences

As discussed earlier, certain psychotic states (e.g. early and acute psychosis) are considered important examples of naturally occurring PiMSs. The psychotomimetic (psychosis-mimicking) effects of classic 5-HT2AR agonist psychedelics have been well documented (Carhart-Harris et al., 2016b; Gonzalez-Maeso and Sealfon, 2009; Quednow et al., 2020; Vollenweider et al., 1997, 1998). Importantly, psychedelics are felt to be useful models of *incipient* psychotic states that may be more likely to display psychedelic-like phenomena (Bowers and Freedman, 1966), such as changes in perception, cognition and ego functioning (Bercel et al., 1956; Bowers and Freedman, 1966; Carhart-Harris et al., 2013; Fischman, 1983; Gouzoulis-Mayfrank et al., 2005; Savage, 1955; Savage and Cholden, 1956). Conversely, established psychotic disorders such as schizophrenia are more likely to feature characteristics of *rigid* cognition such as fixed delusions (Paolini et al., 2016; Rajapakse et al., 2011). Selective 5-HT2AR antagonism attenuates the main characteristic subjective effects of LSD, psilocybin and ayahuasca (Preller et al., 2017; Quednow et al., 2012) and the intensity of psychedelic states is reliably predicted by 5-HT2AR occupancy (Madsen et al., 2019).

Beyond the hypothesized involvement of the 5-HT2AR in the induction of psychotic states (Carhart-Harris et al., 2014b; Geyer and Vollenweider, 2008), it is generally thought that dopaminergic (DA) dysregulation serves as a ‘final common pathway’ underlying chronic psychoses (Howes and Kapur, 2009). In support of this notion, tolerance to sustained 5-HT2AR agonism develops quickly (Geyer and Vollenweider, 2008) and more selective 5-HT2AR antagonists lacking any appreciable DA antagonism properties have not proven to be efficacious antipsychotics (De Paulis, 2001; Garay et al., 2016; Meltzer et al., 2004). However, some evidence does suggest that 5-HT2AR binding and blockade may contribute to the superior efficacy of atypical antipsychotics (particularly clozapine) in attenuating positive and negative symptoms (Aringhieri et al., 2018, Meltzer and Massey, 2011; Richtand et al., 2007). Pimavanserin, a selective 5-HT2AR inverse agonist, has been licensed for psychotic symptoms in relation to Parkinson’s disease psychosis (Cummings et al., 2014; Mohanty et al., 2019), with some additional evidence of efficacy for psychotic symptoms in Alzheimer’s disease (Ballard et al., 2019) and schizophrenic psychoses unresponsive to clozapine (Nasrallah et al., 2019). It has also been demonstrated that, via antagonism of the 5-HT2AR, clozapine ameliorates the psychotomimetic effects of N-methyl-D-aspartate receptor (NMDAR) antagonism in animal models (Schmid et al., 2014, Szlachta et al., 2017). Similar findings have also been observed with risperidone (which has appreciable 5-HT2AR antagonist properties) and ketamine (an NMDAR antagonist) in humans (Joules et al., 2015); see (Carhart-Harris et al., 2013) for a critique of NMDAR antagonist drug models of psychosis.

Serotonin 2A antagonism has less marked effects on cognition than 5-HT2AR agonism but there is some evidence it can impair learning (Welsh et al., 1998; Zhang and Stackman, 2015) and promote compulsive behaviour (Kim et al., 2019; Schirmbeck and Zink, 2012) as well as sleep (Monti et al., 2018). Thus, it is possible that some of the therapeutic effects of 5-HT2AR antagonist antipsychotics may be due to a generic negative action on learning-related cognition and wakefulness. Given the central thesis of this paper, the deployment of 5-HT2AR antagonists early in the etiology of a psychotic disorder may conceivably prevent conversion to psychosis via suppressing the occurrence of a mediating pre- or peri-psychotic PiMS. Consistently, 5-HT2AR antagonism has been found to blunt or reduce cortical plasticity (Inaba et al., 2009; Jitsuki et al., 2011; Lombaert et al., 2018; Xu et al., 2012). Whether such actions have net positive or negative repercussions for long-term mental health is a complicated question, but see (Whitaker, 2011) for an interesting and relevant perspective.

Most post-mortem studies that have assessed 5-HT2AR levels have reported downregulated 5-HT2AR mRNA and protein expression in the cortex of individuals who had schizophrenia, and *in vivo* imaging of 5-HT2AR binding in schizophrenia also suggests decreased cortical receptor densities (see Quednow et al., 2020 for review). Decreased 5-HT2AR expression in schizophrenia may be state specific, as decreased 5-HT2AR binding in first-episode psychosis correlates with severity of positive symptoms (Rasmussen et al., 2010) and healthy monozygotic siblings of affected persons do not display decreases in 5-HT2AR binding (Rasmussen et al., 2016). It is plausible that in prolonged psychoses, the 5-HT2AR becomes adaptively downregulated after an initial period of heightened activity, consistent with agonist-induced downregulation of the 5-HT2AR (Erritzoe et al., 2011). Evidence of epigenetic modification of the 5-HT2AR in schizophrenia (Abdolmaleky et al., 2011; Cheah et al., 2017) is also relevant here.

It seems plausible that a cortical abnormality mediated by increased 5-HT2AR signaling and related glutamatergic activity (Aghajanian and Marek, 2000; Moreno and González-Maeso, 2018) and featuring abnormal plasticity (Kavanagh et al., 2015; Stephan et al., 2009) and associative learning – as mediators of major psychological change – is an important early component of the psychotic process in schizophrenia; whereas 5-HT2AR-mediated dysregulation of dopamine activity is a consequential, and perhaps defining component, namely ‘the final common pathway’ (Howes and Kapur, 2009; Pehek et al., 2006; Pehek and Hernan, 2015; Stahl, 2018). If we allow ourselves to be instructed on the pharmacology of the psychotic process via its phenomenology, then an initial state characterized by ego-disturbance and cognitive and perceptual disturbance preceding subsequent inflexible or perseverative cognitive and behavioural styles (Boulougouris et al., 2008; King et al., 1974; Murphy-Beiner and Soar, 2020) might fit with an initial serotonergic (5-HT2AR) component (although see Boulougouris et al., 2008), followed by a final pathway that is dominated by a hyperactive mesolimbic dopamine system (Stahl, 2018). However, converging evidence also suggests that upregulation (Chiu et al., 2014; Hámor et al., 2018; Napier and Istre, 2008), sensitization (Chiu et al., 2014; Hámor et al., 2018; Napier and Istre, 2008), and direct agonism (Soman et al., 2019) of 5-HT2AR contributes to dopamine-induced psychoses (Cummings et al., 2014; Meltzer et al., 2010). Indeed, manic states might precipitate incipient psychoses in a similar fashion (Correll et al., 2007; Jauhar et al., 2017). It may be relevant in this sense that some shared features have been found in the phenomenology of spiritual experiences and positive psychotic symptoms and the psilocybin experience whereas manic symptoms appear to be mimicked more reliably by pro-DA stimulant drug experiences (Carhart-Harris et al., 2013).

Beyond psychosis, psychedelics offer a reliable model of ‘peak’ (Roseman et al., 2018), ‘god-encounter’ (Griffiths et al., 2019), ‘mystical-type’ (Griffiths et al., 2006, 2008, 2011), near-death (Strassman, 2000; Timmermann et al., 2018) and other such anomalous experiences (Carbonaro et al., 2017) that bear an undeniable resemblance to experiences designated as ‘religious’ or ‘spiritual’. Trait absorption is one of the most reliable predictors of sensitivity to the subjective effects of psychedelics (Haijen et al., 2018) and has also been found to predict spiritual experiences induced via various means (Lifshitz et al., 2019). The 5-HT2AR rs6313 TT genotype is associated with trait absorption and altered time-perception in humans, both of which are implicated in spiritual experiences and NDEs (Cant et al., 2012; Ott et al., 2005; Sysoeva et al., 2010). NDEs and psychedelic experiences feature ‘time-dilation’ (Yanakieva et al., 2019) and altered time perception has also been reported in psychotic states (see Thoenes and Oberfeld, 2017 for review) and PTSD (Ahmadi et al., 2019).

### The psychology of the psychedelic state

We begin this section with a focus on the psychology of the psychedelic experience before turning our attention to its neurobiology, with specific reference to human brain imaging studies with psychedelics. Psychedelics induce cognitive-perceptual instability, relax one’s normal sense of agency and ownership over perceptual objects, including one’s own body and at higher doses, reliably produce different degrees of ‘ego-dissolution’ (Millière, 2017; Nour et al., 2017). Psychedelics also elicit an increased emotional lability – not inconsistent with infancy, early psychosis and spiritual experiences (Carhart-Harris et al., 2016b). The recently developed RElaxed Beliefs Under pSychedelics (REBUS) model proposes that, via a 5-HT2AR-induced entropic effect on cortical activity (Carhart-Harris and Friston, 2019), psychedelics relax the precision weighting (i.e. inverse variance) of high-level priors (internal predictive models), thereby allowing bottom-up information (‘prediction error’) to flow more freely up the brain’s functional hierarchy to impress on high-level cortices and enter conscious awareness. It is proposed that this process is necessary for the effective revision of priors or beliefs (Carhart-Harris and Friston, 2019). This model has recently received support from the application of travelling wave analyses to DMT electroencephalogram (EEG) data, where a rapid shift from top-down to bottom-up dominating waves coincided very closely the onset and subjective intensity of the DMT experience (Alamia et al., 2020).

The REBUS model is consistent with much of the phenomenology of the psychedelic experience, including intense spiritual, ‘peak’ or ‘mystical-type’ experiences (see Barrett and Griffiths, 2017; Roseman et al., 2018). In these (typically high-dose) experiences, discriminative beliefs (e.g. A is different to B) are often moderated and replaced by a sense of reciprocal interconnectedness, which is referred to as the ‘unitive experience’ in studies of ‘mystical’ or ‘spiritual’ experiences (Stace, 1960). Such experiences may account for lasting psychological changes seen with psychedelics, such as enduring increases in the personality trait ‘openness’ (Erritzoe et al., 2018; MacLean et al., 2011; Nour et al., 2017) as well as improvements in wellbeing (Haijen et al., 2018). Thus, during and after profound psychedelic experiences, specific beliefs and the emotional valences attached to them seem particularly susceptible to change, consistent with so-called ‘quantum change experiences’ (C’De Baca and Wilbourne, 2004; Miller and C’de Baca, 2001).

Returning to the phenomenology of the psychedelic state, peak, mystical-type or spiritual experiences and associated feelings of oceanic boundlessness have been found to reliably predict positive outcomes in psychedelic therapy (Griffiths et al., 2016; Haijen et al., 2018; Roseman et al., 2018; Ross et al., 2016). However, they may also represent an extreme experience only achievable (to most people) through pharmacological manipulation. Achieving the same quality and intensity of experience may be difficult via spiritual practice alone; although a recent study comparing the interaction between psychedelics and meditation has suggested a potential synergistic effect (Smigielski et al., 2019; see also Millière et al., 2018). Given our focus on stress-induced PiMSs and associated psychological transformation, it is worth taking a closer look at the common themes of psychological struggle and breakthrough in psychedelic experience (Roseman et al., 2019).

Through the relinquishment of top-down control effected by high-level priors (Carhart-Harris and Friston, 2019), psychedelics may free suppressed emotions and memories so they may more easily percolate into consciousness awareness (Alamia et al., 2020; Carhart-Harris et al., 2014a). Such emotions and memories may be felt as being emotionally challenging (Barrett et al., 2016). Feelings of anxiety are common during and after psychedelic experiences (Barrett et al., 2016; Belser et al., 2017; Carbonaro et al., 2016; Eisner and Cohen, 1958; Gasser et al., 2015; Roseman et al., 2018, 2019) as are symbolic/archetypal themes (Hill, 2013; Malone et al., 2018; Masters and Houston, 2000 [1966]; Shanon, 2002). Intense personal and transpersonal themes can rise to the forefront of awareness (Belser et al., 2017). Values can be ‘remembered’ (Belser et al., 2017) and affect-laden beliefs that previously seemed ‘abstract’ (e.g. ‘love is fundamental’) can be deeply felt (Belser et al., 2017; Pollan, 2019). Cathartic release under psychedelics (Roseman et al., 2018, 2019) may foster an emotional re-evaluation of cognitive (Lyons and Carhart-Harris, 2018b) and philosophical perspectives (Lyons and Carhart-Harris, 2018a) that may have previously been closely tied in with a person’s pathology.

Appropriately managed, psychedelic experiences can lead to an increased willingness and an ability to engage with emotionally difficult psychological material (Roseman et al., 2018; Watts et al., 2017). In therapeutic contexts, this experience is generally positive and is associated with an enhanced sense of emotional empathy (Dolder et al., 2016; Pokorny et al., 2017) and pro-social feelings and behaviour (Griffiths et al., 2018). It warrants stating, however, that extreme negative affect, including paranoid ideation and occasional injurious behaviour, can also arise during psychedelic experiences (Carbonaro et al., 2016; Coid et al., 2016; Honings et al., 2016; Strassman, 1984), particularly if the contextual frame in which the experience occurs is not sufficiently well controlled and supportive.

### The long-term psychological effects of psychedelics

The following psychological factors have been found to be altered in an enduring way with psychedelics: (a) personality (Erritzoe et al., 2018; Lebedev et al., 2016; MacLean et al., 2011); (b) mental health and wellbeing (Argento et al., 2017; Bogenschutz et al., 2015; Carhart-Harris et al., 2017, 2018b; Gasser et al., 2015; Griffiths et al., 2006, 2008; Haijen et al., 2018; Hendricks et al., 2015; Johnson et al., 2014; Osório et al., 2015; Moreno et al., 2006; Ross et al., 2016); (c) political perspective (Lyons and Carhart-Harris, 2018a; Nour et al., 2017); (d) lifestyle preferences (Forstmann and Sagioglou, 2017); and (e) feelings towards the environment/nature (Forstmann and Sagioglou, 2017; Lyons and Carhart-Harris, 2018a; Kettner et al., 2019).

### The neurobiological effects of psychedelics

Functional brain imaging studies of the acute psychedelic state using a variety of psychedelics have observed decreases in the functional modularity of a broad range of functional modules, including high-level networks such as the default-mode and fronto-parietal control network (Carhart-Harris et al., 2016c; Lebedev et al., 2015). Moreover, decreases in the integrity of these networks (Carhart-Harris et al., 2016c; Lebedev et al., 2015) as well as their reduced presence at any given point in time (Lord et al., 2019) appear to relate to high-level aspects of the drug-induced subjective experience, including ‘ego-dissolution’. Under psychedelics, the normal functional segregation or specialization in the brain is instead replaced by a globally interconnected profile (Carhart-Harris et al., 2012a, 2016c; Lord et al., 2019; Petri et al., 2014; Preller et al., 2018; Smigielski et al., 2019; Tagliazucchi et al., 2014). It is natural to speculate that this increase in global connectivity in the brain under psychedelics relates to the weakening of discriminative cognition (e.g. A versus B), exemplified by the unitive experience as well as ‘non-dual awareness’. It is easy to see how something akin to the unitive experience may occur in early psychosis, in which compromised ego-boundaries are reported (Carhart-Harris et al., 2012b; Parnas and Handest, 2003) and similar changes in functional connectivity have been seen in early psychosis as with psychedelics (Carhart-Harris et al., 2013). Decreased orthogonality between high-level networks (e.g. the default mode and salience networks) may also be a sub-acute effect of ayahuasca (Pasquini et al., 2020), although this effect has not been seen consistently (sub-acutely) with psilocybin (Barrett et al., 2020; Carhart-Harris et al., 2017).

Psychedelics have been shown to promote extinction-learning through 5-HT2AR agonism (Cameron et al., 2018; Catlow et al., 2013; Young et al., 2017) as well as low-level associative learning (Gimpl et al., 1979; Harvey, 1996, 2003; King et al., 1974; Morici et al., 2018; Romano et al., 2010; Welsh et al., 1998; Zhang et al., 2013, 2016a; Zhang and Stackman, 2015), for example, as has been nicely demonstrated by the catalysing influence of LSD (a 5-HT2AR agonist) on learning rate versus the impairing influence of ritanserin, a 5-HT2AR antagonist (Harvey, 2003).

Consistently, psychedelics have been shown to potently increase cortical neuroplasticity (Benekareddy et al., 2012; Cavus and Duman, 2003; Frankel and Cunningham, 2002; Gewirtz et al., 2002; Jaggar and Vaidya, 2018; Jones et al., 2009; Meller et al., 2002; Niitsu et al., 1995; Vaidya et al., 1997). A doubling of BDNF mRNA was found in the parietal cortex after administration of the 5-HT2AR agonist DOI (Vaidya et al., 1997) and markedly increased functional and structural neuroplasticity has been found after DMT, LSD and 2,5-Dimethoxy-4-iodamphetamine (DOI) (Ly et al., 2018) – leading the senior author to introduce the term ‘psychoplastogen’ for these and any other relevant compounds that can rapidly elicit appreciable increases in neuroplasticity (Olson, 2018). We have preliminary evidence of increased visual evoked long-term potentiation (LTP) in healthy individuals under the influence of psilocybin (Olson, 2018), and long-term changes in network functionality have also been observed 1 day (Carhart-Harris et al., 2017), 1 week (Barrett et al., 2020) and 1 month after psilocybin (Lyons, 2020) in clinical (Carhart-Harris et al., 2017) and non-clinical populations (Carhart-Harris et al., 2012a).

Increased (spontaneous) brain (activity) entropy or complexity is a highly reliable marker of the acute functional brain effect of psychedelics (Abasolo et al., 2015; Carhart-Harris, 2018b; Dolan et al., 2018; Fernández et al., 2013; Lebedev et al., 2016; Lyons, 2020; Vivot et al., 2020). Increased brain complexity has also been observed during rapid eye movement (REM) sleep (Abasolo et al., 2015), meditation (Vivot et al., 2020), musical experiences (Dolan et al., 2018) and certain psychotic states, with medication status, age and the stage of the psychotic process the key parameters to consider when assessing this (Fernández et al., 2013).

Acute brain entropy under LSD, measured with functional magnetic resonance imaging (fMRI), was found to predict psychological changes 2 weeks later, namely increased trait ‘openness’ (Lebedev et al., 2016) and in a more recent study, increased brain complexity under psilocybin was found to predict both long-term changes (1 month later) in brain network functional connectivity (Lyons, 2020) as well as related improvements in wellbeing (Lyons, 2020). Future neuroimaging analyses of ours will assess how acute brain complexity relates to functional proxies of LTP as well as long-term anatomical changes in the human brain. It is tempting to speculate that the acute brain changes seen with psychedelics, including increased complexity or entropy, are reflective of a hyper-plastic state that mediates subsequent functional and potentially structural brain changes that correlate with long-term psychological changes.

## The adaptive function of pivotal mental states

*How could you rise anew if you have not first become ashes*.

(Nietzsche, Thus Spoke Zarathustra)

As discussed in previous sections, certain types of stress upregulate 5-HT2AR expression and activity (see Table 1 for a review of the relevant literature). Moreover, evidence that acute stress causes the release of 5-HT is compelling (Adell et al., 1997; Amat et al., 1998, 2005; Bastani et al., 2017; Beitia et al., 2005; Bland et al., 2003; Cohen et al., 2015; Ferres-Coy et al., 2013; Fuenmayor and Garcia, 1984; Fujino et al., 2002; Gardner et al., 2005; Hale et al., 2011; Higuchi et al., 2019; Ishida et al., 1997; Ishiwata and Greenwood, 2018; Johnson et al., 2005; Keeney et al., 2006; Kelly et al., 2011; Li et al., 2015; Myers and Beleslin, 1971; Nakajima et al., 2009; Neugebauer, 2020; Paul et al., 2011; Rex et al., 2005; Yoshioka et al., 1995) and it is logical to surmise that increased endogenous 5-HT release will engage the 5-HT2AR system, an assumption backed up by evidence of increased 5-HT2AR associated responses with drug-induced 5-HT release (Kuypers et al., 2018; Liechti et. al., 2000; Pitts et al., 2017; van Wel et al., 2012). These factors have led us (Carhart-Harris and Nutt, 2017) and others (Murnane, 2019) to speculate that the 5-HT2AR system is a stress response system that services adaptability.

Natural questions that follow from this model include why does this function exist and what evolutionary purpose does it serve? Our proposal is that PiMSs have evolved to allow the experiencer a psychological ‘fresh start’, akin to a psychological ‘rebirth’ (Brodersen and Glock, 2016; White, 2004) or allostatic recalibration (Ellis and Del Giudice, 2019). Rather than simply referring to adaptive responses to stress and adversity, which could include many of the symptoms of psychiatric disorders, the PiMS model is intended to invoke the idea that such adaptation can be radical, rapid and discrete, rather than moderate, slow and continuous. These ideas are at least partially consistent with the notion of allostasis (McEwen, 2019; Sterling and Eyer, 1988) and the adaptive calibration model of stress response (Ellis and Del Giudice, 2019), which, like the PiMS model, refuses to characterize adaptive responses to stress as necessarily ‘toxic’ or pathological.

Thus, according to the PiMS model, the outcome of an allostatic recalibration process can be ‘positive’ (e.g. in terms of a therapeutic or spiritual breakthrough) but the same mechanisms could just as easily result in a new or reinforced maladaptive strategy, perhaps best exemplified by a psychotic ‘flight from reality’ (Broome et al., 2007; Freeman et al., 2014; Lyon et al., 1994) or progressive reinforcement of other psychological defence mechanisms. Consistent with previous work (Carhart-Harris, 2018a; Carhart-Harris and Nutt, 2017), it was recently hypothesized that 5-HT2AR signaling forms part of a ‘stress response system’ that serves the adaptive function of reconfiguring responses to environmental stimuli co-occurring with or preceding major perceived existential threats or crises (Murnane, 2019). Thus, the principle is that accelerating learning (Harvey, 2003) and extinction learning or ‘unlearning’ (Cameron et al., 2018) in such extreme situations should aid the individual in the future by causing a domain-general ‘overhaul’ in their outlook.

Evidence of aberrant neuroplasticity in psychosis (Stephan et al., 2009), including both increases (Yee et al., 2018) and decreases (Javitt et al., 1996; Umbricht and Krljes, 2005) in neuroplasticity markers, for example, particularly during critical developmental windows, may bear relevance to the reliable increases in plasticity linked to 5-HT2AR agonism (Ly et al., 2018; Olson, 2018), which are linked here with the PiMS construct. As discussed above, one way in which apparent inconsistencies in the literature may be reconciled is to consider *process* relevant dynamics, where, during a process, a phase of upregulation in a particular parameter (e.g. 5-HT2AR signaling and associated plasticity) can be followed by a phase of adaptive downregulation is the same parameter (e.g. decreased 5-HT2AR expression). Indeed, agonist-induced downregulation of the 5-HT2AR is entirely consistent with this principle (Bull et al., 2004; Erritzoe et al., 2011; Muguruza et al., 2014; Reneman et al., 2002; also see Madsen et al., 2020). From a neurodevelopmental perspective, we must also acknowledge that hyperactivation of 5-HT2ARs coinciding with childhood stress or chronic drug-use *could* negatively affect neurodevelopmental processes. Again, however, the influence of context here must be considered.

Near-death experiences are relevant to the theory that the 5-HT2AR system has evolved to aid psychological transformation for adaptive ends. Altered perceptual processing, heightened emotional tone, time dilation and enhanced episodic memory encoding are all features of NDEs. The major shifts in perspective following NDEs may be the product of a period of hyper-plasticity, potentially mediated by either the massive release of 5-HT or indeed endogenous 5-HT2AR agonists such as DMT during the dying process (Dean et al., 2019). One view is that NDE, and associated hyper-plasticity, may function to aid the individual if such life-or-death-type scenarios were to be met with again in the future. However, another, not incompatible view, is that the dramatic cognitive set-shifts seen after NDEs reflect a more fundamental ‘opening-up’ of high-level mental schemata to revision, consistent with a generic recalibration process and the recent REBUS model (Carhart-Harris and Friston, 2019). Major cognitive revisions may enable an individual to ‘start afresh’ in both the behavioural and cognitive sense, with a refined and recalibrated (‘judgement light’) perspective (Carhart-Harris and Friston, 2019). The same functional explanation could also be used in reference to ‘post-traumatic growth’, a construct that, at least by name, is only two decades old (Tedeschi, 1999). The notions of allostasis (McEwen, 2019; Sterling and Eyer, 1988) and ‘adaptive plasticity’ and ‘adaptive calibration’ (see Ellis and Del Giudice, 2019 for review) are also relevant here.

A relevant recent perspective on the evolutionary origin and potential treatment of PTSD (Murnane, 2019) is largely consistent with an earlier perspective piece on 5-HT2AR-mediated ‘active coping’ and radical adaptation proposed by Carhart-Harris and Nutt (2017), as well as animal work linking serotonergic functioning to faster learning rates (Harvey, 2003; Iigaya et al., 2018), plasticity (Iigaya et al., 2018) and adaptability (Matias et al., 2017). It is also consistent with a growing literature base supporting the value of classic (5-HT2AR agonist) psychedelic therapy in the treatment of a broad range of psychiatric disorders (Carhart-Harris and Goodwin, 2017; Nutt et al., 2020; Rucker et al., 2018). A crucial component of the 5-HT2AR-mediated active coping model (Carhart-Harris and Nutt, 2017) was that the relationship between stress and upregulated 5-HT2AR signaling is non-linear, in the sense that once stress crosses a critical threshold (the specifics of which are presently not clear) – in terms of its severity or chronicity – there is an abrupt shift in a system’s functioning (e.g. global brain function) into a radically different mode (also consistent with bifurcation theory). The PiMS model maintains that the emergence of this mode is mediated by increased 5-HT2AR activity and associated plasticity (Olson, 2018) and its evolutionary and adaptive function is to aid radical psychological change when its need is perceived (Carhart-Harris and Nutt, 2017). Evidence for such nonlinearities in the functioning of the 5-HT2AR system do indeed exist (Dwivedi et al., 2005; Madsen et al., 2019) and should be the focus of future PiMS-relevant hypothesis testing.

Hierarchical predictive processing is an increasingly influential model of global mind and brain function that essentially posits that brains evolve and develop into predictive models of the environments they inhabit. Much of brain anatomy and function appears to be organized hierarchically (Felleman and Van, 1991; Margulies et al., 2016) and it is proposed that relevant predictive mechanisms are encoded in the brain’s multi-level functional and anatomical hierarchies (Clark, 2013; Friston, 2018; Rao and Ballard, 1999; Seth, 2013). The hierarchical predictive processing perspective has recently been applied to the action of psychedelics (i.e. in the so-called ‘REBUS’ model). This model, which is receiving growing empirical support (Alamia et al., 2020; Girn et al., 2020), argues that the de-weighting (decreased ‘precision’) of internal predictive models (‘prior probability distributions’) under psychedelics opens a window for their subsequent revision (Carhart-Harris and Friston, 2019). Consistently, it can be hypothesized here that in individuals undergoing intense PiMSs, the brain inhabits a mode of functioning that is conducive to the modulation or recalibration of internal predictive models, including how they are weighted.

Major recalibrations to fundamental beliefs or outlooks have been referred to in the psychology literature as psychological ‘transformations’ or ‘rebirths’ (White, 2004) and have received particular attention under the construct of ‘quantum change’ (C’De Baca and Wilbourne, 2004; Miller and C’de Baca, 2001). We believe the present PiMS model extends this previous (largely phenomenological) work by proposing a plausible mechanistic account for these phenomena. One may draw parallels between the so-called REBUS model (Carhart-Harris and Friston, 2019), the therapeutic application of psychedelics (Watts et al., 2017; Watts and Luoma, 2019) and the Acceptance and Commitment Therapy (ACT) psychotherapeutic model (Walsh and Thiessen, 2018), where the ACT approach endeavours to promote ‘psychological flexibility’, that is, flexible acceptance and integration of emotionally challenging memories and emotions, and seeks to do this via techniques such as ‘cognitive de-fusion’, which are exercises intended to foster an open, tolerant, inquisitive and accepting stance on psychological suffering and its causes. The possible combination of ACT or related mindfulness-based techniques with psychedelic therapy has been discussed in recent review papers (Sloshower et al., 2020; Walsh and Thiessen, 2018; Watts and Luoma, 2019) and is now being supported by empirical findings (Close et al., 2020; Davis et al., 2019).

## Therapeutic implications

The primary purpose of this review is to introduce a new psychological and neurobiological construct, the PiMS, and examine its potential causes, mechanisms and functions. PiMSs are defined by three key criteria: elevated cortical plasticity, enhanced associative learning (including extinction learning) and a special capacity to mediate psychologically transformative change. The psychedelic state can be considered a prototypical PiMSs and there is clear evidence this particular state meets all three of its defining properties (Carhart-Harris et al., 2018c; Carhart-Harris and Friston, 2019; Griffiths et al., 2006; Harvey, 1996; Ly et al., 2018). Importantly, however, it is our view that PiMSs represent a more fundamental state. We have argued that the same molecular or proteomic gateway (i.e. 5-HT2AR signalling) is implicated in non-drug, ‘naturally’ occurring PiMSs, as in the psychedelic state. Moreover, we argue that the psychological properties of psychedelics depend on their ability to hijack this natural system, which has evolved for mediating rapid, major and potentially lasting adaptive change.

In this section, we address the therapeutic implications of the PiMSs model. Clinically supervised psychedelic experiences are showing promise for the alleviation of a large number of psychiatric symptoms and unhealthy lifestyle habits (Bogenschutz and Johnson, 2016; Carhart-Harris et al., 2018a; Carhart-Harris and Goodwin, 2017; Johnson, 2018). Psychedelic therapy appears to mediate positive mental health change in a customizable and structured way, achieving a remarkable degree and reliability of therapeutic change, often via a small number of isolated therapeutic sessions, supported by subsequent psychological integration work, designed to sustain the relevant positive outcomes (Richards, 2015; Walsh and Thiessen, 2018). In contrast to the often-distressing conditions that naturally agitate the manifestation of a PiMS, psychedelic treatment primes positive transformation via the structuring of ‘set and setting’ (Carhart-Harris et al., 2018c; Hartogsohn, 2016) as part of a broader notion of ‘context’ (Carhart-Harris et al., 2018c).

**Figure 6.**
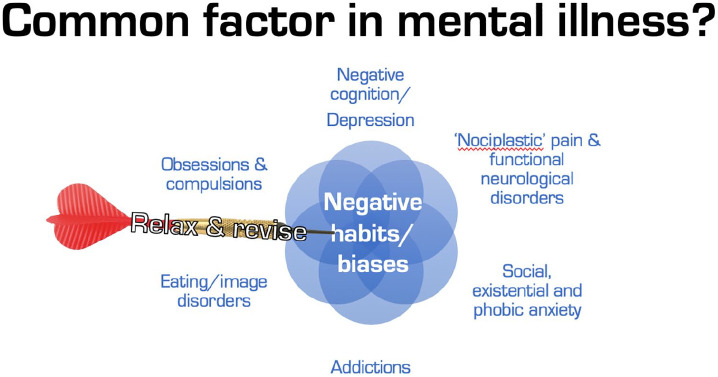
A conceptual schematic illustrating the putative transdiagnostic relevance of over-weighted internal predictive models or ‘priors’ and how they may be effectively targeted by carefully engineered pivotal mental states (PiMSs). This is represented metaphorically by a dart (serotonin 2A receptor (5-HT2AR) signaling inducing a PiMS), targeting the central space where different psychopathologies overlap. The shared characteristic of these psychopathologies is hypothesized to be over-weighted predictive models. Such over-weighted priors may be thought of as aberrant habits or biases (e.g. negative cognitive biases in depression, aberrant self-image in eating disorders or compulsive behaviours in OCD or addiction). Effective PiMS-based therapy should aim to remediate this problematic over-weighting by: (a) acutely ‘relaxing’ problematic priors and then (b) working on promoting a more permanent recalibration of beliefs or bias, for example via a commitment to a healthy behavioural change. Note: the schematic should not be (mis)interpreted as implying psychedelic therapy is a ‘cure all’.

In addition to the treatment of diagnosed psychiatric illness, present findings indicate that psychedelic therapy offers an opportunity for the de-weighting of a plethora of maladaptive cognitive/perceptual schemas or ‘sets’ about self, others and the world (Carhart-Harris and Friston, 2019; Hinton and Kimayer, 2017). This realization implies a broad, ‘transdiagnostic’ therapeutic value (Figure 6). Relatedly, data indicate the deployment of this intervention may yield benefits beyond diagnosed mental illness. For example, given observed improvements in psychological wellbeing (Jungaberle et al., 2018; Walsh, 1982) including resilience-related changes (Close et al., 2020; Davis et al., 2019), it is conceivable that psychedelic therapy could be used as a prophylactic or preventative intervention, for example, promoting adaptability in adversity (Close et al., 2020; Davis et al., 2020; Murphy Beiner and Soar, 2020). We are aware that similar inferences have been made about ACT and psychological flexibility, namely that ACT can be transdiagnostically effective and ‘psychological flexibility’ is universally relevant to mental health (Hayes, 2019).

There are (emotional) pros and cons to recognizing PiMSs as important – perhaps universally accessible – states of mind and brain and we welcome future critique of the idea. For example, it may be (emotionally) preferable to maintain strong distinctions between spiritual experiences and psychotic episodes and reject or ignore some of the similarities (DeHoff, 2015; Parnas and Henriksen, 2016). It is easy to appreciate the sensitivity of this debate: the negative stigma, prognoses and interventions that come with a diagnosis of a psychotic disorder (Wong et al., 2009) are a heavy burden, whereas evidence that spiritual experiences and religious conversions are often associated with positive mental health outcomes (Griffiths et al., 2011) argues against them being linked with pathology.

Relatedly, we anticipate some pushback to what is, essentially, a secular, naturalistic/scientific approach to phenomena others might consider ‘supernatural’, particularly given the aforementioned emotional function that these beliefs may serve (Kornfield, 2001). As discussed above with reference to predictive coding, in advocating a particular framework of belief that is meant to be held with the highest level of certainty, believers are promised an escape from an existential uncertainty (Hillen et al., 2017). Given the emotional appeal of such a prospect, it may be unsurprising that a readiness to endorse metaphysical or supernatural beliefs after a psychedelic experience is associated with (at least short-term) improvements in psychological wellbeing (Timmermann et al., 2019). However, important questions need to be asked here about the sustainability of any such effect. One should be mindful of how recourse to supernatural belief may offer the believer a means of emotional escape, consistent with the phenomenon known as ‘spiritual bypassing’ (Masters, 2010), which was also highlighted above. One must also be mindful, however, how an excessive recourse to the rationalism of science could promote a ‘cognitive fusion’ working *against* rather than *for* psychological flexibility and associated wellbeing (Hayes, 2019). The key here may be to retain a healthy and appropriate scepticism and uncertainty (e.g. of both specific scientific principles and religious dogma), balanced with respect for assumptions that have been found (e.g. via the scientific method) to be reliable and robust to scrutiny (Rovelli, 2018).

We have placed significant emphasis on the 5-HT2AR system throughout this paper, but it should be acknowledged that some prefer to extend the definition of psychedelics beyond action at this particular receptor. MDMA (Siegel, 1986) and ketamine (Bowdle et al., 1998) are two relevant examples in this regard (Kuypers et al., 2018; Lin et al., 2018; Schmid et al., 2014, Szlachta et al., 2017). Like the classic psychedelics, MDMA and ketamine have both been used as adjuncts to psychotherapy, with notable success (McGirr et al., 2017; Sessa et al., 2019; Wilkinson et al., 2017). However, the duration of the therapeutic response associated with classic (direct 5-HT2AR agonist) psychedelics (Carhart-Harris and Goodwin, 2017) appears to exceed that associated with a single exposure to ketamine and although MDMA causes an appreciable increase in activity at the 5-HT2AR through its potent release of 5-HT (van Wel et al., 2012), the non-specific nature of MDMA’s pro-serotonergic, and indeed noradrenergic and DA effects, complicates our understanding of its (pharmaco)therapeutic mechanisms (Liechti et al., 2000).

It is relevant to ask whether we feel it possible for PiMSs to occur without the involvement of increased 5-HT2AR activity. Although it is plausible to imagine this possibility, for example via a non-5-HT2AR-induced increase in cortical excitation, it also seems reasonable to assume that increased 5-HT2AR signaling may be a particularly robust and reliable inducer. One implication of this is that blocking the 5-HT2AR, as is done routinely in psychiatry via a broad range of psychiatric medications, would make PiMSs, and thus, transformative psychological change, less likely. If this is the case, it might support the fear of some that chronic medication with conventional psychiatric medication diminishes the chances of spontaneous remission and renders the medicated person vulnerable to relapse if the stabilizing medication is withdrawn (Moncrieff, 2008; Omachi and Sumiyoshi, 2018; Whitaker, 2011). Accordingly, remaining unmedicated might provide greater opportunity for self-development (Whitaker, 2011).

The obvious counterargument to this perspective, however, is that untreated psychoses render an individual more susceptible to relapse and that recurrent psychotic episodes serve to further reinforce illness chronicity (Emsley et al., 2013; Omachi and Sumiyoshi, 2018). It is also debatable whether the incipient phase of a recurrent psychotic episode that emerges inadvertently, with few warning signs (Emsley et al., 2013) and features severe paranoia and cognitive and behavioural disturbances, really represents a viable situation for (psycho)therapeutic intervention (Omachi and Sumiyoshi, 2018). Certain remote contextual factors contributing to the emergence of psychotic features, such as polygenics and or early-life trauma, might make the shepherding of a PiMS in a positive direction particularly challenging and, indeed, risky to attempt.

This debate speaks to an important cautionary point about presenting PiMSs as inherently therapeutic. Although PiMSs, by definition, represent opportunities for major change, change is not inevitable and neither are ‘positive’ outcomes necessarily more likely than negative ones. In keeping with the so-called ‘plastic paradox’ (Doidge, 2007), in the same way that states of hyper-plasticity can aid unlearning in the service of new perspectives and behaviour, they can also serve to reinforce old beliefs and biases if the relevant entraining conditions exist.

## Limitations

This paper is a theoretical piece with a narrative style. Such approaches inevitably sacrifice self-critique and counterargument in favour of placing the spotlight on supportive evidence. There are obvious limitations to this style, with a ‘cherry-picking’ confirmation bias being one notable problem. We therefore strongly encourage critiques of this work. Moreover, although we have tried to be thorough in our coverage of the literature, it not feasible to be entirely exhaustive and it is inevitable that some truncation and oversight of relevant material has occurred. For example, the perspective that psychedelics model aspects of both (early and acute) psychosis and spiritual experience is treated as largely consensual (Barrett and Griffiths, 2017; Carhart-Harris et al., 2016b; Gonzalez-Maeso and Sealfon, 2009) but either of these positions could be challenged.

This paper’s focus on a small number of example PiMSs could also be questioned. One might argue that the chosen examples are not necessarily ideal and, more specifically, that too much emphasis has been placed on psychosis in particular, ahead of other compelling alternatives, such as trauma or panic attacks. Our preferential focus on psychosis stems from: (a) the wealth of research that has been conducted on psychosis, particularly in relation to the role of the 5-HT2AR system, and (b) that it has a history of having been compared and contrasted with spiritual experiences, which is a conflict that the PiMS model bears special relevance to. Other states we could have looked at in relation to PiMSs include REM sleep (Carhart-Harris, 2007; Carhart-Harris and Nutt, 2014; Hobson et al., 2014; McNamara, 2016; Sanz et al., 2018), particularly given its phenomenological qualities (McNamara, 2019), sensitivity to 5-HT2AR manipulation (Monti et al., 2018; Monti and Jantos, 2006), evidence for its role in processing stressful scenarios (Suchecki et al., 2012) and similarities between its neural correlates and those of the psychedelic state (Carhart-Harris, 2007; Carhart-Harris and Nutt, 2014; Kraehenmann, 2017). Other candidates include the ‘dreamy-state’ aura of temporal lobe epilepsy (Carhart-Harris, 2007), meditative states (Millière, 2017) and certain states observed in dissociative identity disorder (Laddis et al., 2017).

Our emphasis on the importance of contextual factors may seem neglectful of possible genetic and neurodevelopmental predispositions to psychosis and the heterogeneous aetiologies of psychotic disorders more generally. We do accept that not all psychoses have their origins in easily identifiable PiMSs and that genetic and neurodevelopmental factors can influence both the occurrence and outcome of a PiMS. Thus, contrary to dismissing remote aetiological factors, it is our hope that this review will increase interest in translational research and a biopsychosocial perspective that appreciates the relevance of conditional and relational dependencies.

Our selective focus on the 5-HT2AR might also be critiqued but we feel this is justified by the wealth of supportive evidence for its association with PiMS-relevant phenomena and that this may have been overlooked in the past. The present upsurge in interest in classic psychedelics should be welcomed as it has historically been unreasonably difficult to conduct research with these compounds (Nutt et al., 2013). It is inevitable that progress in our understanding of the 5-HT2AR system will have suffered as a result. We acknowledge that other neurotransmitters such as noradrenaline and acetylcholine (Angela and Dayan, 2005), glutamate (Corlett et al., 2016), dopamine (Howes and Kapur, 2009) as well as the endocannabinoid (Ibarra-Lecue et al., 2018) and opiate systems (Butelman and Kreek, 2015) will likely play a role in shaping the quality of PiMSs and their outcome but we maintain that the 5-HT2AR plays a particularly central role, deserving of special attention.

We might also consider whether PiMSs are always transient states or whether they can occur as more protracted episodes or phases, perhaps with more attenuated characteristics in each of the three defining criteria. Infancy, and childhood more generally, might be considered a protracted PiMS or pivotal mental *phase*. Indeed, the quality of consciousness in infancy has been likened to psychedelic (Carhart-Harris et al., 2018b; Carhart-Harris and Nutt, 2014) and spiritual states (Woodsworth, 1884) and is a period during which we are particularly sensitive to contextual influences that may have an exaggerated influence on our psychological development (Gopnik et al., 1999; Rapoport and Gogtay, 2008; Weimer et al., 2013). Perhaps a period of ‘microdosing’ with a psychedelic might be considered another valid example of a pivotal mental *phase* rather than a singular state per se (Kuypers et al., 2019) and, similarly, the at-risk mental state may express more as a protracted phase or process, rather than an entirely discrete state.

The PiMS model could also be applied to processes of ideological radicalization and deradicalization. According to one constructivist model of radicalization, self-invalidation and uncertainty lead to a creative reconstruing of the self, followed by a tightening of beliefs around a newfound identity (Feixas and Winter, 2019). This theory is consistent with the ‘Decentring’ model of religious experience (McNamara, 2009), the social defeat hypothesis of schizophrenia (Selten et al., 2016) and our proposal that psychological flexibility often gives way to psychological rigidity (e.g. delusional ideation) in the psychotic process, as in many psychopathologies. Drastic recalibrations of beliefs in response to interpersonal stresses and uncertainties are common features of psychotic, religious and radical conversion experiences.

Throughout this review, we have placed emphasis on the role of stress in increasing 5-HT2AR expression and signaling and cited evidence linking increased 5-HT2AR signaling with aspects of neuroplasticity. However, there is also evidence that stress can negatively impact neuronal integrity, particularly in the hippocampus (Conrad et al., 2017) and may also cause certain functional impairments (McEwen, 1999). This apparent inconsistency needs to be addressed, if not reconciled. Attempting this, one might propose that plasticity is not a singular homogenous phenomenon (Gray et al., 2013) and (like the PiMS itself) neither is it intrinsically salutogenic, that is, promoting of health (Doidge, 2007). Moreover, there is good evidence that stress can facilitate aspects of learning (Joëls et al., 2006) as is the case with trauma, for example (Deppermann et al., 2014; Joëls et al., 2006). Thus, it would be hasty to cite evidence of the negative impact of chronic stress on regionally specific neuronal integrity (e.g. McEwen, 1999) or aberrant synaptic plasticity in schizophrenia (Stephan et al., 2009) as evidence against the model being presented here. One should also note that we have specifically identified *cortical* plasticity as a key criterion for PiMSs.

One other point of critique is that our central construct, the PiMS, merely repackages previously introduced psychological constructs such as ‘quantum change’ (C’De Baca and Wilbourne, 2004; Miller and C’de Baca, 2001) and ‘transformative experiences’ (Paul, 2014). There is, however, surprisingly little scientific literature on the latter phenomenon and both constructs lay emphasis on *change* or *outcome* rather than the *mediating state* itself. Moreover, there is little-to-no discussion of the underlying neurobiology of quantum change and an arguably one-sided emphasis on positive outcomes (Miller and C’de Baca, 2001). Thus, we feel there is a great deal of scientific and pragmatic value in focusing more on the psychological and neurobiological nature and function of an outcome *agnostic*, yet (outcome) *pivotal*, mediating *state*. We do, however, encourage the reading of literature on quantum change in particular, as it provides a rich coverage of PiMS-relevant phenomenology, including the prevalence with which individuals report psychological crises prior to the onset of a PiMS/quantum change experience (Miller and C’de Baca, 2001). Some other potentially relevant constructs include: (a) the ‘healing crisis’ or Jarisch-Herxheimer reaction (Bryceson, 1976), namely, the principle that a period of heightened pain/suffering can be part of a healing process; (b) the ‘hero’s journey’, which is the notion that a process of overcoming existential struggle and suffering can be positively (trans)formative (Campbell, 2008); and (c) the notions of allostasis (McEwen, 2019; Sterling and Eyer, 1988) and adaptive plasticity and calibration (Ellis and Del Giudice, 2019).

Finally, we are mindful that a significant amount of validation work must now be done to better define the PiMSs construct as well its usefulness as a model. This process should include demonstrating what the PiMS is not – discriminate validity. Relatedly, although good evidence has been provided that the psychedelic state meets all three defining properties of a PiMS, the same evidence has not been collated for other candidate PiMSs. This must be addressed if the case for the fundamental nature of PiMSs is to be strengthened. One way we could model the natural occurrence of PiMS in humans would be to use stress-induction paradigms that are proven to reliably upregulate the 5-HT2AR system in non-human animals, such as sleep deprivation (Elmenhorst et al., 2012; Maple et al., 2015; Zhao et al., 2019) or CO_2_ inhalation-induced hypercapnia (Buchanan et al., 2015). A 5-HT2AR antagonist pre-treatment or combined EEG and positron emission tomography (PET)-fMRI technology could be utilized to examine the neurochemistry, neurophysiology and phenomenology of the relevant state. Thus, this review should be regarded as a first step in what may be a lengthy but ultimately fruitful validation process.

## Conclusion

This paper has proposed that certain traumatic, spiritual, psychedelic and psychosis-relevant experiences can be viewed as examples of what we call PiMSs: transient, intense hyper-plastic states of mind and brain that have the potential to mediate rapid, major and potentially enduring psychological change. Various stressors have been found to upregulate the 5-HT2AR system and converging evidence implies that increased 5-HT2AR signaling may be a key molecular gateway through which PiMSs arise. The pharmacology, systems-level neurobiology and phenomenology of the (5-HT2AR agonist) psychedelic drug state is treated as particularly informative in this regard. PiMSs represent special opportunities for psychological transformation and we propose this may occur through the recalibration of mental schemata, consistent with a recent model of the therapeutic action of psychedelics (Carhart-Harris and Friston, 2019). We propose that 5-HT2AR-mediated PiMSs have evolved to aid major perspective shifts, when conditions demand them. The 5-HT2AR-mediated PiMS may represent a ‘last gasp’ attempt to resolve an intolerable situation, for example, after the expiration of mere tolerance (Carhart-Harris and Nutt, 2017). Suicide and psychosis are tragic but not inconsistent ‘solutions’ within this framework but positive, psychological ‘re-birth’ is another.

Before the relatively recent advent of secular psychology, religious traditions provided useful resources to prepare and guide individuals through PiMSs, for example, for purposes of self-transformation and growth. Note, in this regard, religions have excellent narrative and infrastructural resources for dealing with ‘psychological rebirth’ (Rambo, 1993). In modern secular environments, we presently lack the same quality of integrated support, shared values and unified vision. Thinking towards the future, consideration could be given to the value of creating humanistic contexts supportive of positive self-transformation. Consistent with principles of new-wave psychotherapies, including psychedelic therapy, one can envision an ideal future in which psychological crises are seen less as emergencies requiring immediate suppressive intervention and more as opportunities for development and growth, *if*, of course, appropriately supportive contexts can be provided for this.

## References

[bibr1-0269881120959637] Abasolo S Simons R Morgado da Silva G, et al. (2015) Lempel-Ziv complexity of cortical activity during sleep and waking in rats. J Neurophysiol 113: 2742–2752. 25717159 10.1152/jn.00575.2014 PMC4416627

[bibr2-0269881120959637] Abbott FV Hong Y Blier P, et al. (1996) Activation of 5-HT2A receptors potentiates pain produced by inflammatory mediators. Neuropharmacology 35: 99–110. 8684602 10.1016/0028-3908(95)00136-0

[bibr3-0269881120959637] Abdolmaleky HM Yaqubi S Papageorgis P, et al. (2011) Epigenetic dysregulation of HTR2A in the brain of patients with schizophrenia and bipolar disorder. Schizophr Res 129: 183–190.21550210

[bibr4-0269881120959637] Adell A Casanovas JM Artigas F (1997) Comparative study in the rat of the actions of different types of stress on the release of 5-HT in raphe nuclei and forebrain areas. Neuropharmacology 36: 735–741. 9225300 10.1016/s0028-3908(97)00048-8

[bibr5-0269881120959637] Aghajanian GK Marek GJ (2000) Serotonin model of schizophrenia: Emerging role of glutamate mechanisms. Brain Res Rev 31: 302–312. 10719157 10.1016/s0165-0173(99)00046-6

[bibr6-0269881120959637] Ahmadi M Moradi AR Esmaeili AT, et al. (2019) A preliminary study investigating time perception in adolescents with posttraumatic stress disorder and major depressive disorder. Psychol Trauma: US 11: 671–676. 10.1037/tra0000471 31135172

[bibr7-0269881120959637] Alamia A Timmermann C VanRullen R, et al. (2020) DMT alters cortical travelling waves. Available at: 10.1101/2020.05.06.080937 PMC7577737 33043883

[bibr8-0269881120959637] Alfaro-Rodríguez A González-Piña R González-Maciel, et al. (2006) Serotonin and 5-hydroxy-indole-acetic acid contents in dorsal raphe and suprachiasmatic nuclei in normal, malnourished and rehabilitated rats under 24 h of sleep deprivation. Brain Res 1110: 95–101. 16876773 10.1016/j.brainres.2006.06.069

[bibr9-0269881120959637] Aloyo VJ Dave KD (2007) Behavioral response to emotional stress in rabbits: Role of serotonin and serotonin2A receptors. Behav Pharmacol 18: 651–659. 17912049 10.1097/FBP.0b013e3282effc0d

[bibr10-0269881120959637] Alper RH (1990) Evidence for central and peripheral serotonergic control of corticosterone secretion in the conscious rat. Neuroendocrinol 51: 255–260. 10.1159/000125347 2109269

[bibr11-0269881120959637] AmatJBarattaMVPaulE, et al. (2005) Medial prefrontal cortex determines how stressor controllability affects behavior and dorsal raphe nucleus. Nat Neurosci 8: 365–371.1569616310.1038/nn1399

[bibr12-0269881120959637] Amat J Matus-Amat P Watkins LR (1998) Escapable and inescapable stress differentially and selectively alter extracellular levels of 5-HT in the ventral hippocampus and dorsal periaqueductal gray of the rat. Brain Res 797: 12–22. 9630480 10.1016/s0006-8993(98)00368-0

[bibr13-0269881120959637] Amidfar M Kim YK Colic L, et al. (2017) Increased levels of 5HT2A receptor mRNA expression in peripheral blood mononuclear cells of patients with major depression: Correlations with severity and duration of illness. Nord J Psychiat 71: 282–288. 10.1080/08039488.2016.1276624 28125323

[bibr14-0269881120959637] Anderson CL Monroy M Keltner D (2018) Awe in nature heals: Evidence from military veterans, at-risk youth, and college students. Emotion 18: 1195–1202. 29927260 10.1037/emo0000442

[bibr15-0269881120959637] Andrade R (2011) Serotonergic regulation of neuronal excitability in the prefrontal cortex. Neuropharmacology 61: 382–386. 21251917 10.1016/j.neuropharm.2011.01.015 PMC3110517

[bibr16-0269881120959637] Angela JY Dayan P (2005) Uncertainty, neuromodulation, and attention. Neuron 46: 681–692. 15944135 10.1016/j.neuron.2005.04.026

[bibr17-0269881120959637] Anisman H Du L Palkovits M, et al. (2008) Serotonin receptor subtype and p11 mRNA expression in stress-relevant brain regions of suicide and control subjects. J Psychiatr Neurosci 33: 131–141. PMC2265306 18330459

[bibr18-0269881120959637] Anju TR Smijin S Korah PK, et al. (2011) Cortical 5HT2A receptor function under hypoxia in neonatal rats: Role of glucose, oxygen, and epinephrine resuscitation. J Mol Neurosci 43: 350–357. 20857344 10.1007/s12031-010-9449-3

[bibr19-0269881120959637] Araneda R Andrade R (1991) 5-Hydroxytryptamine2 and 5-hydroxytryptamine 1A receptors mediate opposing responses on membrane excitability in rat association cortex. Neurosci 40: 399–412. 10.1016/0306-4522(91)90128-b 1851255

[bibr20-0269881120959637] Argento E Strathdee SA Tupper K, et al. (2017) Does psychedelic drug use reduce risk of suicidality? Evidence from a longitudinal community-based cohort of marginalised women in a Canadian setting. BMJ Open 7: e016025. 10.1136/bmjopen-2017-016025 PMC5623475 28939573

[bibr21-0269881120959637] Aringhieri S Carli M Kolachalam S, et al. (2018) Molecular targets of atypical antipsychotics: From mechanism of action to clinical differences. Pharm Ther 192: 20–41. 10.1016/j.pharmthera.2018.06.012 29953902

[bibr22-0269881120959637] Audenaert K Van Laere K Dumont F, et al. (2003) Decreased 5-HT2a receptor binding in patients with anorexia nervosa. J Nucl Med 44: 163–169. 12571204

[bibr23-0269881120959637] Aydin N Fischer P Frey D (2010) Turning to God in the face of ostracism: Effects of social exclusion on religiousness. Pers Soc Psychol B 36: 742–753. 10.1177/0146167210367491 20410483

[bibr24-0269881120959637] Azmitia EC (2001) Modern views on an ancient chemical: Serotonin effects on cell proliferation, maturation, and apoptosis. Brain Res Bull 56: 413–424. 11750787 10.1016/s0361-9230(01)00614-1

[bibr25-0269881120959637] Baeken C Bossuyt A De Raedt R (2014) Dorsal prefrontal cortical serotonin 2A receptor binding indices are differentially related to individual scores on harm avoidance. Psychiat Res: Neuroim 221: 162–168. 10.1016/j.pscychresns.2013.12.005 24412555

[bibr26-0269881120959637] Bailer UF Frank GK Henry SE, et al. (2007) Exaggerated 5-HT1A but normal 5-HT2A receptor activity in individuals ill with anorexia nervosa. Biol Psychiatry 61(9): 1090–1099. 17241616 10.1016/j.biopsych.2006.07.018

[bibr27-0269881120959637] Bailer UF Price JC Meltzer CC, et al. (2004) Altered 5-HT 2A receptor binding after recovery from bulimia-type anorexia nervosa: Relationships to harm avoidance and drive for thinness. Neuropsychoph 29: 1143–1155. 10.1038/sj.npp.1300430 PMC4301578 15054474

[bibr28-0269881120959637] Baldacchino JP (2016) Visions or hallucinations? Lacan on mysticism and psychosis reconsidered: The case of St George of Malta. Brit J Psychoth 32: 392–414.

[bibr29-0269881120959637] BallardCYouakimJMCoateB, et al. (2019) Pimavanserin in Alzheimer’s disease psychosis: Efficacy in patients with more pronounced psychotic symptoms. J Prev Alzheimers Dis 6: 27–33.3056908310.14283/jpad.2018.30

[bibr30-0269881120959637] Barrett FS Bradstreet MP Leoutsakos JMS, et al. (2016) The Challenging Experience Questionnaire: Characterization of challenging experiences with psilocybin mushrooms. J Psychopharmacol 30: 1279–1295. 27856683 10.1177/0269881116678781 PMC5549781

[bibr31-0269881120959637] Barrett FS Doss MK Sepeda ND, et al. (2020) Emotions and brain function are altered up to one month after a single high dose of psilocybin. Sci Rep 10: 1–14. 32042038 10.1038/s41598-020-59282-y PMC7010702

[bibr32-0269881120959637] Barrett FS Griffiths RR (2017) Classic hallucinogens and mystical experiences: Phenomenology and neural correlates. In: Halberstadt A Vollenweider FX Nichols DE (eds) Behavioral Neurobiology of Psychedelic Drugs. Heidelberg: Springer, pp. 393–430. 10.1007/7854_2017_474 PMC6707356 28401522

[bibr33-0269881120959637] Barrett FS Johnson MW Griffiths RR (2017) Neuroticism is associated with challenging experiences with psilocybin mushrooms. Pers Indiv Differ 117: 155–160. 10.1016/j.paid.2017.06.004 PMC5540159 28781400

[bibr34-0269881120959637] Bartz JA Tchalova K Fenerci C (2016) Reminders of social connection can attenuate anthropomorphism: A replication and extension of Epley, Akalis, Waytz, and Cacioppo (2008). Psychol Sci 27: 1644–1650. 27777375 10.1177/0956797616668510

[bibr35-0269881120959637] Bastani A Rajabi S Kianimarkani F (2017) The effects of fasting during Ramadan on the concentration of serotonin, dopamine, brain-derived neurotrophic factor and nerve growth factor. Neurol Internat 9: 7043. 10.4081/ni.2017.7043 PMC5505095 28713531

[bibr36-0269881120959637] Beig MI Baumert M Walker FR, et al. (2009) Blockade of 5-HT2A receptors suppresses hyperthermic but not cardiovascular responses to psychosocial stress in rats. Neurosci 159: 1185–1191. 10.1016/j.neuroscience.2009.01.038 19356699

[bibr37-0269881120959637] Beitia G Garmendia L andAzpiroz A (2005) Time-dependent behavioral, neurochemical, and immune consequences of repeated experiences of social defeat stress in male mice and the ameliorative effects of fluoxetine. Brain Behav Immun 19: 530–539. 16214024 10.1016/j.bbi.2004.11.002

[bibr38-0269881120959637] Beliveau V Ganz M Feng L, et al. (2017) A high-resolution in vivo atlas of the human brain’s serotonin system. J Neurosci 37: 120–128. 28053035 10.1523/JNEUROSCI.2830-16.2016 PMC5214625

[bibr39-0269881120959637] Belser AB Agin-Liebes G Swift TC, et al. (2017) Patient experiences of psilocybin-assisted psychotherapy: An interpretative phenomenological analysis. J Humanist Psychol 57: 354–388.

[bibr40-0269881120959637] Belsky J Pluess M (2009) Beyond diathesis stress: Differential susceptibility to environmental influences. Psychol Bull 135: 885–908. 19883141 10.1037/a0017376

[bibr41-0269881120959637] Benekareddy M Goodfellow NM Lambe EK, et al. (2010) Enhanced function of prefrontal serotonin 5-HT2 receptors in a rat model of psychiatric vulnerability. J Neurosci 30: 12138–12150. 20826676 10.1523/JNEUROSCI.3245-10.2010 PMC4177096

[bibr42-0269881120959637] Benekareddy M Nair AR Dias BG, et al. (2012) Induction of the plasticity-associated immediate early gene Arc by stress and hallucinogens: Role of brain-derived neurotrophic factor. Int J Neuropsychop 16: 405–415. 10.1017/S1461145712000168 22404904

[bibr43-0269881120959637] Benekareddy M Vadodaria KC Nair AR, et al. (2011) Postnatal serotonin type 2 receptor blockade prevents the emergence of anxiety behavior, dysregulated stress-induced immediate early gene responses, and specific transcriptional changes that arise following early life stress. Biol Psychiatry 70: 1024–1032. 21959103 10.1016/j.biopsych.2011.08.005 PMC3210326

[bibr44-0269881120959637] Bercel NA Travis LE Olinger LB, et al. (1956) Model psychoses induced by LSD-25 in normals: II. Rorschach test findings. AMA Arch Neurol Psy 75: 612–618. 10.1001/archneurpsyc.1956.02330240050004 13325990

[bibr45-0269881120959637] Berton O Aguerre S Sarrieau A, et al. (1997) Differential effects of social stress on central serotonergic activity and emotional reactivity in Lewis and spontaneously hypertensive rats. Neurosci 82: 147–159. 10.1016/s0306-4522(97)00282-0 9483511

[bibr46-0269881120959637] Bhagwagar Z Hinz R Taylor M, et al. (2006) Increased 5-HT 2A receptor binding in euthymic, medication-free patients recovered from depression: A positron emission study with [11 C] MDL 100,907. Am J Psychiatry 163: 1580–1587. 16946184 10.1176/ajp.2006.163.9.1580

[bibr47-0269881120959637] Bibancos T Jardim DL Aneas I, et al. (2007) Social isolation and expression of serotonergic neurotransmission-related genes in several brain areas of male mice. Genes Brain Behav 6: 529–539. 17083332 10.1111/j.1601-183X.2006.00280.x

[bibr48-0269881120959637] Billac G Nichols C (2019) Elucidating anti-inflammatory signaling paradigm at the 5-HT2A receptor. FASEB J 33(1 Suppl): 503–512.

[bibr49-0269881120959637] Bland ST Hargrave D Pepin JL, et al. (2003) Stressor controllability modulates stress-induced dopamine and serotonin efflux and morphine-induced serotonin efflux in the medial prefrontal cortex. Neuropsychoph 28: 1589–1596. 10.1038/sj.npp.1300206 12784102

[bibr50-0269881120959637] Blessing WW Seaman B (2003) 5-Hydroxytryptamine(2A) receptors regulate sympathetic nerves constricting the cutaneous vascular bed in rabbits and rats. Neurosci 117: 939–948. 10.1016/s0306-4522(02)00810-2 12654345

[bibr51-0269881120959637] Bloche MG (2016) Toward a science of torture. Tex L Rev 95: 1329.

[bibr52-0269881120959637] Bogenschutz MP Forcehimes A Pommy JA, et al. (2015) Psilocybin-assisted treatment for alcohol dependence: A proof-of-concept study. J Psychopharmacol 29: 289–299. 25586396 10.1177/0269881114565144

[bibr53-0269881120959637] Bogenschutz MP Johnson MW (2016) Classic hallucinogens in the treatment of addictions. Prog Neuro-Psychoph 64: 250–258. 10.1016/j.pnpbp.2015.03.002 25784600

[bibr54-0269881120959637] Boswell JF Thompson-Hollands J Farchione TJ, et al. (2013) Intolerance of uncertainty: A common factor in the treatment of emotional disorders. J Clin Psychol 69: 630–645. 23381685 10.1002/jclp.21965 PMC3712497

[bibr55-0269881120959637] Boulougouris V Glennon JC Robbins TW (2008) Dissociable effects of selective 5-HT 2A and 5-HT 2C receptor antagonists on serial spatial reversal learning in rats. Neuropsychoph 33: 2007–2019. 10.1038/sj.npp.1301584 17957219

[bibr56-0269881120959637] Bowdle AT Radant AD Cowley DS, et al. (1998) Psychedelic effects of ketamine in healthy volunteers relationship to steady-state plasma concentrations. Anesthesiology 88: 82–88. 9447860 10.1097/00000542-199801000-00015

[bibr57-0269881120959637] Bowers MB Freedman DX (1966) Psychedelic experiences in acute psychoses. Arch Gen Psychiat 15: 240–248. 5911238 10.1001/archpsyc.1966.01730150016003

[bibr58-0269881120959637] Briere J Godbout N Dias C (2015) Cumulative trauma, hyperarousal, and suicidality in the general population: A path analysis. J Trauma Dissociatio 16: 153–169. 10.1080/15299732.2014.970265 25587939

[bibr59-0269881120959637] Brodersen E Glock M (eds) (2016) Jungian Perspectives on Rebirth and Renewal: Phoenix Rising. Abingdon-on-Thames: Taylor & Francis.

[bibr60-0269881120959637] Broome MR Johns LC Valli I, et al. (2007) Delusion formation and reasoning biases in those at clinical high risk for psychosis. Brit J Psychiat 51: s38–s42. 10.1192/bjp.191.51.s38 18055936

[bibr61-0269881120959637] Brotto LA Gorzalka BB Hanson LA (1998) Effects of housing conditions and 5-HT2A activation on male rat sexual behavior. Physiol Behav 63: 475–479. 9523886 10.1016/s0031-9384(97)00482-4

[bibr62-0269881120959637] Brown GL Linnoila MI (1990) CSF serotonin metabolite (5-HIAA) studies in depression, impulsivity, and violence. J Clin Psychiat 51(Suppl): 31–41. 1691169

[bibr63-0269881120959637] Brown RP Gerbarg PL (2005) Sudarshan Kriya Yogic breathing in the treatment of stress, anxiety, and depression: Part II—clinical applications and guidelines. J Altern Complem Med 11: 711–717. 10.1089/acm.2005.11.711 16131297

[bibr64-0269881120959637] Bryceson AD (1976) Clinical pathology of the Jarisch-Herxheimer reaction. J Infect Dis 133: 696–704. 932495 10.1093/infdis/133.6.696

[bibr65-0269881120959637] Bubenik GA Ball RO Pang SF (1992) The effect of food deprivation on brain and gastrointestinal tissue levels of tryptophan, serotonin, 5-hydroxyindoleacetic acid, and melatonin. J Pineal Res 12: 7–16. 1373446 10.1111/j.1600-079x.1992.tb00020.x

[bibr66-0269881120959637] Buchanan GF Smith HR MacAskill A, et al. (2015) 5-HT2A receptor activation is necessary for CO2-induced arousal. J Neurophysiol 114: 233–243. 25925320 10.1152/jn.00213.2015 PMC4507958

[bibr67-0269881120959637] Buckley P (1981) Mystical experience and schizophrenia. Schizophrenia Bull 7: 516–521. 10.1093/schbul/7.3.516 7280578

[bibr68-0269881120959637] Bull EJ Hutson PH Fone KC (2004) Decreased social behaviour following 3, 4-methylenedioxymethamphetamine (MDMA) is accompanied by changes in 5-HT2A receptor responsivity. Neuropharmacology 46: 202–210. 14680758 10.1016/j.neuropharm.2003.08.004

[bibr69-0269881120959637] Butelman ER Kreek MJ (2015) Salvinorin A, a kappa-opioid receptor agonist hallucinogen: Pharmacology and potential template for novel pharmacotherapeutic agents in neuropsychiatric disorders. Front Pharmacol 6: 190. 26441647 10.3389/fphar.2015.00190 PMC4561799

[bibr70-0269881120959637] C’De Baca J Wilbourne P (2004) Quantum change: Ten years later. J Clin Psychol 60: 531–541. 15048699 10.1002/jclp.20006

[bibr71-0269881120959637] Cahir M Ardis T Reynolds GP, et al. (2007) Acute and chronic tryptophan depletion differentially regulate central 5-HT 1A and 5-HT 2A receptor binding in the rat. Psychopharmacology 190: 497–506. 17124620 10.1007/s00213-006-0635-5

[bibr72-0269881120959637] Calogero AE Bernardini R Margioris AN, et al. (1989) Effects of serotonergic agonists and antagonists on corticotropin-releasing hormone secretion by explanted rat hypothalami. Peptides 10: 189–200. 2787501 10.1016/0196-9781(89)90096-x

[bibr73-0269881120959637] Cameron LP Benson CJ Dunlap LE, et al. (2018) Effects of N, N-dimethyltryptamine on rat behaviors relevant to anxiety and depression. ACS Chem Neurosci 9: 1582–1590. 29664276 10.1021/acschemneuro.8b00134 PMC7196340

[bibr74-0269881120959637] Campbell J (2008) The Hero with a Thousand Faces. Novato, CA: New World Library.

[bibr75-0269881120959637] Camporesi P (1988) The Incorruptible Flesh: Bodily Mutation and Mortification in Religion and Folklore. Cambridge: Cambridge University Press.

[bibr76-0269881120959637] Cangas AJ Sass LA Pérez-Álvarez M (2008) From the visions of Saint Teresa of Jesus to the voices of schizophrenia. Philo Psychia Psychol 15: 239–250.

[bibr77-0269881120959637] Cant R Cooper S Chung C, et al. (2012) The divided self: Near death experiences of resuscitated patients–A review of literature. Int Emerg Nurs 20: 88–93. 22483004 10.1016/j.ienj.2011.05.005

[bibr78-0269881120959637] Carbonaro TM Bradstreet MP Barrett FS, et al. (2016) Survey study of challenging experiences after ingesting psilocybin mushrooms: Acute and enduring positive and negative consequences. J Psychopharmacol 30: 1268–1278. 27578767 10.1177/0269881116662634 PMC5551678

[bibr79-0269881120959637] Carbonaro TM Johnson MW Griffiths RR (2017) Comparison of anomalous experiences after ingesting psilocybin mushrooms in research and non-research settings. Drug Alcohol Depen 171: e34.

[bibr80-0269881120959637] Cardno AG Owen MJ (2014) Genetic relationships between schizophrenia, bipolar disorder, and schizoaffective disorder. Schizophrenia Bull 40: 504–515. 10.1093/schbul/sbu016 PMC3984527 24567502

[bibr81-0269881120959637] Carhart-Harris RL (2007) Waves of the unconscious: The neurophysiology of dreamlike phenomena and its implications for the psychodynamic model of the mind. Neuropsychoanalysis 9: 183–211.

[bibr82-0269881120959637] Carhart-Harris RL (2018a) Serotonin, psychedelics and psychiatry. World Psychiatry 17: 358–359. 30192100 10.1002/wps.20555 PMC6127802

[bibr83-0269881120959637] Carhart-Harris RL (2018b) The entropic brain-revisited. Neuropharmacology 142: 167–178. 29548884 10.1016/j.neuropharm.2018.03.010

[bibr84-0269881120959637] Carhart-Harris RL (2019) How do psychedelics work? Curr Opin Psychiatr 32: 16–21. 10.1097/YCO.0000000000000467 30394903

[bibr85-0269881120959637] Carhart-Harris RL Bolstridge M Day CMJ, et al. (2018a) Psilocybin with psychological support for treatment-resistant depression: Six-month follow-up. Psychopharmacology 235: 399–408. 29119217 10.1007/s00213-017-4771-x PMC5813086

[bibr86-0269881120959637] Carhart-Harris RL Bolstridge M Rucker J, et al. (2016a) Psilocybin with psychological support for treatment-resistant depression: An open-label feasibility study. Lancet Psychiat 3: 619–627. 10.1016/S2215-0366(16)30065-7 27210031

[bibr87-0269881120959637] Carhart-Harris RL Brugger S Nutt DJ, et al. (2013) Psychiatry’s next top model: Cause for a re-think on drug models of psychosis and other psychiatric disorders. J Psychopharmacol 27: 771–778. 23784738 10.1177/0269881113494107

[bibr88-0269881120959637] Carhart-Harris RL Erritzoe D Haijen E, et al. (2018b). Psychedelics and connectedness. Psychopharmacology 235: 547–550. 28795211 10.1007/s00213-017-4701-y

[bibr89-0269881120959637] Carhart-Harris RL Erritzoe D Williams T, et al. (2012a). Neural correlates of the psychedelic state as determined by fMRI studies with psilocybin. P Natl Acad Sci 109: 2138–2143. 10.1073/pnas.1119598109 PMC3277566 22308440

[bibr90-0269881120959637] Carhart-Harris RL Friston KJ (2010) The default-mode, ego-functions and free-energy: A neurobiological account of Freudian ideas. Brain 133: 1265–1283. 20194141 10.1093/brain/awq010 PMC2850580

[bibr91-0269881120959637] Carhart-Harris RL Friston KJ (2019) REBUS and the anarchic brain: Toward a unified model of the brain action of psychedelics. Pharmacol Rev 71: 316–344. 31221820 10.1124/pr.118.017160 PMC6588209

[bibr92-0269881120959637] Carhart-Harris RL Goodwin GM (2017) The therapeutic potential of psychedelic drugs: Past, present, and future. Neuropsychoph 42: 2105–2113. 10.1038/npp.2017.84 PMC5603818 28443617

[bibr93-0269881120959637] Carhart-Harris RL Kaelen M Bolstridge M, et al. (2016b) The paradoxical psychological effects of lysergic acid diethylamide (LSD). Psychol Med 46: 1379–1390. 26847689 10.1017/S0033291715002901

[bibr94-0269881120959637] Carhart-Harris RL Kaelen M Whalley MG, et al. (2015) LSD enhances suggestibility in healthy volunteers. Psychopharmacology 232: 785–794. 25242255 10.1007/s00213-014-3714-z

[bibr95-0269881120959637] Carhart-Harris RL Leech R Erritzoe D, et al. (2012b) Functional connectivity measures after psilocybin inform a novel hypothesis of early psychosis. Schizophrenia Bull 39: 1343–1351. 10.1093/schbul/sbs117 PMC3796071 23044373

[bibr96-0269881120959637] Carhart-Harris RL Leech R Hellyer PJ, et al. (2014a) The entropic brain: A theory of conscious states informed by neuroimaging research with psychedelic drugs. Front Hum Neurosci 8: 20. 24550805 10.3389/fnhum.2014.00020 PMC3909994

[bibr97-0269881120959637] Carhart-Harris RL Muthukumaraswamy S Roseman L, et al. (2016c) Neural correlates of the LSD experience revealed by multimodal neuroimaging. P Natl Acad Sci 113: 4853–4858. 10.1073/pnas.1518377113 PMC4855588 27071089

[bibr98-0269881120959637] Carhart-Harris RL Nutt DJ (2014) Was it a vision or a waking dream?. Front Psychol 5: 255. 24772095 10.3389/fpsyg.2014.00255 PMC3983501

[bibr99-0269881120959637] Carhart-Harris RL Nutt DJ (2017) Serotonin and brain function: A tale of two receptors. J Psychopharmacol 31: 1091–1120. 28858536 10.1177/0269881117725915 PMC5606297

[bibr100-0269881120959637] Carhart-Harris RL Roseman L Bolstridge M, et al. (2017) Psilocybin for treatment-resistant depression: fMRI-measured brain mechanisms. Sci Rep 7: 13187. 29030624 10.1038/s41598-017-13282-7 PMC5640601

[bibr101-0269881120959637] Carhart-Harris RL Roseman L Haijen E, et al. (2018c) Psychedelics and the essential importance of context. J Psychopharmacol 32: 725–731. 29446697 10.1177/0269881118754710

[bibr102-0269881120959637] Carhart-Harris RL Wall MB Erritzoe D, et al. (2014b) The effect of acutely administered MDMA on subjective and BOLD-fMRI responses to favourite and worst autobiographical memories. Int J Neuropsychoph 17: 527–540. 10.1017/S1461145713001405 24345398

[bibr103-0269881120959637] Carrigan N Barkus E (2017) Schizotypy and cognitive failures: A mediating role for affect. Psychopathology 50: 195–202. 28486227 10.1159/000464106

[bibr104-0269881120959637] Castro ECC Sen P Parks WT, et al. (2017) The role of serotonin transporter in human lung development and in neonatal lung disorders. Can Respir J 2017: 9064046. 28316463 10.1155/2017/9064046 PMC5337869

[bibr105-0269881120959637] Catlow BJ Song S Paredes DA, et al. (2013) Effects of psilocybin on hippocampal neurogenesis and extinction of trace fear conditioning. Exp Brain Res 228: 481–491. 23727882 10.1007/s00221-013-3579-0

[bibr106-0269881120959637] Cavus I Duman RS (2003) Influence of estradiol, stress, and 5-HT2A agonist treatment on brain-derived neurotrophic factor expression in female rats. Biol Psychiatry 54: 59–69. 12842309 10.1016/s0006-3223(03)00236-1

[bibr107-0269881120959637] Chan CC Spencer CC West C, et al. (2015) Metacognitive processes in psychometrically defined schizotypy. Psychiat Res 230: 279–286. 10.1016/j.psychres.2015.09.006 26381182

[bibr108-0269881120959637] Chang CC Fang WH Chang HA, et al. (2017) Serotonin 2A receptor (5-HT2A) gene promoter variant interacts with chronic perceived stress to modulate resting parasympathetic activity in humans. Psychoneuroendocrino 76: 119–126. 10.1016/j.psyneuen.2016.11.015 27912162

[bibr109-0269881120959637] Chaouloff F Baudrie V Coupry I (1994) Effects of chlorisondamine and restraint on cortical [3H] ketanserin binding, 5-HT2A receptor-mediated head shakes, and behaviours in models of anxiety. Neuropharmacology 33: 449–456. 7984283 10.1016/0028-3908(94)90075-2

[bibr110-0269881120959637] Chaouloff F Coupry I Baudrie V (1995) Cortical [3H] ketanserin binding and 5-HT2A receptor-mediated behavioral responses in obese Zucker rats. Pharmacol Biochem Be 50: 309–312. 10.1016/0091-3057(94)00297-v 7740073

[bibr111-0269881120959637] Charney DS Woods SW Goodman WK, et al. (1987) Serotonin function in anxiety. Psychopharmacology 92: 14–24. 3110824 10.1007/BF00215473

[bibr112-0269881120959637] Cheah SY Lawford BR Young RM, et al. (2017) mRNA expression and DNA methylation analysis of serotonin receptor 2A (HTR2A) in the human schizophrenic brain. Genes 8: 14. 10.3390/genes8010014 PMC5295009 28054990

[bibr113-0269881120959637] Chiu HY Chan MH Lee MY, et al. (2014) Long-lasting alterations in 5-HT 2A receptor after a binge regimen of methamphetamine in mice. Int J Neuropsychopharmacol 17(10): 647–1658. 10.1017/S1461145714000455 24763081

[bibr114-0269881120959637] Clark A (2013) Whatever next? Predictive brains, situated agents, and the future of cognitive science. Behav Brain Sci 36: 181–204. 23663408 10.1017/S0140525X12000477

[bibr115-0269881120959637] Clark DM (1986) A cognitive approach to panic. Behav Res Ther 24: 461–470. 3741311 10.1016/0005-7967(86)90011-2

[bibr116-0269881120959637] Clinard CT Bader LR Sullivan MA, et al. (2015) Activation of 5-HT2a receptors in the basolateral amygdala promotes defeat-induced anxiety and the acquisition of conditioned defeat in Syrian hamsters. Neuropharmacology 90: 102–112. 25458113 10.1016/j.neuropharm.2014.11.016 PMC4281932

[bibr117-0269881120959637] Close JB Haijen EC Watts R, et al. (2020) Psychedelics and psychological flexibility – Results of a prospective web-survey using the Acceptance in Action Questionnaire II. J Contextual Behav Sci 16: 37–44.

[bibr118-0269881120959637] Cohen JY Amoroso MW Uchida (2015) Serotonergic neurons signal reward and punishment on multiple timescales. eLife 4: e06346. 10.7554/eLife.06346 PMC4389268 25714923

[bibr119-0269881120959637] Coid JW Ullrich S Bebbington P, et al. (2016) Paranoid ideation and violence: Meta-analysis of individual subject data of 7 population surveys. Schizophrenia Bull 42: 907–915. 10.1093/schbul/sbw006 PMC4903063 26884548

[bibr120-0269881120959637] Conrad CD Ortiz JB Judd JM (2017) Chronic stress and hippocampal dendritic complexity: Methodological and functional considerations. Physiol Behav 178: 66–81. 27887995 10.1016/j.physbeh.2016.11.017

[bibr121-0269881120959637] Corcoran CM Kimhy D Parrilla-Escobar MA, et al. (2011) The relationship of social function to depressive and negative symptoms in individuals at clinical high risk for psychosis. Psychol Med 41: 251–261. 20444306 10.1017/S0033291710000802 PMC3376746

[bibr122-0269881120959637] Corlett PR Honey GD Fletcher PC (2016) Prediction error, ketamine and psychosis: An updated model. J Psychopharmacol 30: 1145–1155. 27226342 10.1177/0269881116650087 PMC5105325

[bibr123-0269881120959637] Correll CU Penzner JB Frederickson AM, et al. (2007) Differentiation in the preonset phases of schizophrenia and mood disorders: Evidence in support of a bipolar mania prodrome. Schizophrenia Bull 33: 703–714. 10.1093/schbul/sbm028 PMC2526140 17478437

[bibr124-0269881120959637] Cortes-Altamirano JL Olmos-Hernandez A Jaime HB, et al. (2018) 5-HT1, 5-HT2, 5-HT3 and 5-HT7 receptors and their role in the modulation of pain response in the central nervous system. Curr Neuropharmacol 16: 210–221. 28901281 10.2174/1570159X15666170911121027 PMC5883380

[bibr125-0269881120959637] Courteix C Dupuis A Martin P, et al. (2018) 5-HT2A receptors and pain. In: Guiard BP Di Giovanni G (eds) 5-HT2A Receptors in the Central Nervous System. Totowa: Humana Press, pp. 339–353.

[bibr126-0269881120959637] Craddock N O’Donovan MC Owen MJ (2009) Psychosis genetics: Modeling the relationship between schizophrenia, bipolar disorder, and mixed (or ‘schizoaffective’) psychoses. Schizophrenia Bull 35: 482–490. 10.1093/schbul/sbp020 PMC2669589 19329560

[bibr127-0269881120959637] Crespi B Dinsdale N Read S, et al. (2019) Spirituality, dimensional autism, and schizotypal traits: The search for meaning. PLoS One 14: e0213456. 30849096 10.1371/journal.pone.0213456 PMC6407781

[bibr128-0269881120959637] Csordas TJ Lewton E (1998) Practice, performance, and experience in ritual healing. Transcult Psychiatry 35: 435–512.

[bibr129-0269881120959637] Cui R Fan J Ge T, et al. (2018) The mechanism of acute fasting-induced antidepressant-like effects in mice. J Cell Mol Med 22: 223–229. 28782175 10.1111/jcmm.13310 PMC5742683

[bibr130-0269881120959637] Cummings J Isaacson S Mills R, et al. (2014) Pimavanserin for patients with Parkinson’s disease psychosis: A randomised, placebo-controlled phase 3 trial. Lancet 383: 533–540. 24183563 10.1016/S0140-6736(13)62106-6

[bibr131-0269881120959637] da Silva Soares R Jr Falconi-Sobrinho LL Almada RC, et al. (2019) Dorsal raphe nucleus 5-Hydroxytryptamine 2A receptors are critical for the organisation of panic attack-like defensive behaviour and unconditioned fear-induced antinociception elicited by the chemical stimulation of superior colliculus neurons. Eur Neuropsychopharm 29: 858–870. 10.1016/j.euroneuro.2019.05.007 31227263

[bibr132-0269881120959637] Davidson CA Hoffman L Spaulding WD (2016) Schizotypal personality questionnaire–brief revised (updated): An update of norms, factor structure, and item content in a large non-clinical young adult sample. Psychiat Res 238: 345–355. 10.1016/j.psychres.2016.01.053 PMC4834869 27086255

[bibr133-0269881120959637] Davis AK Barrett FS Griffiths RR (2019) Psychological flexibility mediates the relations between acute psychedelic effects and subjective decreases in depression and anxiety. J Contextual Behav Sci 15: 39–45. 32864325 10.1016/j.jcbs.2019.11.004 PMC7451132

[bibr134-0269881120959637] Davis AK Barrett FS Griffiths RR (2020) Psychological flexibility mediates the relations between acute psychedelic effects and subjective decreases in depression and anxiety. J Contextual Behav Sci 15: 39–45. 32864325 10.1016/j.jcbs.2019.11.004 PMC7451132

[bibr135-0269881120959637] Davis J Eyre H Jacka FN, et al. (2016) A review of vulnerability and risks for schizophrenia: Beyond the two hit hypothesis. Neurosci Biobehav Rev 65: 185–194. 27073049 10.1016/j.neubiorev.2016.03.017 PMC4876729

[bibr136-0269881120959637] Day TA (2005) Defining stress as a prelude to mapping its neurocircuitry: No help from allostasis. Prog Neuro-Psychoph 29: 1195–1200. 10.1016/j.pnpbp.2005.08.005 16213079

[bibr137-0269881120959637] De Paulis T (2001) M-100907 (Aventis). Curr Opin Invest Dr 2: 123. 11527004

[bibr138-0269881120959637] Deacon BJ (2013) The biomedical model of mental disorder: A critical analysis of its validity, utility, and effects on psychotherapy research. Clin Psychol Rev 33: 846–861. 23664634 10.1016/j.cpr.2012.09.007

[bibr139-0269881120959637] Deakin JW Graeff FG (1991) 5-HT and mechanisms of defence. J Psychopharmacol 5: 305–315. 22282829 10.1177/026988119100500414

[bibr140-0269881120959637] Dean JG Liu T Huff S, et al. (2019) Biosynthesis and extracellular concentrations of N, N-dimethyltryptamine (DMT) in mammalian brain. Sci Rep 9: 9333. 31249368 10.1038/s41598-019-45812-w PMC6597727

[bibr141-0269881120959637] DeHoff SL (2015) Distinguishing mystical religious experience and psychotic experience: A qualitative study interviewing Presbyterian church (USA) professionals. Pastoral Psychol 64: 21–39.

[bibr142-0269881120959637] Deppermann S Storchak H Fallgatter AJ, et al. (2014) Stress-induced neuroplasticity: (Mal) adaptation to adverse life events in patients with PTSD–A critical overview. Neurosci 283: 166–177. 10.1016/j.neuroscience.2014.08.037 25193848

[bibr143-0269881120959637] DeVylder JE Yang LH Harkavy-Friedman JM, et al. (2014) Assessing depression in youth at clinical high risk for psychosis: A comparison of three measures. Psychiat Res 215: 323–328. 10.1016/j.psychres.2013.12.002 PMC3945159 24370335

[bibr144-0269881120959637] Diamond E (2003) Holy Men and Hunger Artists: Fasting and Asceticism in Rabbinic Culture. Oxford: Oxford University Press.

[bibr145-0269881120959637] Disner SG Beevers C. Haigh EA, et al. (2011) Neural mechanisms of the cognitive model of depression. Nat Rev Neurosci 12: 467–477. 21731066 10.1038/nrn3027

[bibr146-0269881120959637] Doidge N (2007) The Brain That Changes Itself: Stories of Personal Triumph from the Frontiers of Brain Science. London: Penguin.

[bibr147-0269881120959637] Dolan D Jensen HJ Mediano PA, et al. (2018) The improvisational state of mind: A multidisciplinary study of an improvisatory approach to classical music repertoire performance. Front Psychol 9: 1341. 10.3389/fpsyg.2018.01341 PMC6167963 30319469

[bibr148-0269881120959637] Dolder PC Schmid Y Müller F, et al. (2016) LSD acutely impairs fear recognition and enhances emotional empathy and sociality. Neuropsychoph 41: 2638–2646. 10.1038/npp.2016.82 PMC5026740 27249781

[bibr149-0269881120959637] Dos Santos RG Grasa E Valle M, et al. (2012) Pharmacology of ayahuasca administered in two repeated doses. Psychoph (Berl.) 219: 1039–1053. 10.1007/s00213-011-2434-x 21842159

[bibr150-0269881120959637] Dressler WW Balieiro MC de Araújo LF, et al. (2016) Culture as a mediator of gene-environment interaction: Cultural consonance, childhood adversity, a 2A serotonin receptor polymorphism, and depression in urban Brazil. Soc Sci Med 161: 109–117. 27270123 10.1016/j.socscimed.2016.05.033

[bibr151-0269881120959637] Dugan KM (1995) Fasting for life: The place of fasting in the Christian tradition. J Am Acad Relig 63: 539–548.

[bibr152-0269881120959637] Dwivedi Y Mondal AC Payappagoudar GV, et al. (2005) Differential regulation of serotonin (5HT) 2A receptor mRNA and protein levels after single and repeated stress in rat brain: Role in learned helplessness behavior. Neuropharmacology 48: 204–214. 15695159 10.1016/j.neuropharm.2004.10.004

[bibr153-0269881120959637] Eisner BG Cohen S (1958) Psychotherapy with lysergic acid diethylamide. J Nerv Ment Dis 127: 528–539. 13621221 10.1097/00005053-195812000-00006

[bibr154-0269881120959637] Ellis BJ Del Giudice M (2019) Developmental adaptation to stress: An evolutionary perspective. Annu Rev Psychol 70: 111–139. 30125133 10.1146/annurev-psych-122216-011732

[bibr155-0269881120959637] Elmenhorst D Kroll T Matusch A, et al. (2012) Sleep deprivation increases cerebral serotonin 2A receptor binding in humans. Sleep 35: 1615–1623. 23204604 10.5665/sleep.2230 PMC3490354

[bibr156-0269881120959637] Emsley R Chiliza B Asmal L, et al. (2013) The nature of relapse in schizophrenia. BMC Psychiat 13: 50. 10.1186/1471-244X-13-50 PMC3599855 23394123

[bibr157-0269881120959637] Engel GL (1977) The need for a new medical model: A challenge for biomedicine. Science 196: 129–136. 847460 10.1126/science.847460

[bibr158-0269881120959637] Epley N Akalis S Waytz A, et al. (2008) Creating social connection through inferential reproduction: Loneliness and perceived agency in gadgets, gods, and greyhounds. Psychol Sci 19: 114–120. 18271858 10.1111/j.1467-9280.2008.02056.x

[bibr159-0269881120959637] Erritzoe D Frokjaer VG Holst KK, et al. (2011) In vivo imaging of cerebral serotonin transporter and serotonin2A receptor binding in 3, 4-methylenedioxymethamphetamine (MDMA or ‘ecstasy’) and hallucinogen users. Arch Gen Psychiat 68: 562–576. 21646575 10.1001/archgenpsychiatry.2011.56

[bibr160-0269881120959637] Erritzoe D Nutt DJ Carhart-Harris R (2017) Concerns regarding conclusions made about LSD-treatments (received 25 10 2016). Hist Psychiatr 28: 257–260. 10.1177/0957154X17692197 28198192

[bibr161-0269881120959637] Erritzoe D Rasmussen H Kristiansen KT, et al. (2008) Cortical and subcortical 5-HT2A receptor binding in neuroleptic-naive first-episode schizophrenic patients. Neuropsychoph 33: 2435–2441. 10.1038/sj.npp.1301656 18288096

[bibr162-0269881120959637] Erritzoe D Roseman L Nour M, et al. (2018) Effects of psilocybin therapy on personality structure. Acta Psychiat Scand 138: 368–378. 29923178 10.1111/acps.12904 PMC6220878

[bibr163-0269881120959637] Eskildsen S (1998) Asceticism in Early Taoist Religion. New York: SUNY Press.

[bibr164-0269881120959637] Eyssel F Reich N (2013) Loneliness makes the heart grow fonder (of robots)—on the effects of loneliness on psychological anthropomorphism. In: 2013 8th ACM/IEEE International Conference on Human-Robot Interaction (HRI), 3–6 March, Tokyo, pp. 121–122. New York: IEEE.

[bibr165-0269881120959637] Fairbanks LA Melega WP Jorgensen MJ, et al. (2001) Social impulsivity inversely associated with CSF 5-HIAA and fluoxetine exposure in vervet monkeys. Neuropsychoph 24: 370–378. 10.1016/S0893-133X(00)00211-6 11182532

[bibr166-0269881120959637] Fanibunda SE Deb S Maniyadath B, et al. (2019) Serotonin regulates mitochondrial biogenesis and function in rodent cortical neurons via the 5-HT2A receptor and SIRT1–PGC-1α axis. P Natl Acad Sci 116: 11028–11037. 10.1073/pnas.1821332116 PMC6561197 31072928

[bibr167-0269881120959637] Farré-i-Barril NM (2012) Sleep deprivation: Asceticism, religious experience and neurological quandaries. In Fuller R (ed.) Religion and the Body. Leiden: Brill, pp. 217–234.

[bibr168-0269881120959637] Faulkner P Deakin JW (2014) The role of serotonin in reward, punishment and behavioural inhibition in humans: Insights from studies with acute tryptophan depletion. Neurosci Biobehav Rev 46: 365–378. 25195164 10.1016/j.neubiorev.2014.07.024

[bibr169-0269881120959637] Feixas G Winter DA (2019) Towards a constructivist model of radicalization and deradicalization: A conceptual and methodological proposal. Front Psychol 10: 412. 30894826 10.3389/fpsyg.2019.00412 PMC6414560

[bibr170-0269881120959637] Felleman DJ Van DE (1991) Distributed hierarchical processing in the primate cerebral cortex. Cereb Cortex 1: 1–47. 1822724 10.1093/cercor/1.1.1-a

[bibr171-0269881120959637] Fernandes C McKittrick CR File SE, et al. (1997) Decreased 5-HT1A and increased 5-HT2A receptor binding after chronic corticosterone associated with a behavioural indication of depression but not anxiety. Psychoneuroendocrino 22: 477–491. 10.1016/s0306-4530(97)00052-8 9373882

[bibr172-0269881120959637] Fernández A Gómez C Hornero R, et al. (2013) Complexity and schizophrenia. Prog Neuro-Psychoph 45: 267–276. 10.1016/j.pnpbp.2012.03.015 22507763

[bibr173-0269881120959637] Ferres-Coy A Santana N Castane A (2013) Acute 5-HT(1)A autoreceptor knockdown increases antidepressant responses and serotonin release in stressful conditions. Psychopharmacology (Berl) 225: 61–74. 22820867 10.1007/s00213-012-2795-9

[bibr174-0269881120959637] Fiocco AJ Joober R Poirier J, et al. (2007) Polymorphism of the 5-HT2A receptor gene: Association with stress-related indices in healthy middle-aged adults. Front Behav Neurosci 1: 3. 18958185 10.3389/neuro.08.003.2007 PMC2525859

[bibr175-0269881120959637] Fischman LG (1983) Dreams, hallucinogenic drug states, and schizophrenia: A psychological and biological comparison. Schizophrenia Bull 9: 73–94. 10.1093/schbul/9.1.73 6133348

[bibr176-0269881120959637] Flanagan TW Nichols CD (2018) Psychedelics as anti-inflammatory agents. Int Rev Psychiatr 30: 363–375. 10.1080/09540261.2018.1481827 30102081

[bibr177-0269881120959637] Flanagan TW Sebastian MN Battaglia DM, et al. (2019a) 5-HT2 receptor activation alleviates airway inflammation and structural remodeling in a chronic mouse asthma model. Life Sci 236: 116790. 31626791 10.1016/j.lfs.2019.116790

[bibr178-0269881120959637] Flanagan TW Sebastian MN Battaglia DM, et al. (2019b). Activation of 5-HT 2 receptors reduces inflammation in vascular tissue and cholesterol levels in high-fat diet-fed apolipoprotein E knockout mice. Sci Rep 9: 1–10. 31530895 10.1038/s41598-019-49987-0 PMC6748996

[bibr179-0269881120959637] Fond G Macgregor A Leboyer M, et al. (2013) Fasting in mood disorders: Neurobiology and effectiveness. A review of the literature. Psychiat Res 209: 253–258. 10.1016/j.psychres.2012.12.018 23332541

[bibr180-0269881120959637] Fonseca MS Murakami M Mainen ZF (2015) Activation of dorsal raphe serotonergic neurons promotes waiting but is not reinforcing. Curr Biol 25: 306–315. 25601545 10.1016/j.cub.2014.12.002

[bibr181-0269881120959637] Forstmann M Sagioglou C (2017) Lifetime experience with (classic) psychedelics predicts pro-environmental behavior through an increase in nature relatedness. J Psychopharmacol 31: 975–988. 28631526 10.1177/0269881117714049

[bibr182-0269881120959637] Fosha D (2000) The Transforming Power of Affect: A Model for Accelerated Change. New York: Basic Books.

[bibr183-0269881120959637] Fraguas D Díaz-Caneja CM Ayora M, et al. (2019) Oxidative stress and inflammation in first-episode psychosis: A systematic review and meta-analysis. Schizophrenia Bull 45: 742–751. 10.1093/schbul/sby125 PMC6581144 30169868

[bibr184-0269881120959637] Frank GK Kaye WH Meltzer CC, et al. (2002) Reduced 5-HT2A receptor binding after recovery from anorexia nervosa. Biol Psychiatry 52: 896–906. 12399143 10.1016/s0006-3223(02)01378-1

[bibr185-0269881120959637] Frankel PS Cunningham KA (2002) The hallucinogen d-lysergic acid diethylamide (d-LSD) induces the immediate-early gene c-Fos in rat forebrain. Brain Res 958: 251–260. 12470860 10.1016/s0006-8993(02)03548-5

[bibr186-0269881120959637] Freeman D Startup H Dunn G, et al. (2014) Understanding jumping to conclusions in patients with persecutory delusions: Working memory and intolerance of uncertainty. Psychol Med 44: 3017–3024. 25066636 10.1017/S0033291714000592

[bibr187-0269881120959637] Friedman M Saroglou V (2010) Religiosity, psychological acculturation to the host culture, self-esteem and depressive symptoms among stigmatized and nonstigmatized religious immigrant groups in Western Europe. Basic Appl Soc Psych 32: 185–195.

[bibr188-0269881120959637] Friedman SA Hirsch SE (1971) Extreme hyperthermia after LSD ingestion JAMA 217: 1549–155. 5109756

[bibr189-0269881120959637] Friston K (2018) Am I self-conscious? (Or does self-organization entail self-consciousness?). Front Psychol 9: 579. 29740369 10.3389/fpsyg.2018.00579 PMC5928749

[bibr190-0269881120959637] Frokjaer VG Mortensen EL Nielsen FÅ, et al. (2008) Frontolimbic serotonin 2A receptor binding in healthy subjects is associated with personality risk factors for affective disorder. Biol Psychiatry 63: 569–576. 17884017 10.1016/j.biopsych.2007.07.009

[bibr191-0269881120959637] Fuenmayor LD García S (1984) The effect of fasting on 5-hydroxytryptamine metabolism in brain regions of the albino rat. Brit J Pharmacol 83: 357–362. 6207885 10.1111/j.1476-5381.1984.tb16495.x PMC1987105

[bibr192-0269881120959637] Fujino K Yoshitake T Inoue O (2002) Increased serotonin release in mice frontal cortex and hippocampus induced by acute physiological stressors. Neurosci Lett 320: 91–95. 11849771 10.1016/s0304-3940(02)00029-0

[bibr193-0269881120959637] Galynker I Ieronimo C Perez-Acquino A, et al. (1996) Panic attacks with psychotic features. J Clin Psychiat 57: 402–406. 9746448

[bibr194-0269881120959637] Garay RP Bourin M de Paillette E, et al. (2016) Potential serotonergic agents for the treatment of schizophrenia. Expert Opin Inv Drug 25: 159–170. 10.1517/13543784.2016.1121995 26576669

[bibr195-0269881120959637] Garcia-Garcia AL Meng Q Canetta S, et al. (2017) Serotonin signaling through prefrontal cortex 5-HT1A receptors during adolescence can determine baseline mood-related behaviors. Cell Rep 18(5): 1144–1156. 28147271 10.1016/j.celrep.2017.01.021 PMC5325088

[bibr196-0269881120959637] García-Montes JM Pérez-Álvarez M Balbuena CS, et al. (2006) Metacognitions in patients with hallucinations and obsessive-compulsive disorder: The superstition factor. Behav Res Ther 44: 1091–1104. 16212934 10.1016/j.brat.2005.07.008

[bibr197-0269881120959637] Gardner KL Thrivikraman KV Lightman SL, et al. (2005) Early life experience alters behavior during social defeat: Focus on serotonergic systems. Neurosci 136: 181–191. 10.1016/j.neuroscience.2005.07.042 16182451

[bibr198-0269881120959637] Garrett MT Torres-Rivera E Brubaker M, et al. (2011) Crying for a vision: The Native American sweat lodge ceremony as therapeutic intervention. J Couns Dev 89: 318–325.

[bibr199-0269881120959637] Garssen B Visser A de Jager Meezenbroek E (2016) Examining whether spirituality predicts subjective well-being: How to avoid tautology. Psychol Relig Spirit 8: 141–148.

[bibr200-0269881120959637] Gasser P Kirchner K Passie T (2015) LSD-assisted psychotherapy for anxiety associated with a life-threatening disease: A qualitative study of acute and sustained subjective effects. J Psychopharmacol 29: 57–68. 25389218 10.1177/0269881114555249

[bibr201-0269881120959637] Geiger J (2009) The Third Man Factor. New York: Hachette Books.

[bibr202-0269881120959637] Gershon MD (2013) 5-Hydroxytryptamine (serotonin) in the gastrointestinal tract. Curr Opin Endocrinol 20: 14. 10.1097/MED.0b013e32835bc703 PMC3708472 23222853

[bibr203-0269881120959637] Gewirtz JC Chen AC Terwilliger R, et al. (2002) Modulation of DOI-induced increases in cortical BDNF expression by group II mGlu receptors. Pharmacol Biochem Be 73: 317–326. 10.1016/s0091-3057(02)00844-4 12117585

[bibr204-0269881120959637] Geyer MA Krebs-Thomson K Varty GB (1999) The effects of M100907 in pharmacological and developmental animal models of prepulse inhibition deficits in schizophrenia. Neuropsychoph 21: S134–S142.

[bibr205-0269881120959637] Geyer MA Vollenweider FX (2008) Serotonin research: Contributions to understanding psychoses. Trends Pharmacol Sci 29: 445–453. 19086254 10.1016/j.tips.2008.06.006

[bibr206-0269881120959637] Ghorpade J Lackritz JR Singh G (2006) Intrinsic religious orientation among minorities in the United States: A research note. Int J Psychol Relig 16: 51–62.

[bibr207-0269881120959637] Glennon RA Titeler M McKenney JD (1984) Evidence for 5-HT_2_ receptor involvement in the mechanism of action of hallucinogenic agents. Life Sci 35: 2505–2511. 6513725 10.1016/0024-3205(84)90436-3

[bibr208-0269881120959637] Gimpl MP Gormezano I Harvey JA (1979) Effects of LSD on learning as measured by classical conditioning of the rabbit nictitating membrane response. J Pharmacol Exp Ther 208: 330–334. 762668

[bibr209-0269881120959637] Girn M Roseman L Bernhardt B, et al. (2020) LSD flattens the functional hierarchy of the human brain.

[bibr210-0269881120959637] Giustarini G (2012) The role of fear (Bhaya) in the nikāyas and in the abhidhamma. J Indian Philos 40: 511–531.

[bibr211-0269881120959637] Godar SC Mosher LJ Scheggi S, et al. (2019) Gene-environment interactions in antisocial behavior are mediated by early-life 5-HT2A receptor activation. Neuropharmacology 159: 107513. 30716416 10.1016/j.neuropharm.2019.01.028 PMC7578912

[bibr212-0269881120959637] Gonzalez-Maeso J Sealfon SC (2009) Psychedelics and schizophrenia. Trends Neurosci 32: 225–232. 19269047 10.1016/j.tins.2008.12.005

[bibr213-0269881120959637] Goodwin R Davidson L (2002) Panic attacks in psychosis. Acta Psychiat Scand 105: 14–19. 12086220 10.1034/j.1600-0447.2002._10424.x

[bibr214-0269881120959637] Gopnik A Meltzoff AN Kuhl PK (1999) The Scientist in the Crib: Minds, Brains, and How Children Learn. New York: William Morrow and Company.

[bibr215-0269881120959637] Gorwood P Lanfumey L Viltart, et al. (2018) 5-HT 2A receptors in eating disorders. In Di Giovanni G Guiard BP (eds) 5-HT2A Receptors in the Central Nervous System. Totowa: Humana Press, pp. 353–373.

[bibr216-0269881120959637] Gorzalka BB Hanson LA Brotto LA (1998) Chronic stress effects on sexual behavior in male and female rats: Mediation by 5-HT2A receptors. Pharmacol Biochem Be 61: 405–412. 10.1016/s0091-3057(98)00106-3 9802835

[bibr217-0269881120959637] Gouzoulis-Mayfrank E Heekeren K Neukirch A, et al. (2005) Psychological effects of (S)-ketamine and N, N-dimethyltryptamine (DMT): A double-blind, cross-over study in healthy volunteers. Pharmacopsychiatry 38: 301–311. 16342002 10.1055/s-2005-916185

[bibr218-0269881120959637] Gray JD Milner TA McEwen BS (2013) Dynamic plasticity: The role of glucocorticoids, brain-derived neurotrophic factor and other trophic factors. Neurosci 239: 214–227. 10.1016/j.neuroscience.2012.08.034 PMC3743657 22922121

[bibr219-0269881120959637] Griffiths RR Hurwitz ES Davis AK, et al. (2019) Survey of subjective ‘God encounter experiences’: Comparisons among naturally occurring experiences and those occasioned by the classic psychedelics psilocybin, LSD, ayahuasca, or DMT. PLoS One 14: e0214377. 31013281 10.1371/journal.pone.0214377 PMC6478303

[bibr220-0269881120959637] Griffiths RR Johnson MW Carducci MA, et al. (2016) Psilocybin produces substantial and sustained decreases in depression and anxiety in patients with life-threatening cancer: A randomized double-blind trial. J Psychopharmacol 30: 1181–1197. 27909165 10.1177/0269881116675513 PMC5367557

[bibr221-0269881120959637] Griffiths RR Johnson MW Richards WA, et al. (2011) Psilocybin occasioned mystical-type experiences: Immediate and persisting dose-related effects. Psychopharmacology 218: 649–665. 21674151 10.1007/s00213-011-2358-5 PMC3308357

[bibr222-0269881120959637] Griffiths RR Johnson MW Richards WA, et al. (2018) Psilocybin-occasioned mystical-type experience in combination with meditation and other spiritual practices produces enduring positive changes in psychological functioning and in trait measures of prosocial attitudes and behaviors. J Psychopharmacol 32: 49–69. 29020861 10.1177/0269881117731279 PMC5772431

[bibr223-0269881120959637] Griffiths RR Richards WA Johnson MW, et al. (2008) Mystical-type experiences occasioned by psilocybin mediate the attribution of personal meaning and spiritual significance 14 months later. J Psychopharmacol 22: 621–632. 18593735 10.1177/0269881108094300 PMC3050654

[bibr224-0269881120959637] Griffiths RR Richards WA McCann U, et al. (2006) Psilocybin can occasion mystical-type experiences having substantial and sustained personal meaning and spiritual significance. Psychopharmacology 187: 268–283. 16826400 10.1007/s00213-006-0457-5

[bibr225-0269881120959637] Grof S Grof C (eds) (1989) Spiritual Emergency: When Personal Transformation Becomes a Crisis. New York: TarcherPerigee.

[bibr226-0269881120959637] Grof S Grof C (2010) Holotropic Breathwork. Albany, NY: State University of New York.

[bibr227-0269881120959637] Grubaugh AL Zinzow HM Paul L, et al. (2011) Trauma exposure and posttraumatic stress disorder in adults with severe mental illness: A critical review. Clin Psychol Rev 31: 883–899. 21596012 10.1016/j.cpr.2011.04.003 PMC3148295

[bibr228-0269881120959637] Günther L Liebscher S Jähkel M, et al. (2008) Effects of chronic citalopram treatment on 5-HT1A and 5-HT2A receptors in group-and isolation-housed mice. Eur J Pharmacol 593: 49–61. 18657534 10.1016/j.ejphar.2008.07.011

[bibr229-0269881120959637] Guthrie SE Guthrie S (1995) Faces in the Clouds: A New Theory of Religion. Oxford: Oxford University Press.

[bibr230-0269881120959637] Hagihara H Catts VS Katayama Y, et al. (2018) Decreased brain pH as a shared endophenotype of psychiatric disorders. Neuropsychoph 43: 459–468. 10.1038/npp.2017.167 PMC5770757 28776581

[bibr231-0269881120959637] Haider S Haleem DJ (2000) Decreases of brain serotonin following a food restriction schedule of 4 weeks in male and female rats. Med Sci Monitor 6: 1061–1067. 11208456

[bibr232-0269881120959637] Haijen EC Kaelen M Roseman L, et al. (2018) Predicting responses to psychedelics: A prospective study. Front Pharmacol 9: 897. 30450045 10.3389/fphar.2018.00897 PMC6225734

[bibr233-0269881120959637] Hale MW Dady KF Evans AK, et al. (2011) Evidence for in vivo thermosensitivity of serotonergic neurons in the rat dorsal raphe nucleus and raphe pallidus nucleus implicated in thermoregulatory cooling. Exp Neurol 227: 264–278. 21111735 10.1016/j.expneurol.2010.11.012

[bibr234-0269881120959637] Haney C (2018) The psychological effects of solitary confinement: A systematic critique. Crime Justice 47: 365–416.

[bibr235-0269881120959637] Hanusch KU Janssen CH Billheimer D, et al. (2013) Whole-body hyperthermia for the treatment of major depression: Associations with thermoregulatory cooling. Am J Psychiatry 170: 802–804. 23820835 10.1176/appi.ajp.2013.12111395

[bibr236-0269881120959637] Hardy A (1979) The Spiritual Nature of Man: A Study of Contemporary Religious Experiences. Oxford: Clarendon Press.

[bibr237-0269881120959637] Hartogsohn I (2016) Set and setting, psychedelics and the placebo response: An extra-pharmacological perspective on psychopharmacology. J Psychopharmacol 30: 1259–1267. 27852960 10.1177/0269881116677852

[bibr238-0269881120959637] Harvey BH Naciti C Brand L, et al. (2003) Endocrine, cognitive and hippocampal/cortical 5HT1A/2A receptor changes evoked by a time-dependent sensitisation (TDS) stress model in rats. Brain Res 983: 97–107. 12914970 10.1016/s0006-8993(03)03033-6

[bibr239-0269881120959637] Harvey JA (1996) Serotonergic regulation of associative learning. Behav Brain Res 73: 47–50. 8788476 10.1016/0166-4328(96)00068-x

[bibr240-0269881120959637] Harvey JA (2003) Role of the serotonin 5-HT2A receptor in learning. Learn Memory 10: 355–362. 10.1101/lm.60803 PMC218001 14557608

[bibr241-0269881120959637] Hasler F Grimberg U Benz MA, et al. (2004) Acute psychological and physiological effects of psilocybin in healthy humans: A double-blind, placebo-controlled dose-effect study. Psychopharmacology (Berl) 172: 145–156. 14615876 10.1007/s00213-003-1640-6

[bibr242-0269881120959637] Hayashida S Oka T Tsuji S (2010) Repeated social defeat stress induces chronic hyperthermia in rats. Physiol Behav 101: 124–131. 20438740 10.1016/j.physbeh.2010.04.027

[bibr243-0269881120959637] Hayes SC (2019) A Liberated Mind: How to Pivot toward What Matters. New York: Penguin.

[bibr244-0269881120959637] Hayes SC Barnes-Holmes D Roche B (eds) (2013) Relational Frame Theory: A Post-Skinnerian Account of Human Language and Cognition. New York: Plenum Publishers. 10.1016/s0065-2407(02)80063-5 11605362

[bibr245-0269881120959637] Hefferon K Grealy M Mutrie N (2009) Post-traumatic growth and life threatening physical illness: A systematic review of the qualitative literature. Brit J Health Psych 14: 343–378. 10.1348/135910708X332936 18718109

[bibr246-0269881120959637] Hendricks PS Johnson MW Griffiths RR (2015) Psilocybin, psychological distress, and suicidality. J Psychopharmacol 29: 1041–1043. 26395582 10.1177/0269881115598338 PMC4721603

[bibr247-0269881120959637] Higuchi Y Soga T Parhar IS (2019) Social defeat stress decreases mRNA for monoamine oxidase A and increases 5-HT turnover in the brain of male nile tilapia (Oreochromis niloticus). Front Pharmacol 9: 1549. 10.3389/fphar.2018.01549 PMC6333864 30687104

[bibr248-0269881120959637] Hill SJ (2013) Confrontation with the Unconscious: Jungian Depth Psychology and Psychedelic Experience. London: Muswell Hill Press.

[bibr249-0269881120959637] Hillen MA Gutheil CM Strout TD, et al. (2017) Tolerance of uncertainty: Conceptual analysis, integrative model, and implications for healthcare. Soc Sci Med 180: 62–75. 28324792 10.1016/j.socscimed.2017.03.024

[bibr250-0269881120959637] Hinton DE Kirmayer LJ (2017) The flexibility hypothesis of healing. Cult Med Psychiat 41: 3–34. 10.1007/s11013-016-9493-8 27142641

[bibr251-0269881120959637] Hobson JA Hong CCH Friston KJ (2014) Virtual reality and consciousness inference in dreaming. Front Psychol 5: 1133. 10.3389/fpsyg.2014.01133 PMC4191565 25346710

[bibr252-0269881120959637] Hof W Rosales J (2011) Becoming the Iceman. Minneapolis, MN: Hillcrest Publishing Group.

[bibr253-0269881120959637] Honings S Drukker M. ten Have M, et al. (2016) Psychotic experiences and risk of violence perpetration and arrest in the general population: A prospective study. PLoS One 11: e0159023. 27447190 10.1371/journal.pone.0159023 PMC4957763

[bibr254-0269881120959637] Howes OD Kapur S (2009) The dopamine hypothesis of schizophrenia: Version III—the final common pathway. Schizophrenia Bull 35: 549–562. 10.1093/schbul/sbp006 PMC2669582 19325164

[bibr255-0269881120959637] Hoyer D (2019) Serotonin receptors nomenclature. In: Tricklebank MD and Daly E (eds) The Serotonin System. Cambridge, MA: Academic Press, pp. 63–93.

[bibr256-0269881120959637] Hüfner K Brugger H Kuster E, et al. (2018) Isolated psychosis during exposure to very high and extreme altitude–characterisation of a new medical entity. Psychol Med 48: 1872–1879. 29202898 10.1017/S0033291717003397 PMC6088769

[bibr257-0269881120959637] Hunt HT (2000) Experiences of radical personal transformation in mysticism, religious conversion, and psychosis: A review of the varieties, processes, and consequences of the numinous. J Mind Behav 21: 353–397.

[bibr258-0269881120959637] Hunt HT (2007) ‘Dark nights of the soul’: Phenomenology and neurocognition of spiritual suffering in mysticism and psychosis. Rev Gen Psychol 11: 209–234.

[bibr259-0269881120959637] Ibarra-Lecue I Mollinedo-Gajate I Meana JJ, et al. (2018) Chronic cannabis promotes pro-hallucinogenic signaling of 5-HT2A receptors through Akt/mTOR pathway. Neuropsychoph 43: 2028–2035. 10.1038/s41386-018-0076-y PMC6098160 29748632

[bibr260-0269881120959637] Iigaya K Fonseca MS Murakami M, et al. (2018) An effect of serotonergic stimulation on learning rates for rewards apparent after long intertrial intervals. Nat Commun 9: 2477. 10.1038/s41467-018-04840-2 PMC6018802 29946069

[bibr261-0269881120959637] Inaba M Maruyama T Yoshimura Y, et al. (2009) Facilitation of low-frequency stimulation-induced long-term potentiation by endogenous noradrenaline and serotonin in developing rat visual cortex. Neurosci Res 64: 191–198. 19428700 10.1016/j.neures.2009.02.014

[bibr262-0269881120959637] Ishida A Nakajima W Takada G (1997) Short-term fasting alters neonatal rat striatal dopamine levels and serotonin metabolism: An in vivo microdialysis study. Dev Brain Res 104: 131–136. 9466715 10.1016/s0165-3806(97)00149-1

[bibr263-0269881120959637] Ishiwata T Greenwood BN (2018) Changes in thermoregulation and monoamine release in freely moving rats during cold exposure and inhibition of the ventromedial, dorsomedial, or posterior hypothalamus. J Comp Physiol B 188: 541–551. 29075844 10.1007/s00360-017-1130-5

[bibr264-0269881120959637] Ishiwata T Saito T Hasegawa H, et al. (2004) Changes of body temperature and extracellular serotonin level in the preoptic area and anterior hypothalamus after thermal or serotonergic pharmacological stimulation of freely moving rats. Life Sci 75: 2665–2675. 15369702 10.1016/j.lfs.2004.04.040

[bibr265-0269881120959637] Iyassu R Jolley S Bebbington P, et al. (2014) Psychological characteristics of religious delusions. Soc Psych Psych Epid 49: 1051–1061. 10.1007/s00127-013-0811-y PMC4173112 24379014

[bibr266-0269881120959637] Jackson M (1997) Benign schizotypy? The case of spiritual experience. In: Claridge G (ed.) Schizotypy: Implications for Illness and Health. Oxford: Oxford University Press, pp. 227–250.

[bibr267-0269881120959637] Jaggar M Vaidya VA (2018) 5-HT 2A receptors and BDNF regulation: Implications for psychopathology. In: BGuiard BP Di Giovanni G (eds) 5-HT2A Receptors in the Central Nervous System. Totowa: Humana Press, pp. 395–438.

[bibr268-0269881120959637] Jaggar M Weisstaub N Gingrich JA, et al. (2017) 5-HT2A receptor deficiency alters the metabolic and transcriptional, but not the behavioral, consequences of chronic unpredictable stress. Neurobiol Stress 7: 89–102. 28626787 10.1016/j.ynstr.2017.06.001 PMC5470573

[bibr269-0269881120959637] James W (2003 [1902]) The Varieties of Religious Experience: A Study in Human Nature. Abingdon-on-Thames: Routledge.

[bibr270-0269881120959637] Janssen CW Lowry CA Mehl MR, et al. (2016) Whole-body hyperthermia for the treatment of major depressive disorder: A randomized clinical trial. JAMA Psychiat 73: 789–795. 10.1001/jamapsychiatry.2016.1031 27172277

[bibr271-0269881120959637] Jauhar S Nour MM Veronese M, et al. (2017) A test of the transdiagnostic dopamine hypothesis of psychosis using positron emission tomographic imaging in bipolar affective disorder and schizophrenia. JAMA Psychiat 74: 1206–1213. 10.1001/jamapsychiatry.2017.2943 PMC6059355 29049482

[bibr272-0269881120959637] Javitt DC Steinschneider M Schroeder CE, et al. (1996) Role of cortical N-methyl-D-aspartate receptors in auditory sensory memory and mismatch negativity generation: Implications for schizophrenia. P Natl Acad Sci 93: 11962–11967. 10.1073/pnas.93.21.11962 PMC38166 8876245

[bibr273-0269881120959637] Jaynes J (1976) The Origin of Consciousness in the Breakdown of the Bicameral Mind. Boston, MA: Houghton Mifflin Harcourt.

[bibr274-0269881120959637] Jia X (2016) Translational medicine: Creating the crucial bidirectional bridge between bench and bedside. J Mol Sci 17: 1918. 10.3390/ijms17111918 PMC5133915 27854337

[bibr275-0269881120959637] Jiang Y Cui C Ge H, et al. (2016) Effect of 5-HT2A receptor polymorphisms and occupational stress on self-reported sleep quality: A cross-sectional study in Xinjiang, China. Sleep Med 20: 30–36. 27318223 10.1016/j.sleep.2015.12.007

[bibr276-0269881120959637] Jitsuiki H Kagaya A Goto S, et al. (2000) Effect of lithium carbonate on the enhancement of serotonin 2A receptor elicited by dexamethasone. Neuropsychobiology 41: 55–61. 10644925 10.1159/000026634

[bibr277-0269881120959637] Jitsuki S Takemoto K Kawasaki T, et al. (2011) Serotonin mediates cross-modal reorganization of cortical circuits. Neuron 69: 780–792. 21338886 10.1016/j.neuron.2011.01.016 PMC3503249

[bibr278-0269881120959637] Joëls M Pu Z Wiegert O, et al. (2006) Learning under stress: How does it work? Trends Cogn Sci 10: 152–158. 16513410 10.1016/j.tics.2006.02.002

[bibr279-0269881120959637] Johnson MW (2018) Psychiatry might need some psychedelic therapy. Int Rev Psychiatr 30: 285–290. 10.1080/09540261.2018.1509544 31282826

[bibr280-0269881120959637] Johnson MW Garcia-Romeu A Cosimano MP, et al. (2014) Pilot study of the 5-HT2AR agonist psilocybin in the treatment of tobacco addiction. J Psychopharmacol 28: 983–992. 25213996 10.1177/0269881114548296 PMC4286320

[bibr281-0269881120959637] Johnson MW Richards WA Griffiths RR (2008) Human hallucinogen research: Guidelines for safety. J Psychopharmacol 22: 603–620. 18593734 10.1177/0269881108093587 PMC3056407

[bibr282-0269881120959637] Johnson PL Hollis JH Moratalla R, et al. (2005) Acute hypercarbic gas exposure reveals functionally distinct subpopulations of serotonergic neurons in rats. J Psychopharmacol 19(4): 327–341. 15982987 10.1177/0269881105053281

[bibr283-0269881120959637] Jokela M Lehtimäki T Keltikangas-Järvinen L (2007) The influence of urban/rural residency on depressive symptoms is moderated by the serotonin receptor 2A gene. Am J Med Genet B 144: 918–922. 10.1002/ajmg.b.30555 17510953

[bibr284-0269881120959637] Jones KA Srivastava DP Allen JA, et al. (2009) Rapid modulation of spine morphology by the 5-HT2A serotonin receptor through kalirin-7 signaling. P Natl Acad Sci 106: 19575–19580. 10.1073/pnas.0905884106 PMC2780750 19889983

[bibr285-0269881120959637] Jones PB Bebbington P Foerster A, et al. (1993) Premorbid social underachievement in schizophrenia: Results from the Camberwell Collaborative Psychosis Study. Brit J Psychiat 162: 65–71. 10.1192/bjp.162.1.65 8425142

[bibr286-0269881120959637] Josipovic Z (2014) Neural correlates of nondual awareness in meditation. Ann NY Acad Sci 1307: 9–18. 24033505 10.1111/nyas.12261

[bibr287-0269881120959637] Joules R Doyle OM Schwarz AJ, et al. (2015) Ketamine induces a robust whole-brain connectivity pattern that can be differentially modulated by drugs of different mechanism and clinical profile. Psychopharmacology 232: 4205–4218. 25980482 10.1007/s00213-015-3951-9 PMC4600469

[bibr288-0269881120959637] Jouvet M (1999) Sleep and serotonin: An unfinished story. Neuropsychoph 21: 24S–27S. 10.1016/S0893-133X(99)00009-3 10432485

[bibr289-0269881120959637] Jungaberle H Thal S Zeuch A, et al. (2018) Positive psychology in the investigation of psychedelics and entactogens: A critical review. Neuropharmacology 142: 179–199. 29964094 10.1016/j.neuropharm.2018.06.034

[bibr290-0269881120959637] Kalucy MJ Grunstein R Lambert T, et al. (2013) Obstructive sleep apnoea and schizophrenia–A research agenda. Sleep Med Rev 17: 357–365. 23528272 10.1016/j.smrv.2012.10.003

[bibr291-0269881120959637] Kalynchuk LE Pinel JP Meaney MJ (2006) Serotonin receptor binding and mRNA expression in the hippocampus of fearful amygdala-kindled rats. Neurosci Lett 396: 38–43. 16324784 10.1016/j.neulet.2005.11.005

[bibr292-0269881120959637] Kavanagh DH Tansey KE O’Donovan MC, et al. (2015) Schizophrenia genetics: Emerging themes for a complex disorder. Mol Psychiatr 20: 72–76. 10.1038/mp.2014.148 25385368

[bibr293-0269881120959637] Kaye WH Wierenga CE Bailer UF, et al. (2013) Nothing tastes as good as skinny feels: The neurobiology of anorexia nervosa. Trends Neurosci 36: 110–120. 23333342 10.1016/j.tins.2013.01.003 PMC3880159

[bibr294-0269881120959637] Keeney A Jessop DS Harbuz MS, et al. (2006) Differential effects of acute and chronic social defeat stress on hypothalamic-pituitary-adrenal axis function and hippocampal serotonin release in mice. J Neuroendocrinol 18: 330–338. 16629831 10.1111/j.1365-2826.2006.01422.x

[bibr295-0269881120959637] Kelly KJ Donner NC Hale MW, et al. (2011) Swim stress activates serotonergic and nonserotonergic neurons in specific subdivisions of the rat dorsal raphe nucleus in a temperature-dependent manner. Neurosci 197: 251–268. 10.1016/j.neuroscience.2011.09.011 PMC3219528 21945646

[bibr296-0269881120959637] Kessler RC McLaughlin KA Green JG, et al. (2010) Childhood adversities and adult psychopathology in the WHO World Mental Health Surveys. Brit J Psychiat 197: 378–385. 10.1192/bjp.bp.110.080499 PMC2966503 21037215

[bibr297-0269881120959637] Kettner H Gandy S Haijen EC, et al. (2019) From egoism to ecoism: Psychedelics increase nature relatedness in a state-mediated and context-dependent manner. Int J Env Res Pub He 16: 5147. 10.3390/ijerph16245147 PMC6949937 31888300

[bibr298-0269881120959637] Khanna S Greyson B (2015) Near-death experiences and posttraumatic growth. J Nerv Ment Dis 203: 749–755. 26348586 10.1097/NMD.0000000000000362

[bibr299-0269881120959637] Kielhöfer H (2011) Bifurcation Theory: An Introduction with Applications to Partial Differential Equations, vol. 156. Heidelberg: Springer Science & Business Media.

[bibr300-0269881120959637] Kilcommons AM Morrison AP (2005) Relationships between trauma and psychosis: An exploration of cognitive and dissociative factors. Acta Psychiat Scand 112: 351–359. 16223422 10.1111/j.1600-0447.2005.00623.x

[bibr301-0269881120959637] Kilpatrick DG Ruggiero KJ Acierno R, et al. (2003) Violence and risk of PTSD, major depression, substance abuse/dependence, and comorbidity: Results from the National Survey of Adolescents. J Consult Clin Psych 71: 692–700. 10.1037/0022-006x.71.4.692 12924674

[bibr302-0269881120959637] Kim DD Barr AM White RF, et al. (2019) Clozapine-induced obsessive–compulsive symptoms: Mechanisms and treatment. J Psychiatr Neurosci 44: 71–72. 10.1503/jpn.180087 PMC6306284 30565908

[bibr303-0269881120959637] King AR Martin IL Melville KA (1974) Reversal learning enhanced by lysergic acid diethylamide (LSD): Concomitant rise in brain 5-hydroxytryptamine levels. Brit J Pharmacol 52: 419. 4458849 10.1111/j.1476-5381.1974.tb08611.x PMC1777004

[bibr304-0269881120959637] Kirkbride JB Jones PB Ullrich S, et al. (2012) Social deprivation, inequality, and the neighborhood-level incidence of psychotic syndromes in East London. Schizophrenia Bull 40: 169–180. 10.1093/schbul/sbs151 PMC3885290 23236081

[bibr305-0269881120959637] Klein DF (1993) False suffocation alarms, spontaneous panics, and related conditions: An integrative hypothesis. Arch Gen Psychiat 50: 306–317. 8466392 10.1001/archpsyc.1993.01820160076009

[bibr306-0269881120959637] Klemenc-Ketis Z Kersnik J Grmec S (2010) The effect of carbon dioxide on near-death experiences in out-of-hospital cardiac arrest survivors: A prospective observational study. Crit Care 14: R56. 20377847 10.1186/cc8952 PMC2887177

[bibr307-0269881120959637] Klock JC Boerne U Becker CE (1975) Coma, hyperthermia, and bleeding associated with massive LSD overdose, a report of eight cases. Clin Toxicol 8: 191–203. 1149410 10.3109/15563657508988063

[bibr308-0269881120959637] Kornfield J (2001) After the Ecstasy, the Laundry: How the Heart Grows Wise on the Spiritual Path. New York: Bantam.

[bibr309-0269881120959637] Kotler S Wheal J (2017) Stealing Fire: How Silicon Valley, the Navy SEALs, and Maverick Scientists Are Revolutionizing the Way We Live and Work. New York: Harper Collins.

[bibr310-0269881120959637] Koyanagi A Stickley A (2015) The association between psychosis and severe pain in community-dwelling adults: Findings from 44 low-and middle-income countries. J Psychiat Res 69: 19–26. 26343590 10.1016/j.jpsychires.2015.07.020

[bibr311-0269881120959637] Kraehenmann R (2017) Dreams and psychedelics: Neurophenomenological comparison and therapeutic implications. Curr Neuropharmacol 15: 1032–1042. 28625125 10.2174/1573413713666170619092629 PMC5652011

[bibr312-0269881120959637] Kraehenmann R Pokorny D Aicher H, et al. (2017) LSD increases primary process thinking via serotonin 2A receptor activation. Front Pharmacol 8: 814. 29167644 10.3389/fphar.2017.00814 PMC5682333

[bibr313-0269881120959637] Kuijpers HJ Van der Heijden FMMA Tuinier S, et al. (2007) Meditation-induced psychosis. Psychopathology 40: 461–464. 17848828 10.1159/000108125

[bibr314-0269881120959637] Kupers R Frokjaer VG Naert A, et al. (2009) A PET [18F] altanserin study of 5-HT2A receptor binding in the human brain and responses to painful heat stimulation. Neuroimage 44: 1001–1007. 19007894 10.1016/j.neuroimage.2008.10.011

[bibr315-0269881120959637] Kuroda Y Mikuni M Ogawa T (1992) Effect of ACTH, adrenalectomy and the combination treatment on the density of 5-HT 2 receptor binding sites in neocortex of rat forebrain and 5-HT 2 receptor-mediated wet-dog shake behaviors. Psychopharmacology 108: 27–32. 1384079 10.1007/BF02245281

[bibr316-0269881120959637] Kuroda Y Watanabe Y Albeck DS (1994) Effects of adrenalectomy and type I or type II glucocorticoid receptor activation on 5-HT1A and 5-HT2 receptor binding and 5-HT transporter mRNA expression in rat brain. Brain Res 648: 157–161. 7922518 10.1016/0006-8993(94)91916-x

[bibr317-0269881120959637] Kuypers KP de la Torre R Farre M, et al. (2018) MDMA-induced indifference to negative sounds is mediated by the 5-HT 2A receptor. Psychopharmacology 235: 481–490. 28735368 10.1007/s00213-017-4699-1 PMC5813067

[bibr318-0269881120959637] Kuypers KP Ng L Erritzoe D, et al. (2019) Microdosing psychedelics: More questions than answers? An overview and suggestions for future research. J Psychopharmacol 33: 1039–1057. 31303095 10.1177/0269881119857204 PMC6732823

[bibr319-0269881120959637] Laddis A Dell PF Korzekwa M (2017) Comparing the symptoms and mechanisms of ‘dissociation’ in dissociative identity disorder and borderline personality disorder. J Trauma Dissociatio 18: 139–173. 10.1080/15299732.2016.1194358 27245196

[bibr320-0269881120959637] Laidlaw J (2005) A life worth leaving: Fasting to death as telos of a Jain religious life. Econ Soc 34: 178–199.

[bibr321-0269881120959637] Laloyaux J Dessart G Van der Linden M, et al. (2016) Maladaptive emotion regulation strategies and stress sensitivity mediate the relation between adverse life events and attenuated positive psychotic symptoms. Cogn Neuropsychiatry 21: 116–129. 26829655 10.1080/13546805.2015.1137213

[bibr322-0269881120959637] Lang M Krátký J Shaver JH, et al. (2015) Effects of anxiety on spontaneous ritualized behavior. Curr Biol 25: 1892–1897. 26096971 10.1016/j.cub.2015.05.049

[bibr323-0269881120959637] Laursen TM Agerbo E Pedersen CB (2009) Bipolar disorder, schizoaffective disorder, and schizophrenia overlap: A new comorbidity index. J Clin Psychiat 70: 1432–1438. 10.4088/JCP.08m04807 19538905

[bibr324-0269881120959637] Leary T Litwin GH Metzner R (1963) Reactions to psilocybin administered in a supportive environment. J Nerv Ment Dis 137: 561–573. 14087676 10.1097/00005053-196312000-00007

[bibr325-0269881120959637] Lebe M Hasenbring MI Schmieder K, et al. (2013) Association of serotonin-1A and-2A receptor promoter polymorphisms with depressive symptoms, functional recovery, and pain in patients 6 months after lumbar disc surgery. PAIN 154: 377–384. 23318131 10.1016/j.pain.2012.11.017

[bibr326-0269881120959637] Lebedev AV Kaelen M Lövdén M, et al. (2016) LSD-induced entropic brain activity predicts subsequent personality change. Hum Brain Mapp 37: 3203–3213. 27151536 10.1002/hbm.23234 PMC6867426

[bibr327-0269881120959637] Lebedev AV Lövdén M Rosenthal G, et al. (2015) Finding the self by losing the self: Neural correlates of ego-dissolution under psilocybin. Hum Brain Mapp 36: 3137–3153. 26010878 10.1002/hbm.22833 PMC6869189

[bibr328-0269881120959637] Lee H Kwak S Paik J, et al. (2007) Association between serotonin 2A receptor gene polymorphism and posttraumatic stress disorder. Psychiat Invest 4: 104–108.

[bibr329-0269881120959637] Lee MA Shlain B (1992) Acid Dreams: The Complete Social History of LSD: The CIA, the Sixties, and Beyond. New York: Grove Press.

[bibr330-0269881120959637] Letheby C (2016) The epistemic innocence of psychedelic states. Conscious Cogn 39: 28–37. 26675408 10.1016/j.concog.2015.11.012

[bibr331-0269881120959637] Li D Mabrouk OS Liu T, et al. (2015) Asphyxia-activated corticocardiac signaling accelerates onset of cardiac arrest. P Natl Acad Sci 112: E2073–E2082. 10.1073/pnas.1423936112 PMC4413312 25848007

[bibr332-0269881120959637] Liechti ME Dolder PC Schmid Y (2017) Alterations of consciousness and mystical-type experiences after acute LSD in humans. Psychopharmacology 234: 1499–1510. 27714429 10.1007/s00213-016-4453-0 PMC5420386

[bibr333-0269881120959637] Liechti ME Saur MR Gamma A, et al. (2000) Psychological and physiological effects of MDMA (‘Ecstasy’) after pretreatment with the 5-HT 2 antagonist ketanserin in healthy humans. Neuropsychoph 23: 396–404. 10.1016/S0893-133X(00)00126-3 10989266

[bibr334-0269881120959637] Lienert GA Netter P (1996) LSD response in Eysenckian trait types identified by polypredictive CFA. Pers Indiv Differ 21: 845–850.

[bibr335-0269881120959637] Lifshitz M van Elk M Luhrmann TM (2019) Absorption and spiritual experience: A review of evidence and potential mechanisms. Conscious Cogn 73: 102760. 31228696 10.1016/j.concog.2019.05.008

[bibr336-0269881120959637] Lin H Kim JG Park SW, et al. (2018) Enhancement of 5-HT 2A receptor function and blockade of Kv1. 5 by MK801 and ketamine: Implications for PCP derivative-induced disease models. Exp Mol Med 50: 47. 10.1038/s12276-018-0073-6 PMC5938026 29700292

[bibr337-0269881120959637] Liskow B (1971) Extreme hyperthermia from LSD. JAMA 218: 1049. 5171031

[bibr338-0269881120959637] Liu YZ Wang YX Jiang CL (2017) Inflammation: The common pathway of stress-related diseases. Front Hum Neurosci 11: 316. 28676747 10.3389/fnhum.2017.00316 PMC5476783

[bibr339-0269881120959637] Lombaert N Hennes M Gilissen S, et al. (2018) 5-HTR 2A and 5-HTR 3A but not 5-HTR 1A antagonism impairs the cross-modal reactivation of deprived visual cortex in adulthood. Mol Brain 11: 1–19. 30400993 10.1186/s13041-018-0404-5 PMC6218970

[bibr340-0269881120959637] Lönnqvist JE Verkasalo M Haukka J, et al. (2009) Premorbid personality factors in schizophrenia and bipolar disorder: Results from a large cohort study of male conscripts. J Abnorm Psychol 118: 418. 19413416 10.1037/a0015127

[bibr341-0269881120959637] Lord LD Expert P Atasoy S, et al. (2019) Dynamical exploration of the repertoire of brain networks at rest is modulated by psilocybin. Neuroimage 199: 127–142. 31132450 10.1016/j.neuroimage.2019.05.060

[bibr342-0269881120959637] Loyola SI (2007) The Spiritual Exercises of St. Ignatius of Loyola. New York: Cosimo, Inc.

[bibr343-0269881120959637] Luhrmann TM (2017) Diversity within the psychotic continuum. Schizophrenia Bull 43: 27–31. 10.1093/schbul/sbw137 PMC5216862 27872266

[bibr344-0269881120959637] Lukoff D (1985) The diagnosis of mystical experiences with psychotic features. J Transpersonal Psy 17: 155–181.

[bibr345-0269881120959637] Lukoff D (2007) Visionary spiritual experiences. Southern Med J 100: 635–641. 17591329 10.1097/SMJ.0b013e318060072f

[bibr346-0269881120959637] Lukoff D (2018) Spirituality and extreme states. J Humanist Psychol 59: 754–761.

[bibr347-0269881120959637] Ly C Greb AC Cameron LP, et al. (2018) Psychedelics promote structural and functional neural plasticity. Cell Rep 23: 3170–3182. 29898390 10.1016/j.celrep.2018.05.022 PMC6082376

[bibr348-0269881120959637] Lyon HM Kaney S Bentall RP (1994) The defensive function of persecutory delusions: Evidence from attribution tasks. Brit J Psychiat 164: 637–646. 10.1192/bjp.164.5.637 7921714

[bibr349-0269881120959637] Lyons T (2020) The acute and long-term effects of psilocybin in healthy volunteers, 4. Available at: https://www.youtube.com/watch?v=tSC-hu5BWTA

[bibr350-0269881120959637] Lyons T Carhart-Harris RL (2018a) Increased nature relatedness and decreased authoritarian political views after psilocybin for treatment-resistant depression. J Psychopharmacol 32: 811–819. 29338538 10.1177/0269881117748902 PMC6047302

[bibr351-0269881120959637] Lyons T Carhart-Harris RL (2018b) More realistic forecasting of future life events after psilocybin for treatment-resistant depression. Front Psychol 9: 1721. 10.3389/fpsyg.2018.01721 PMC6194345 30369890

[bibr352-0269881120959637] MacLean KA Johnson MW Griffiths RR (2011) Mystical experiences occasioned by the hallucinogen psilocybin lead to increases in the personality domain of openness. J Psychopharmacol 25: 1453–1461. 21956378 10.1177/0269881111420188 PMC3537171

[bibr353-0269881120959637] Macmillan S (2013) ‘The nyghtes watchys’: Sleep deprivation in medieval devotional culture. J Mediev Relig Cult 39: 23–42.

[bibr354-0269881120959637] Madsen MK Fisher PM Burmester D, et al. (2019) Psychedelic effects of psilocybin correlate with serotonin 2A receptor occupancy and plasma psilocin levels. Neuropsychoph 44: 1328–1334. 10.1038/s41386-019-0324-9 PMC6785028 30685771

[bibr355-0269881120959637] Madsen MK Fisher PM Stenbæk DS, et al. (2020) A single psilocybin dose is associated with long-term increased mindfulness, preceded by a proportional change in neocortical 5-HT2A receptor binding. Eur Neuropsychopharm 33: 71–80. 10.1016/j.euroneuro.2020.02.001 32146028

[bibr356-0269881120959637] Magalhaes AC Holmes KD Dale LB, et al. (2010) CRF receptor 1 regulates anxiety behavior via sensitization of 5-HT2 receptor signaling. Nat Neurosci 13: 622–629. 20383137 10.1038/nn.2529 PMC2862362

[bibr357-0269881120959637] Malone TC Mennenga SE Guss J, et al. (2018) Individual experiences in four cancer patients following psilocybin-assisted psychotherapy. Front Pharmacol 9: 256. 29666578 10.3389/fphar.2018.00256 PMC5891594

[bibr358-0269881120959637] Mann JJ Metts AV Ogden RT, et al. (2019) Quantification of 5-HT1A and 5-HT2A receptor binding in depressed suicide attempters and non-attempters. Arch Suicide Res 23: 122–133. 29281590 10.1080/13811118.2017.1417185

[bibr359-0269881120959637] Maple AM Zhao X Elizalde DI, et al. (2015) Htr2a expression responds rapidly to environmental stimuli in an Egr3-dependent manner. ACS Chem Neurosci 6: 1137–1142. 25857407 10.1021/acschemneuro.5b00031 PMC4565721

[bibr360-0269881120959637] Margulies DS Ghosh SS Goulas A (2016) Situating the default-mode network along a principal gradient of macroscale cortical organization. P Natl Acad Sci 113: 12574–12579. 10.1073/pnas.1608282113 PMC5098630 27791099

[bibr361-0269881120959637] Masdrakis VG Legaki EM Papageorgiou C, et al. (2017) Psychoticism in patients with panic disorder with or without comorbid agoraphobia. Int J Psychiat Clin 21: 181–187. 10.1080/13651501.2017.1305111 28345374

[bibr362-0269881120959637] Masters RA (2010) Spiritual Bypassing: When Spirituality Disconnects Us from What Really Matters. Berkeley, CA: North Atlantic Books.

[bibr363-0269881120959637] Masters RA Houston J (2000 [1966]) The Varieties of Psychedelic Experience: The Classic Guide to the Effects of LSD on the Human Psyche. Rochester, NY: Inner Traditions/Bear and Co.

[bibr364-0269881120959637] Matias S Lottem E Dugue GP, et al. (2017) Activity patterns of serotonin neurons underlying cognitive flexibility. eLife 6: e20552. 28322190 10.7554/eLife.20552 PMC5360447

[bibr365-0269881120959637] Mazzola-Pomietto P Aulakh CS Wozniak KM, et al. (1995) Evidence that 1-(2,5-dimethoxy-4-iodophenyl)-2-aminopropane (DOI)-induced hyperthermia in rats is mediated by stimulation of 5-HT2A receptors. Psychopharmacology 117: 193–199. 7753967 10.1007/BF02245187

[bibr366-0269881120959637] McCarthy-Jones S Longden E (2015) Auditory verbal hallucinations in schizophrenia and post-traumatic stress disorder: Common phenomenology, common cause, common interventions?. Front Psychol 6: 1071. 10.3389/fpsyg.2015.01071 PMC4517448 26283997

[bibr367-0269881120959637] McDannald MA (2015) Serotonin: Waiting but not rewarding. Curr Biol 25: R103–104. 25649815 10.1016/j.cub.2014.12.019

[bibr368-0269881120959637] McEvoy PM Mahoney AE (2011) Achieving certainty about the structure of intolerance of uncertainty in a treatment-seeking sample with anxiety and depression. J Anxiety Disord 25: 112–122. 20828984 10.1016/j.janxdis.2010.08.010

[bibr369-0269881120959637] McEwen BS (1999) Stress and hippocampal plasticity. Annu Rev Neurosci 22: 105–122. 10202533 10.1146/annurev.neuro.22.1.105

[bibr370-0269881120959637] McEwen BS (2019) The good side of ‘stress’. Stress 22: 524–525. 31237468 10.1080/10253890.2019.1631794

[bibr371-0269881120959637] McGirr A Berlim MT Bond DJ, et al. (2017) Adjunctive ketamine in electroconvulsive therapy: Updated systematic review and meta-analysis. Brit J Psychiat 210: 403–407. 10.1192/bjp.bp.116.195826 28385704

[bibr372-0269881120959637] McGurk D Cotting DI Britt TW, et al. (2006) Joining the ranks: The role of indoctrination in transforming civilians to service members. In Adler AB Castro CA Britt TW (eds) Military Life: The Psychology of Serving in Peace and Combat: Operational Stress. Santa Barbara, CA: Praeger Security International, pp. 13–31.

[bibr373-0269881120959637] McKittrick CR Blanchard DC Blanchard RJ, et al. (1995) Serotonin receptor binding in a colony model of chronic social stress. Biol Psychiatry 37: 383–393. 7772647 10.1016/0006-3223(94)00152-s

[bibr374-0269881120959637] McNamara P (2009) The Neuroscience of Religious Experience. Cambridge: Cambridge University Press.

[bibr375-0269881120959637] McNamara P (2016) Dreams and Visions: How Religious Ideas Emerge in Sleep and Dreams. Santa Barbara, CA: ABC-CLIO.

[bibr376-0269881120959637] McNamara P (2019) The Neuroscience of Sleep and Dreams. Cambridge: Cambridge University Press.

[bibr377-0269881120959637] Meduna LJ (1950) Carbondioxide Therapy: A Neurophysiological Treatment of Nervous Disorders. Springfield, IL: Charles C Thomas Publisher.

[bibr378-0269881120959637] Meller R Babity JM Grahame-Smith DG (2002) 5-HT2A receptor activation leads to increased BDNF mRNA expression in C6 glioma cells. Neuromol Med 1: 197–205. 10.1385/NMM:1:3:197 12095161

[bibr379-0269881120959637] Mellman TA Alim T Brown DD, et al. (2009) Serotonin polymorphisms and posttraumatic stress disorder in a trauma exposed African American population. Depress Anxiety 26: 993–997. 19842167 10.1002/da.20627 PMC2963151

[bibr380-0269881120959637] Meltzer HY Arvanitis L Bauer D, et al. (2004) Placebo-controlled evaluation of four novel compounds for the treatment of schizophrenia and schizoaffective disorder. Am J Psychiatry 161: 975–984. 15169685 10.1176/appi.ajp.161.6.975

[bibr381-0269881120959637] Meltzer HY Massey BW (2011) The role of serotonin receptors in the action of atypical antipsychotic drugs. Curr Opin Pharmacol 11: 59–67. 21420906 10.1016/j.coph.2011.02.007

[bibr382-0269881120959637] Meltzer HY Mills R Revell S, et al. (2010) Pimavanserin, a serotonin(2A) receptor inverse agonist, for the treatment of Parkinson’s disease psychosis. Neuropsychoph 35: 881–892. 10.1038/npp.2009.176 PMC3055369 19907417

[bibr383-0269881120959637] Meneses A (1999) 5-HT system and cognition. Neurosci Biobehav Rev 23: 1111–1125. 10643820 10.1016/s0149-7634(99)00067-6

[bibr384-0269881120959637] Menon JM Nolten C Achterberg EM, et al. (2019) Brain microdialysate monoamines in relation to circadian rhythms, sleep, and sleep deprivation–a systematic review, network meta-analysis, and new primary data. J Circadian Rhythm 17: 1. 10.5334/jcr.174 PMC6337052 30671123

[bibr385-0269881120959637] Meyer JH McMain S Kennedy SH, et al. (2003) Dysfunctional attitudes and 5-HT2 receptors during depression and self-harm. Am J Psychiatry 160: 90–99. 12505806 10.1176/appi.ajp.160.1.90

[bibr386-0269881120959637] Meyhöfer I Kumari V Hill A, et al. (2017) Sleep deprivation as an experimental model system for psychosis: Effects on smooth pursuit, prosaccades, and antisaccades. J Psychopharmacol 31: 418–433. 28347256 10.1177/0269881116675511

[bibr387-0269881120959637] Miller MW Sperbeck E Robinson ME, et al. (2016) 5-HT2A gene variants moderate the association between PTSD and reduced default mode network connectivity. Front Neurosci 10: 299. 27445670 10.3389/fnins.2016.00299 PMC4923242

[bibr388-0269881120959637] Miller WR C’de Baca J (2001) Quantum Change: When Epiphanies and Sudden Insights Transform Ordinary Lives. New York: Guilford Press.

[bibr389-0269881120959637] Millière R (2017) Looking for the self: Phenomenology, neurophysiology and philosophical significance of drug-induced ego dissolution. Front Hum Neurosci 11: 245. 28588463 10.3389/fnhum.2017.00245 PMC5441112

[bibr390-0269881120959637] Millière R Carhart-Harris RL Roseman L, et al. (2018) Psychedelics, meditation, and self-consciousness. Front Psychol 9: 1475. 10.3389/fpsyg.2018.01475 PMC6137697 30245648

[bibr391-0269881120959637] Minichino A Delle Chiaie R Cruccu G, et al. (2016) Pain-processing abnormalities in bipolar I disorder, bipolar II disorder, and schizophrenia: A novel trait marker for psychosis proneness and functional outcome? Bipolar Disord 18: 591–601. 27782355 10.1111/bdi.12439

[bibr392-0269881120959637] Mishlove M Chapman LJ (1985) Social anhedonia in the prediction of psychosis proneness. J Abnorm Psychol 94: 384–396. 4031235 10.1037//0021-843x.94.3.384

[bibr393-0269881120959637] Miyazaki K Miyazaki KW Doya K (2012) The role of serotonin in the regulation of patience and impulsivity. Mol Neurobiol 45: 213–224. 22262065 10.1007/s12035-012-8232-6 PMC3311865

[bibr394-0269881120959637] Miyazaki K Miyazaki KW Yamanaka A, et al. (2018) Reward probability and timing uncertainty alter the effect of dorsal raphe serotonin neurons on patience. Nat Commun 9: 2048. 10.1038/s41467-018-04496-y PMC5984631 29858574

[bibr395-0269881120959637] Miyazaki KW Miyazaki K Tanaka KF (2014) Optogenetic activation of dorsal raphe serotonin neurons enhances patience for future rewards. Curr Biol 24: 2033–2040. 25155504 10.1016/j.cub.2014.07.041

[bibr396-0269881120959637] Mohanty D Sarai S Naik S, et al. (2019) Pimavanserin for Parkinson disease psychosis. Prim Care Companion CNS Disord 21: 18l02355. 10.4088/PCC.18l02355 31050230

[bibr397-0269881120959637] Møller P Husby R (2000) The initial prodrome in schizophrenia: Searching for naturalistic core dimensions of experience and behavior. Schizophrenia Bull 26: 217–232. 10.1093/oxfordjournals.schbul.a033442 10755683

[bibr398-0269881120959637] Møller P Husby R (2003) The initial prodrome in schizophrenia–core dimensions of experience and behavior. Tidsskrift for den Norske laegeforening: tidsskrift for praktisk medicin, ny raekke 123: 2425–2429. 14594048

[bibr399-0269881120959637] Moncrieff J (2008) The Myth of the Chemical Cure: A Critique of Psychiatric Drugs. New York: Palgrave Macmillan.

[bibr400-0269881120959637] Monti JM Jantos H (2006) Effects of activation and blockade of 5-HT2A/2C receptors in the dorsal raphe nucleus on sleep and waking in the rat. Prog Neuropsychopharmacol Biol Psychiatry 30(7): 1189–1195. 16713054 10.1016/j.pnpbp.2006.02.013

[bibr401-0269881120959637] Monti JM Perumal SRP Spence DW, et al. (2018) The involvement of 5-HT 2A receptor in the regulation of sleep and wakefulness, and the potential therapeutic use of selective 5-HT 2A receptor antagonists and inverse agonists for the treatment of an insomnia disorder. In: Guiard BP Di Giovanni G (eds) 5-HT2A Receptors in the Central Nervous System. Totowa: Humana Press, pp. 311–337.

[bibr402-0269881120959637] Moreno FA Wiegand CB Taitano EK, et al. (2006) Safety, tolerability, and efficacy of psilocybin in 9 patients with obsessive-compulsive disorder. J Clin Psychiat 67: 1735–1740. 10.4088/jcp.v67n1110 17196053

[bibr403-0269881120959637] Moreno JL González-Maeso J (2018) Crosstalk receptors: Between implications 5-HT in 2A and schizophrenia mGlu2 and its treatment. In: Guiard BP Di Giovanni G (eds) 5-HT2A Receptors in the Central Nervous System. Totowa: Humana Press, pp. 147–189.

[bibr404-0269881120959637] Morici JF Miranda M Gallo FT, et al. (2018) 5-HT2a receptor in mPFC influences context-guided reconsolidation of object memory in perirhinal cortex. eLife 7: e33746. 29717980 10.7554/eLife.33746 PMC5931799

[bibr405-0269881120959637] Morylowska-Topolska J Ziemiński R Molas A, et al. (2017) Schizophrenia and anorexia nervosa-reciprocal relationships. A literature review. Psychiatr Pol 51: 261–270. 28581536 10.12740/PP/OnlineFirst/63514

[bibr406-0269881120959637] Muguruza C Miranda-Azpiazu P Díez-Alarcia R, et al. (2014) Evaluation of 5-HT2A and mGlu2/3 receptors in postmortem prefrontal cortex of subjects with major depressive disorder: Effect of antidepressant treatment. Neuropharmacology 86: 311–318. 25150943 10.1016/j.neuropharm.2014.08.009 PMC12706166

[bibr407-0269881120959637] Murakami N Sakai Y Ooki S (1980) Behavioral thermoregulation in rats during hyperthermia induced by lysergic acid diethylamide. Neurosci Lett 20: 105–108. 7052543 10.1016/0304-3940(80)90242-6

[bibr408-0269881120959637] Murnane KS (2019) Serotonin 2A receptors are a stress response system: Implications for post-traumatic stress disorder. Behav Pharmacol 30: 151–162. 30632995 10.1097/FBP.0000000000000459 PMC6422730

[bibr409-0269881120959637] Murphy-Beiner A Soar K (2020) Ayahuasca’s ‘afterglow’: Improved mindfulness and cognitive flexibility in ayahuasca drinkers. Psychopharmacology 237: 1161–1169. 31927605 10.1007/s00213-019-05445-3

[bibr410-0269881120959637] Murray ED Cunningham MG Price BH (2012) The role of psychotic disorders in religious history considered. J Neuropsych Clin N 24: 410–426. 10.1176/appi.neuropsych.11090214 23224447

[bibr411-0269881120959637] Myers RD Beleslin DB (1971) Changes in serotonin release in hypothalamus during cooling or warming of the monkey. Am J Physiol 220: 1746–1754. 4997208 10.1152/ajplegacy.1971.220.6.1746

[bibr412-0269881120959637] Nakagawa H Matsumura T Suzuki K, et al. (2016) Changes of brain monoamine levels and physiological indexes during heat acclimation in rats. J Therm Biol 58: 15–22. 27157329 10.1016/j.jtherbio.2016.03.010

[bibr413-0269881120959637] Nakajima K Obata H Ito N, et al. (2009) The nociceptive mechanism of 5-hydroxytryptamine released into the peripheral tissue in acute inflammatory pain in rats. Eur J Pain 13: 441–447. 18656400 10.1016/j.ejpain.2008.06.007

[bibr414-0269881120959637] Naor L Mayseless O (2017) How personal transformation occurs following a single peak experience in nature: A phenomenological account. J Humanist Psychol. Epub ahead of print 23 June. DOI: 10.1177/0022167817714692.

[bibr415-0269881120959637] Napier TC Istre ED (2008) Methamphetamine-induced sensitization includes a functional upregulation of ventral pallidal 5-HT2A/2C receptors. Synapse 62(1): 14–21. 17957734 10.1002/syn.20460

[bibr416-0269881120959637] Nasrallah HA Fedora R Morton R (2019) Successful treatment of clozapine-nonresponsive refractory hallucinations and delusions with pimavanserin, a serotonin 5HT-2A receptor inverse agonist. Schizophr Res 208: 217–220. 30837203 10.1016/j.schres.2019.02.018

[bibr417-0269881120959637] Nau F Jr Yu B Martin D, et al. (2013) Serotonin 5-HT2A receptor activation blocks TNF-α mediated inflammation in vivo. PLoS One 8: e75426. 24098382 10.1371/journal.pone.0075426 PMC3788795

[bibr418-0269881120959637] Nelson B Sass LA Parnas J (2016) Basic self disturbance in the schizophrenia spectrum: A review and future directions. In: Kyrios, et al. (eds) The Self in Understanding and Treating Psychological Disorders. Cambridge: Cambridge University Press, pp. 158–168.

[bibr419-0269881120959637] Neugebauer V (2020) Serotonin—pain modulation. In: Müller CP and Cunningham KA (eds) Handbook of Behavioral Neuroscience, vol. 31. Elsevier, pp. 309–320.

[bibr420-0269881120959637] Ng LC Petruzzi LJ Greene MC, et al. (2016) Posttraumatic stress disorder symptoms and social and occupational functioning of people with schizophrenia. J Nerv Ment Dis 204: 590–598. 27105458 10.1097/NMD.0000000000000523 PMC4969152

[bibr421-0269881120959637] Nichols CD Nichols DE (2019) DMT in the Mammalian Brain: A Critical Appraisal. Available at: https://www.aliusresearch.org/nichols-nichols-endogenous-dmt.html

[bibr422-0269881120959637] Nichols DE (2016) Psychedelics. Pharmacol Rev 68: 264–355. 26841800 10.1124/pr.115.011478 PMC4813425

[bibr423-0269881120959637] Niitsu Y Hamada S Hamaguchi K (1995) Regulation of synapse density by 5-HT2A receptor agonist and antagonist in the spinal cord of chicken embryo. Neurosci Lett 195: 159–162. 8584199 10.1016/0304-3940(95)11805-7

[bibr424-0269881120959637] Nisijima K Yoshino T Yui K, et al. (2001) Potent serotonin (5-HT)(2A) receptor antagonists completely prevent the development of hyperthermia in an animal model of the 5-HT syndrome. Brain Res 890: 23–31. 11164765 10.1016/s0006-8993(00)03020-1

[bibr425-0269881120959637] Nivethitha L Mooventhan A Manjunath NK, et al. (2017) Cerebrovascular hemodynamics during pranayama techniques. J Neurosci Rur Pract 8: 60. 10.4103/0976-3147.193532 PMC5225724 28149083

[bibr426-0269881120959637] Nour MM Evans L Carhart-Harris RL (2017) Psychedelics, personality and political perspectives. J Psychoactive Drugs 49: 182–191. 28443703 10.1080/02791072.2017.1312643

[bibr427-0269881120959637] Nour MM Evans L Nutt D, et al. (2016) Ego-dissolution and psychedelics: Validation of the ego-dissolution inventory (EDI). Front Hum Neurosci 10: 269. 27378878 10.3389/fnhum.2016.00269 PMC4906025

[bibr428-0269881120959637] Nutt D Erritzoe D Carhart-Harris R (2020) Psychedelic psychiatry’s brave new world. Cell 181: 24–28. 32243793 10.1016/j.cell.2020.03.020

[bibr429-0269881120959637] Nutt DJ King LA Nichols DE (2013) Effects of schedule I drug laws on neuroscience research and treatment innovation. Nat Rev Neurosci 14: 577. 23756634 10.1038/nrn3530

[bibr430-0269881120959637] Ohta KI Miki T Warita K, et al. (2014) Prolonged maternal separation disturbs the serotonergic system during early brain development. Int J Dev Neurosci 33: 15–21. 24184298 10.1016/j.ijdevneu.2013.10.007

[bibr431-0269881120959637] Olivier B Mos J (1990) Serenics, serotonin and aggression. Prog Clin Biol Res 361: 203–230. 1981258

[bibr432-0269881120959637] Olson C (ed.) (2008) Celibacy and Religious Traditions. New York: Oxford University Press.

[bibr433-0269881120959637] Olson DE (2018) Psychoplastogens: A promising class of plasticity-promoting neurotherapeutics. J Exp Neurosci 12: 1179069518800508. 30262987 10.1177/1179069518800508 PMC6149016

[bibr434-0269881120959637] Olthof M Hasselman F Strunk G, et al. (2019) Destabilization in self-ratings of the psychotherapeutic process is associated with better treatment outcome in patients with mood disorders. Psychother Res 30: 520–531. 31256713 10.1080/10503307.2019.1633484

[bibr435-0269881120959637] Omachi Y Sumiyoshi T (2018) Dose reduction/discontinuation of antipsychotic drugs in psychosis; Effect on cognition and functional outcomes. Front Psychiat 9: 447. 10.3389/fpsyt.2018.00447 PMC6158366 30294286

[bibr436-0269881120959637] O’Mara S (2015) Why Torture Doesn’t Work. Boston, MA: Harvard University Press.

[bibr437-0269881120959637] Osório FDL Sanches RF Macedo LR, et al. (2015) Antidepressant effects of a single dose of ayahuasca in patients with recurrent depression: A preliminary report. Braz J Psychiat 37: 13–20. 10.1590/1516-4446-2014-1496 25806551

[bibr438-0269881120959637] Ossowska G Nowak G Kata R, et al. (2001) Brain monoamine receptors in a chronic unpredictable stress model in rats. J Neural Transm 108: 311–319. 11341483 10.1007/s007020170077

[bibr439-0269881120959637] Ott U Reuter M Hennig J, et al. (2005) Evidence for a common biological basis of the absorption trait, hallucinogen effects, and positive symptoms: Epistasis between 5-HT2a and COMT polymorphisms. Am J Med Genet B 137: 29–32. 10.1002/ajmg.b.30197 15965969

[bibr440-0269881120959637] Owens MJ Knight DL Ritchie JC, et al. (1991) The 5-hydroxytryptamine2 agonist, (+-)-1-(2,5-dimethoxy-4-bromophenyl)-2-aminopropane stimulates the hypothalamic-pituitary-adrenal (HPA) axis. I. Acute effects on HPA axis activity and corticotropin-releasing factor-containing neurons in the rat brain. J Pharmacol Exp Ther 256: 787–794. 1847212

[bibr441-0269881120959637] Pandey GN Dwivedi Y Rizavi HS, et al. (2002) Higher expression of serotonin 5-HT2A receptors in the postmortem brains of teenage suicide victims. Am J Psychiatry 159: 419–429. 11870006 10.1176/appi.ajp.159.3.419

[bibr442-0269881120959637] Paolini E Moretti P Compton MT (2016) Delusions in first-episode psychosis: Principal component analysis of twelve types of delusions and demographic and clinical correlates of resulting domains. Psychiat Res 243: 5–13. 10.1016/j.psychres.2016.06.002 PMC5014642 27344587

[bibr443-0269881120959637] Parade SH Novick AM Parent J, et al. (2017) Stress exposure and psychopathology alter methylation of the serotonin receptor 2A (HTR2A) gene in preschoolers. Dev Psychopathol 29: 1619–1626. 29162169 10.1017/S0954579417001274 PMC5720374

[bibr444-0269881120959637] Parnas J Handest P (2003) Phenomenology of anomalous self-experience in early schizophrenia. Compr Psychiat 44: 121–134. 12658621 10.1053/comp.2003.50017

[bibr445-0269881120959637] Parnas J Henriksen MG (2016) Mysticism and schizophrenia: A phenomenological exploration of the structure of consciousness in the schizophrenia spectrum disorders. Conscious Cogn 43: 75–88. 27258928 10.1016/j.concog.2016.05.010

[bibr446-0269881120959637] Partti K Vasankari T Kanervisto M, et al. (2015) Lung function and respiratory diseases in people with psychosis: Population-based study. Brit J Psychiat 207: 37–45. 10.1192/bjp.bp.113.141937 25858177

[bibr447-0269881120959637] Pasquini L Palhano-Fontes F Araujo DB (2020) Subacute effects of the psychedelic ayahuasca on the salience and default mode networks. J Psychopharmacol 34: 623–635. 32255395 10.1177/0269881120909409

[bibr448-0269881120959637] Paul ED Hale MW Lukkes JL, et al. (2011) Repeated social defeat increases reactive emotional coping behavior and alters functional responses in serotonergic neurons in the rat dorsal raphe nucleus. Physiol Behav 104: 272–282. 21238469 10.1016/j.physbeh.2011.01.006 PMC3089807

[bibr449-0269881120959637] Paul LA (2014) Transformative Experience. Oxford: Oxford University Press.

[bibr450-0269881120959637] Pawlyk AC Cosmi S Alfinito PD, et al. (2006) Effects of the 5-HT2A antagonist mirtazapine in rat models of thermoregulation. Brain Res 1123: 135–144. 17067560 10.1016/j.brainres.2006.09.050

[bibr451-0269881120959637] Pehek EA Hernan AE (2015) Stimulation of glutamate receptors in the ventral tegmental area is necessary for serotonin-2 receptor-induced increases in mesocortical dopamine release. Neurosci 290: 159–164. 10.1016/j.neuroscience.2015.01.029 PMC5329887 25637799

[bibr452-0269881120959637] Pehek EA Nocjar C Roth BL, et al. (2006) Evidence for the preferential involvement of 5-HT2A serotonin receptors in stress-and drug-induced dopamine release in the rat medial prefrontal cortex. Neuropsychoph 31: 265–277. 10.1038/sj.npp.1300819 15999145

[bibr453-0269881120959637] Peričić D (2003) Swim stress inhibits 5-HT 2A receptor-mediated head twitch behaviour in mice. Psychopharmacology 167: 373–379. 12695874 10.1007/s00213-002-1357-y

[bibr454-0269881120959637] Perry JW (1977) Psychosis and the visionary mind. J Alter St Conscious 3: 5–14.

[bibr455-0269881120959637] Petri G Expert P Turkheimer F, et al. (2014) Homological scaffolds of brain functional networks. J R Soc Interface 11: 20140873. 25401177 10.1098/rsif.2014.0873 PMC4223908

[bibr456-0269881120959637] Picardi A Fonzi L Pallagrosi M, et al. (2018) Delusional themes across affective and non-affective psychoses. Front Psychiat 9: 132. 10.3389/fpsyt.2018.00132 PMC5895977 29674982

[bibr457-0269881120959637] Pillinger T Beck K Gobjila C, et al. (2017) Impaired glucose homeostasis in first-episode schizophrenia: A systematic review and meta-analysis. JAMA Psychiat 74: 261–269. 10.1001/jamapsychiatry.2016.3803 PMC6352957 28097367

[bibr458-0269881120959637] Piszczek L Piszczek A Kuczmanska J, et al. (2015) Modulation of anxiety by cortical serotonin 1A receptors. Front Behav Neurosci 9: 48. 25759645 10.3389/fnbeh.2015.00048 PMC4338812

[bibr459-0269881120959637] Pitts EG Minerva AR Chandler EB, et al. (2017) 3, 4-Methylenedioxymethamphetamine increases affiliative behaviors in squirrel monkeys in a serotonin 2A receptor-dependent manner. Neuropsychoph 42: 1962–1971. 10.1038/npp.2017.80 PMC5561347 28425496

[bibr460-0269881120959637] Plitman E Patel R Chung JK, et al. (2016) Glutamatergic metabolites, volume and cortical thickness in antipsychotic-naive patients with first-episode psychosis: Implications for excitotoxicity. Neuropsychoph 41: 2606. 10.1038/npp.2016.84 PMC4987861 27272768

[bibr461-0269881120959637] Pokorny T Preller KH Kometer M, et al. (2017) Effect of psilocybin on empathy and moral decision-making. Int J Neuropsychoph 20: 747–757. 10.1093/ijnp/pyx047 PMC5581487 28637246

[bibr462-0269881120959637] Polimeni J (2018) Psychosis is episodically required for the enduring integrity of shamanism. Behav Brain Sci 41: e82. 31064457 10.1017/S0140525X17002138

[bibr463-0269881120959637] Pollan M (2019) How to Change Your Mind: What the New Science of Psychedelics Teaches Us about Consciousness, Dying, Addiction, Depression, and Transcendence. New York: Penguin Books. 10.1080/02791072.2018.1535149 30376643

[bibr464-0269881120959637] Powers AR Corlett PR (2018) Shamanism and psychosis: Shared mechanisms? Behav Brain Sci 41: e83. 31064450 10.1017/S0140525X1700214X

[bibr465-0269881120959637] Prabakaran S Swatton JE Ryan MM, et al. (2004) Mitochondrial dysfunction in schizophrenia: Evidence for compromised brain metabolism and oxidative stress. Mol Psychiatr 9: 684–697. 10.1038/sj.mp.4001511 15098003

[bibr466-0269881120959637] Preece MA Dalley JW Theobald DEH, et al. (2004) Region specific changes in forebrain 5-hydroxytryptamine1A and 5-hydroxytryptamine2A receptors in isolation-reared rats: An in vitro autoradiography study. Neuroscience 123: 725–732. 14706784 10.1016/j.neuroscience.2003.10.008

[bibr467-0269881120959637] Preller KH Burt JB Ji JL, et al. (2018) Changes in global and thalamic brain connectivity in LSD-induced altered states of consciousness are attributable to the 5-HT2A receptor. eLife 7: e35082. 30355445 10.7554/eLife.35082 PMC6202055

[bibr468-0269881120959637] Preller KH Herdener M Pokorny T, et al. (2017) The fabric of meaning and subjective effects in LSD-induced states depend on serotonin 2A receptor activation. Curr Biol 27: 451–457. 28132813 10.1016/j.cub.2016.12.030

[bibr469-0269881120959637] Quednow BB Geyer MA Halberstadt AL (2020) Serotonin and schizophrenia. In: Müller CP and Cunningham KA (eds) Handbook of Behavioral Neuroscience, vol. 31. Elsevier, pp. 711–743.

[bibr470-0269881120959637] Quednow BB Kometer M Geyer MA, et al. (2012) Psilocybin-induced deficits in automatic and controlled inhibition are attenuated by ketanserin in healthy human volunteers. Neuropsychoph 37: 630–640. 10.1038/npp.2011.228 PMC3260978 21956447

[bibr471-0269881120959637] Raine A (1991) The SPQ: A scale for the assessment of schizotypal personality based on DSM-III-R criteria. Schizophrenia Bull 17: 555–564. 10.1093/schbul/17.4.555 1805349

[bibr472-0269881120959637] Raine A Reynolds C Lencz T, et al. (1994) Cognitive-perceptual, interpersonal, and disorganized features of schizotypal personality. Schizophrenia Bull 20: 191–201. 10.1093/schbul/20.1.191 8197415

[bibr473-0269881120959637] Rajapakse T Garcia-Rosales A Weerawardene S, et al. (2011) Themes of delusions and hallucinations in first-episode psychosis. Early Interv Psychia 5: 254–258. 10.1111/j.1751-7893.2011.00281.x 21707940

[bibr474-0269881120959637] Rambo LR (1993) Understanding Religious Conversion. New Haven, CT: Yale University Press.

[bibr475-0269881120959637] Ranade S Pi HJ Kepecs A (2014) Neuroscience: Waiting for serotonin. Curr Biol 24: R803–805. 25202872 10.1016/j.cub.2014.07.024

[bibr476-0269881120959637] Rao RP Ballard DH (1999) Predictive coding in the visual cortex: A functional interpretation of some extra-classical receptive-field effects. Nat Neurosci 2: 79–87. 10195184 10.1038/4580

[bibr477-0269881120959637] Raote I Bhattacharya A Panicker MM (2007) Serotonin 2A (5-HT2A) receptor function: Ligand-dependent mechanisms and pathways. In: Chattopadhyay (ed.) Serotonin Receptors in Neurobiology. Boca Raton, FL: CRC Press, chapter 6. 21204452

[bibr478-0269881120959637] Rapoport JL Gogtay N (2008) Brain neuroplasticity in healthy, hyperactive and psychotic children: Insights from neuroimaging. Neuropsychoph 33: 181–197. 10.1038/sj.npp.1301553 17851542

[bibr479-0269881120959637] Rasmussen H Erritzoe D Andersen R, et al. (2010) Decreased frontal serotonin2A receptor binding in antipsychotic-naive patients with first-episode schizophrenia. Arch Gen Psychiat 67: 9–16. 20048218 10.1001/archgenpsychiatry.2009.176

[bibr480-0269881120959637] Rasmussen H Frokjaer VG Hilker RW, et al. (2016) Low frontal serotonin 2A receptor binding is a state marker for schizophrenia? Eur Neuropsychopharm 26: 1248–1250. 10.1016/j.euroneuro.2016.04.008 27179966

[bibr481-0269881120959637] Rasmussen K Glennon RA Aghajanian GK (1986) Phenethylamine hallucinogens in the locus coeruleus: Potency of action correlates with rank order of 5-HT2 binding affinity. Eur J Pharmacol 132: 79–82. 3816969 10.1016/0014-2999(86)90014-2

[bibr482-0269881120959637] Reeve S Sheaves B Freeman D (2018) Sleep disorders in early psychosis: Incidence, severity, and association with clinical symptoms. Schizophrenia Bull 45: 287–295. 10.1093/schbul/sby129 PMC6403049 30202909

[bibr483-0269881120959637] Reininghaus UA Morgan C Simpson J, et al. (2008) Unemployment, social isolation, achievement–expectation mismatch and psychosis: Findings from the ÆSOP Study. Soc Psych Psych Epid 43: 743–751. 10.1007/s00127-008-0359-4 18491023

[bibr484-0269881120959637] Rekhi G Rapisarda A Lee J (2019) Impact of distress related to attenuated psychotic symptoms in individuals at ultra high risk of psychosis: Findings from the Longitudinal Youth at Risk Study. Early Interv Psychia 13: 73–78. 10.1111/eip.12451 28560723

[bibr485-0269881120959637] Reneman L Endert E de Bruin K, et al. (2002) The acute and chronic effects of MDMA (‘ecstasy’) on cortical 5-HT 2A receptors in rat and human brain. Neuropsychoph 26: 387–396. 10.1016/S0893-133X(01)00366-9 11850153

[bibr486-0269881120959637] Rex A Voigt JP Fink H (2005) Anxiety but not arousal increases 5-hydroxytryptamine release in the rat ventral hippocampus in vivo. Eur J Neurosci 22: 1185–1189. 16176361 10.1111/j.1460-9568.2005.04251.x

[bibr487-0269881120959637] Rhodes RF (1987) The ‘Kaihōgyō’ Practice of Mt. Hiei. Jpn J Relig Stud 14: 185–202.

[bibr488-0269881120959637] Richards WA (2015) Sacred Knowledge: Psychedelics and Religious Experiences. New York: Columbia University Press.

[bibr489-0269881120959637] Richerson GB (2004) Serotonergic neurons as carbon dioxide sensors that maintain pH homeostasis. Nat Rev Neurosci 5: 449–461. 15152195 10.1038/nrn1409

[bibr490-0269881120959637] Richtand NM Welge JA Logue AD, et al. (2007) Dopamine and serotonin receptor binding and antipsychotic efficacy. Neuropsychoph 32: 1715–1726. 10.1038/sj.npp.1301305 17251913

[bibr491-0269881120959637] Rietdijk J Ising HK Dragt S, et al. (2013) Depression and social anxiety in help-seeking patients with an ultra-high risk for developing psychosis. Psychiat Res 209: 309–313. 10.1016/j.psychres.2013.01.012 23433870

[bibr492-0269881120959637] Rilke O Freier D Jähkel M, et al. (1998) Dynamic alterations of serotonergic metabolism and receptors during social isolation of low-and high-active mice. Pharmacol Biochem Be 59: 891–896. 10.1016/s0091-3057(97)00509-1 9586845

[bibr493-0269881120959637] Rillich J Stevenson PA (2018) Serotonin mediates depression of aggression after acute and chronic social defeat stress in a model insect. Front Behav Neurosci 12: 233. 30349464 10.3389/fnbeh.2018.00233 PMC6186776

[bibr494-0269881120959637] Rochefort C Baldwin AS Chmielewski M (2018) Experiential avoidance: An examination of the construct validity of the AAQ-II and MEAQ. Behav Ther 49: 435–449. 29704971 10.1016/j.beth.2017.08.008

[bibr495-0269881120959637] Roemer L Litz BT Orsillo SM, et al. (2001) A preliminary investigation of the role of strategic withholding of emotions in PTSD. J Trauma Stress 14: 149–156.

[bibr496-0269881120959637] Romano AG Quinn JL Li L, et al. (2010) Intrahippocampal LSD accelerates learning and desensitizes the 5-HT(2A) receptor in the rabbit. Psychopharmacology 212: 441–448. 20827462 10.1007/s00213-010-2004-7

[bibr497-0269881120959637] Roseman L Haijen E Idialu-Ikato K, et al. (2019) Emotional breakthrough and psychedelics: Validation of the Emotional Breakthrough Inventory. J Psychopharmacol 33: 1076–1087. 31294673 10.1177/0269881119855974

[bibr498-0269881120959637] Roseman L Nutt DJ Carhart-Harris RL (2018) Quality of acute psychedelic experience predicts therapeutic efficacy of psilocybin for treatment-resistant depression. Front Pharmacol 8: 974. 29387009 10.3389/fphar.2017.00974 PMC5776504

[bibr499-0269881120959637] Ross RM McKay R (2018) Shamanism and the psychosis continuum. Behav Brain Sci 41: e84. 31064531 10.1017/S0140525X17002151

[bibr500-0269881120959637] Ross S Bossis A Guss J, et al. (2016) Rapid and sustained symptom reduction following psilocybin treatment for anxiety and depression in patients with life-threatening cancer: A randomized controlled trial. J Psychopharmacol 30: 1165–1180. 27909164 10.1177/0269881116675512 PMC5367551

[bibr501-0269881120959637] Roth WT Gomolla A Meuret AE, et al. (2002) High altitudes, anxiety, and panic attacks: Is there a relationship? Depress Anxiety 16: 51–58. 12219335 10.1002/da.10059

[bibr502-0269881120959637] Rovelli C (2018) Reality Is Not What It Seems: The Journey to Quantum Gravity. New York: Penguin.

[bibr503-0269881120959637] Rucker JJ Iliff J Nutt DJ (2018) Psychiatry & the psychedelic drugs. Past, present & future. Neuropharmacology 142: 200–218. 29284138 10.1016/j.neuropharm.2017.12.040

[bibr504-0269881120959637] Sadzot B Baraban JM Glennon RA, et al. (1989) Hallucinogenic drug interactions at human brain 5-HT 2 receptors: Implications for treating LSD-induced hallucinogenesis. Psychopharmacology 98: 495–499. 2505289 10.1007/BF00441948

[bibr505-0269881120959637] Saito T Ishiwata T Hasegawa H, et al. (2005) Changes in monoamines in rat hypothalamus during cold acclimation. J Therm Biol 30: 229–235.

[bibr506-0269881120959637] Sakaue M Ago Y Sowa C, et al. (2002) Modulation by 5-HT2A receptors of aggressive behavior in isolated mice. Jpn J Pharmacol 89: 89–92. 12083749 10.1254/jjp.89.89

[bibr507-0269881120959637] Sakr AH (1975) Fasting in Islam. J Am Diet Assoc 67: 17–21. 1141609

[bibr508-0269881120959637] Salmi P Ahlenius S (1998) Evidence for functional interactions between 5-HT1A and 5-HT2A receptors in rat thermoregulatory mechanisms. Pharmacol Toxicol 82: 122–127. 9553989 10.1111/j.1600-0773.1998.tb01410.x

[bibr509-0269881120959637] Salo J Jokela M Lehtimäki T, et al. (2011) Serotonin receptor 2A gene moderates the effect of childhood maternal nurturance on adulthood social attachment. Genes Brain Behav 10: 702–709. 21649857 10.1111/j.1601-183X.2011.00708.x

[bibr510-0269881120959637] Salvation (n.d.) [Def. 3 a.b.] Merriam-Webster.com. Available at: https://www.merriam-webster.com/dictionary/salvation (accessed 13 August 2019).

[bibr511-0269881120959637] Sanz C Zamberlan F Erowid E, et al. (2018) The experience elicited by hallucinogens presents the highest similarity to dreaming within a large database of psychoactive substance reports. Front Neurosci 12: 7. 29403350 10.3389/fnins.2018.00007 PMC5786560

[bibr512-0269881120959637] Savage C (1955) Variations in ego feeling induced by D-lysergic acid diethylamide (LSD-25). Psychoanal Rev 42: 1–16. 14371878

[bibr513-0269881120959637] Savage C Cholden L (1956) Schizophrenia and the model psychoses. J Clin Exp Psychopat 17: 405–412. 13406030

[bibr514-0269881120959637] Schiller L Jähkel M Kretzschmar M, et al. (2003) Autoradiographic analyses of 5-HT1A and 5-HT2A receptors after social isolation in mice. Brain Res 980: 169–178. 12867255 10.1016/s0006-8993(03)02832-4

[bibr515-0269881120959637] Schindler EA Wallace RM Sloshower JA, et al. (2018) Neuroendocrine associations underlying the persistent therapeutic effects of classic serotonergic psychedelics. Front Pharmacol 9: 177. 29545753 10.3389/fphar.2018.00177 PMC5838010

[bibr516-0269881120959637] Schirmbeck F Zink M (2012) Clozapine-induced obsessive-compulsive symptoms in schizophrenia: A critical review. Curr Neuropharmacol 10: 88–95. 22942882 10.2174/157015912799362724 PMC3286851

[bibr517-0269881120959637] Schlosser M Sparby T Vörös S, et al. (2019) Unpleasant meditation-related experiences in regular meditators: Prevalence, predictors, and conceptual considerations. PLoS One 14: e0216643. 31071152 10.1371/journal.pone.0216643 PMC6508707

[bibr518-0269881120959637] Schmid CL Streicher JM Meltzer HY, et al. (2014) Clozapine acts as an agonist at serotonin 2A receptors to counter MK-801-induced behaviors through a βarrestin2-independent activation of Akt. Neuropsychoph 39: 1902. 10.1038/npp.2014.38 PMC4059899 24531562

[bibr519-0269881120959637] Schmid Y Enzler F Gasser P, et al. (2015) Acute effects of lysergic acid diethylamide in healthy subjects. Biol Psychiatry 78: 544–553. 25575620 10.1016/j.biopsych.2014.11.015

[bibr520-0269881120959637] Seeman MV (2017) Solitude and schizophrenia. Psychosis 9: 176–183.

[bibr521-0269881120959637] Sellers R Varese F Wells A, et al. (2017) A meta-analysis of metacognitive beliefs as implicated in the self-regulatory executive function model in clinical psychosis. Schizophr Res 179: 75–84. 27670237 10.1016/j.schres.2016.09.032

[bibr522-0269881120959637] Selten JP Cantor-Graae E (2005) Social defeat: Risk factor for schizophrenia? Brit J Psychiat 187: 101–102. 10.1192/bjp.187.2.101 16055818

[bibr523-0269881120959637] Selten JP Cantor-Graae E (2007) Hypothesis: Social defeat is a risk factor for schizophrenia? Brit J Psychiat 191(S51): s9–s12. 10.1192/bjp.191.51.s9 18055945

[bibr524-0269881120959637] Selten JP van der Ven E Rutten BP, et al. (2013) The social defeat hypothesis of schizophrenia: An update. Schizophrenia Bull 39: 1180–1186. 10.1093/schbul/sbt134 PMC3796093 24062592

[bibr525-0269881120959637] Selten JP van Os J Cantor-Graae E (2016) The social defeat hypothesis of schizophrenia: Issues of measurement and reverse causality. World Psychiatry 15: 294–295. 27717278 10.1002/wps.20369 PMC5032495

[bibr526-0269881120959637] Sessa B Higbed L Nutt D (2019) A review of 3, 4-methylenedioxymethamphetamine (MDMA)-assisted psychotherapy. Front Psychiat 10: 138. 10.3389/fpsyt.2019.00138 PMC6435835 30949077

[bibr527-0269881120959637] Seth AK (2013) Interoceptive inference, emotion, and the embodied self. Trends Cogn Sci 17: 565–573. 24126130 10.1016/j.tics.2013.09.007

[bibr528-0269881120959637] Seyrek M Kahraman S Deveci MS, et al. (2010) Systemic cannabinoids produce CB1-mediated antinociception by activation of descending serotonergic pathways that act upon spinal 5-HT7 and 5-HT2A receptors. Eur J Pharmacol 649: 183–194. 20868676 10.1016/j.ejphar.2010.09.039

[bibr529-0269881120959637] Shanon B (2002) The Antipodes of the Mind: Charting the Phenomenology of the Ayahuasca Experience. New York: Oxford University Press.

[bibr530-0269881120959637] Shelton RC Sanders-Bush E Manier DH, et al. (2009) Elevated 5-HT 2A receptors in postmortem prefrontal cortex in major depression is associated with reduced activity of protein kinase A. Neuroscience 158: 1406–1415. 19111907 10.1016/j.neuroscience.2008.11.036 PMC9208662

[bibr531-0269881120959637] Siegel RK (1986) MDMA: Nonmedical use and intoxication. J Psychoactive Drugs 18: 349–354. 2880950 10.1080/02791072.1986.10472368

[bibr532-0269881120959637] Singh M (2018) The cultural evolution of shamanism. Behav Brain Sci 41: 1–62. 10.1017/S0140525X17001893 28679454

[bibr533-0269881120959637] Sinh V Ootsuka Y (2019) Blockade of 5-HT2A receptors inhibits emotional hyperthermia in mice. J Physiol Sci 69: 1097–1107. 31432430 10.1007/s12576-019-00703-7 PMC10717664

[bibr534-0269881120959637] Slade M Rennick-Egglestone S Blackie L, et al. (2019) Post-traumatic growth in mental health recovery: Qualitative study of narratives. BMJ Open 9: e029342. 10.1136/bmjopen-2019-029342 PMC6609070 31256037

[bibr535-0269881120959637] Sloshower J Guss J Krause R, et al. (2020) Psilocybin-assisted therapy of major depressive disorder using acceptance and commitment therapy as a therapeutic frame. J Contextual Behav Sci 15: 12–19.

[bibr536-0269881120959637] Smigielski L Scheidegger M Kometer M, et al. (2019) Psilocybin-assisted mindfulness training modulates self-consciousness and brain default mode network connectivity with lasting effects. Neuroimage 196: 207–215. 30965131 10.1016/j.neuroimage.2019.04.009

[bibr537-0269881120959637] Smith HR Leibold NK Rappoport DA, et al. (2018) Dorsal raphe serotonin neurons mediate CO2-induced arousal from sleep. J Neurosci 38: 1915–1925. 29378860 10.1523/JNEUROSCI.2182-17.2018 PMC5824737

[bibr538-0269881120959637] Soman S Bhattacharya A Panicker MM (2019) Dopamine requires unique residues to signal via the serotonin 2A receptor. Neuroscience 439: 319–331. 30970266 10.1016/j.neuroscience.2019.03.056

[bibr539-0269881120959637] Sood A Pati S Bhattacharya A, et al. (2018) Early emergence of altered 5-HT2A receptor-evoked behavior, neural activation and gene expression following maternal separation. Int J Dev Neurosci 65: 21–28. 29037912 10.1016/j.ijdevneu.2017.10.005

[bibr540-0269881120959637] Spier L (1921) The Sun Dance of the Plains Indians: Its Development and Diffusion (Vol. 16, No. 7). New York: The Trustees.

[bibr541-0269881120959637] Stace WT (1960) Mysticism and Philosophy. London: St. Martin’s Press.

[bibr542-0269881120959637] Stahl SM (2018) Beyond the dopamine hypothesis of schizophrenia to three neural networks of psychosis: Dopamine, serotonin, and glutamate. CNS Spectr 23: 187–191. 29954475 10.1017/S1092852918001013

[bibr543-0269881120959637] Stanley M Mann JJ (1983) Increased serotonin-2 binding sites in frontal cortex of suicide victims. Lancet 321: 214–216. 10.1016/s0140-6736(83)92590-4 6130248

[bibr544-0269881120959637] Steffen E Coyle A (2010) Can ‘sense of presence’ experiences in bereavement be conceptualised as spiritual phenomena? Ment Heal Relig Cult 13: 273–291.

[bibr545-0269881120959637] Steinberg LJ Underwood MD Bakalian MJ, et al. (2019) 5-HT1A receptor, 5-HT2A receptor and serotonin transporter binding in the human auditory cortex in depression. J Psychiatr Neurosci 44: 1–8. 10.1503/jpn.180190 PMC6710086 31120232

[bibr546-0269881120959637] Stephan KE Friston KJ Frith CD (2009) Dysconnection in schizophrenia: From abnormal synaptic plasticity to failures of self-monitoring. Schizophrenia Bull 35: 509–527. 10.1093/schbul/sbn176 PMC2669579 19155345

[bibr547-0269881120959637] Steptoe A Poole L (2016) Control and stress. In: Fink G (ed.) Stress: Concepts, Cognition, Emotion, and Behavior. Cambridge: Academic Press, pp. 73–80.

[bibr548-0269881120959637] Sterling P Eyer J (1988) Allostasis: A new paradigm to explain arousal pathology. In: Fisher S Reason JT (eds) Handbook of Life Stress, Cognition, and Health. Chicester, NY: Wiley, pp.629–649.

[bibr549-0269881120959637] Strajhar P Schmid Y Liakoni E, et al. (2016) Acute effects of lysergic acid diethylamide on circulating steroid levels in healthy subjects. J Neuroendocrinol 28: 12374. 26849997 10.1111/jne.12374

[bibr550-0269881120959637] Strassman RJ (1984) Adverse reactions to psychedelic drugs. A review of the literature. J Nerv Ment Dis 172: 577–595. 6384428 10.1097/00005053-198410000-00001

[bibr551-0269881120959637] Strassman RJ (2000) DMT: The Spirit Molecule: A Doctor’s Revolutionary Research into the Biology of Near-Death and Mystical Experiences. New York: Simon & Schuster.

[bibr552-0269881120959637] Studerus E Gamma A Kometer M, et al. (2012) Prediction of psilocybin response in healthy volunteers. PLoS One 7: e30800. 22363492 10.1371/journal.pone.0030800 PMC3281871

[bibr553-0269881120959637] Suchecki D Tiba PA Machado RB (2012) REM sleep rebound as an adaptive response to stressful situations. Front Neurol 3: 41. 22485105 10.3389/fneur.2012.00041 PMC3317042

[bibr554-0269881120959637] Sudo N Sogawa H Komaki G, et al. (1997) The serotonin-induced elevation of intracellular Ca 2+ in human platelets is enhanced by total fasting. Biol Psychiatry 41: 618–620. 9046994 10.1016/s0006-3223(96)00499-4

[bibr555-0269881120959637] Swiney L Sousa P (2013) When our thoughts are not our own: Investigating agency misattributions using the mind-to-mind paradigm. Conscious Cogn 22: 589–602. 23619312 10.1016/j.concog.2013.03.007

[bibr556-0269881120959637] Sysoeva OV Tonevitsky AG Wackermann J (2010) Genetic determinants of time perception mediated by the serotonergic system. PLoS One 5: e12650. 20862259 10.1371/journal.pone.0012650 PMC2941468

[bibr557-0269881120959637] Szabo A Kovacs A Riba J Djurovic S, et al. (2016) The endogenous hallucinogen and trace amine N, N-dimethyltryptamine (DMT) displays potent protective effects against hypoxia via sigma-1 receptor activation in human primary iPSC-derived cortical neurons and microglia-like immune cells. Front Neurosci 10: 423. 27683542 10.3389/fnins.2016.00423 PMC5021697

[bibr558-0269881120959637] Szlachta M Pabian P Kuśmider M, et al. (2017) Effect of clozapine on ketamine-induced deficits in attentional set shift task in mice. Psychopharmacology 234: 2103–2112. 28405711 10.1007/s00213-017-4613-x PMC5486929

[bibr559-0269881120959637] Szyf M (2013) DNA methylation, behavior and early life adversity. J Genet Genomics 40: 331–338. 23876773 10.1016/j.jgg.2013.06.004

[bibr560-0269881120959637] Tagliazucchi E Carhart-Harris R Leech R, et al. (2014) Enhanced repertoire of brain dynamical states during the psychedelic experience. Hum Brain Mapp 35: 5442–5456. 24989126 10.1002/hbm.22562 PMC6869695

[bibr561-0269881120959637] Takao K Nagatani T Kitamura Y, et al. (1995) Chronic forced swim stress of rats increases frontal cortical 5-HT2 receptors and the wet-dog shakes they mediate, but not frontal cortical β-adrenoceptors. Eur J Pharmacol 294: 721–726. 8750738 10.1016/0014-2999(95)00620-6

[bibr562-0269881120959637] Tedeschi RG (1999) Violence transformed: Posttraumatic growth in survivors and their societies. Aggress Violent Beh 4: 319–341.

[bibr563-0269881120959637] Teran FA Richerson GB (2020) 5-HT neurons and central CO2 chemoreception. In: Müller CP and Cunningham KA (eds) Handbook of Behavioral Neuroscience, vol. 31. Elsevier, pp. 377–391.

[bibr564-0269881120959637] Thoenes S Oberfeld D (2017) Meta-analysis of time perception and temporal processing in schizophrenia: Differential effects on precision and accuracy. Clin Psychol Rev 54: 44–64. 28391027 10.1016/j.cpr.2017.03.007

[bibr565-0269881120959637] Timmermann C Hannes K Letheby C, et al. (2019) September Psychedelics and Metaphysical Beliefs. Poster presented at Insight 2019, Berlin.

[bibr566-0269881120959637] Timmermann C Roseman L Williams L, et al. (2018) DMT models the near-death experience. Front Psychol 9: 1424. 10.3389/fpsyg.2018.01424 PMC6107838 30174629

[bibr567-0269881120959637] Tull MT Berghoff CR Wheeless LE, et al. (2018) PTSD symptom severity and emotion regulation strategy use during trauma cue exposure among patients with substance use disorders: Associations with negative affect, craving, and cortisol reactivity. Behav Ther 49: 57–70. 29405922 10.1016/j.beth.2017.05.005 PMC5805399

[bibr568-0269881120959637] Turecki G Brière R Dewar K, et al. (1999) Prediction of level of serotonin 2A receptor binding by serotonin receptor 2A genetic variation in postmortem brain samples from subjects who did or did not commit suicide. Am J Psychiatry 156: 1456–1458. 10484964 10.1176/ajp.156.9.1456

[bibr569-0269881120959637] Umbricht D Krljes S (2005) Mismatch negativity in schizophrenia: A meta-analysis. Schizophr Res 76: 1–23. 15927795 10.1016/j.schres.2004.12.002

[bibr570-0269881120959637] Vaidya VA Marek GJ Aghajanian GK, et al. (1997) 5-HT2A receptor-mediated regulation of brain-derived neurotrophic factor mRNA in the hippocampus and the neocortex. J Neurosci 17: 2785–2795. 9092600 10.1523/JNEUROSCI.17-08-02785.1997 PMC6573109

[bibr571-0269881120959637] van der Steen Y Gimpel-Drees J Lataster T, et al. (2017) Clinical high risk for psychosis: The association between momentary stress, affective and psychotic symptoms. Acta Psychiat Scand 136: 63–73. 28260264 10.1111/acps.12714

[bibr572-0269881120959637] Van Lommel P Van Wees R Meyers V, et al. (2001) Near-death experience in survivors of cardiac arrest: A prospective study in the Netherlands. Lancet 358: 2039–2045. 11755611 10.1016/S0140-6736(01)07100-8

[bibr573-0269881120959637] Van Os J Jones PB (2001) Neuroticism as a risk factor for schizophrenia. Psychol Med 31: 1129–1134. 11513380 10.1017/s0033291701004044

[bibr574-0269881120959637] van Wel JH Kuypers KP Theunissen EL, et al. (2012) Effects of acute MDMA intoxication on mood and impulsivity: Role of the 5-HT2 and 5-HT1 receptors. PLoS One 7: e40187. 22808116 10.1371/journal.pone.0040187 PMC3393729

[bibr575-0269881120959637] Varese F Barkus E Bentall RP (2012a) Dissociation mediates the relationship between childhood trauma and hallucination-proneness. Psychol Med 42: 1025–1036. 21896238 10.1017/S0033291711001826

[bibr576-0269881120959637] Varese F Smeets F Drukker M, et al. (2012b) Childhood adversities increase the risk of psychosis: A meta-analysis of patient-control, prospective-and cross-sectional cohort studies. Schizophrenia Bull 38: 661–671. 10.1093/schbul/sbs050 PMC3406538 22461484

[bibr577-0269881120959637] Vazquez DM Lopez JF Van Hoers H Watson SJ Levine S (2000) Maternal deprivation regulates serotonin 1A and 2A receptors in the infant rat. Brain Res 855: 76–82. 10650132 10.1016/s0006-8993(99)02307-0

[bibr578-0269881120959637] Veling W Susser E Van Os J, et al. (2008) Ethnic density of neighborhoods and incidence of psychotic disorders among immigrants. Am J Psychiatry 165: 66–73. 18086750 10.1176/appi.ajp.2007.07030423

[bibr579-0269881120959637] Visser AK Meerlo P Ettrup A, et al. (2014) Acute social defeat does not alter cerebral 5-HT2A receptor binding in male Wistar rats. Synapse 68: 379–386. 24823545 10.1002/syn.21750

[bibr580-0269881120959637] Vivot RM Pallavicini C Zamberlan F, et al. (2020) Meditation increases the entropy of brain oscillatory activity. Neurosci 431: 40–51. 10.1016/j.neuroscience.2020.01.033 32032666

[bibr581-0269881120959637] Vollenweider FX Leenders KL Scharfetter C, et al. (1997) Positron emission tomography and fluorodeoxyglucose studies of metabolic hyperfrontality and psychopathology in the psilocybin model of psychosis. Neuropsychoph 16: 357–372. 10.1016/S0893-133X(96)00246-1 9109107

[bibr582-0269881120959637] Vollenweider FX Vollenweider-Scherpenhuyzen MF Bäbler A, et al. (1998) Psilocybin induces schizophrenia-like psychosis in humans via a serotonin-2 agonist action. Neuroreport 9: 3897–3902. 9875725 10.1097/00001756-199812010-00024

[bibr583-0269881120959637] Vollmer LL Strawn JR Sah R (2015) Acid–base dysregulation and chemosensory mechanisms in panic disorder: A translational update. Transl Psychiat 5: e572. 10.1038/tp.2015.67 PMC4471296 26080089

[bibr584-0269881120959637] Wallace AF (1956) Revitalization movements. Am Anthropol 58: 264–281.

[bibr585-0269881120959637] Walsh R (1982) Psychedelics and psychological well-being. J Humanist Psychol 22: 22–32.

[bibr586-0269881120959637] Walsh Z Thiessen MS (2018) Psychedelics and the new behaviourism: Considering the integration of third-wave behaviour therapies with psychedelic-assisted therapy. Int Rev Psychiatr 30: 343–349. 10.1080/09540261.2018.1474088 30251904

[bibr587-0269881120959637] Waters F Chiu V Atkinson A, et al. (2018) Severe sleep deprivation causes hallucinations and a gradual progression toward psychosis with increasing time awake. Front Psychiat 9: 303. 10.3389/fpsyt.2018.00303 PMC6048360 30042701

[bibr588-0269881120959637] Watkins CC Andrews SR (2016) Clinical studies of neuroinflammatory mechanisms in schizophrenia. Schizophr Res 176: 14–22. 26235751 10.1016/j.schres.2015.07.018

[bibr589-0269881120959637] Watts R Day C Krzanowski J, et al. (2017) Patients’ accounts of increased ‘connectedness’ and ‘acceptance’ after psilocybin for treatment-resistant depression. J Humanist Psychol 57: 520–564.

[bibr590-0269881120959637] Watts R Luoma J (2019) The use of the psychological flexibility model to support psychedelic assisted therapy. J Contextual Behav Sci 15: 92–102.

[bibr591-0269881120959637] Weimer K Gulewitsch MD Schlarb AA, et al. (2013) Placebo effects in children: A review. Pediatr Res 74: 96–102. 23598811 10.1038/pr.2013.66

[bibr592-0269881120959637] Welsh SE Romano AG Harvey JA (1998) Effects of serotonin 5-HT2A/2C antagonists on associative learning in the rabbit. Psychopharmacology 137: 157–163. 9630002 10.1007/s002130050605

[bibr593-0269881120959637] Wessinger C (ed.) (2000) Millennialism, Persecution, and Violence: Historical Cases. Syracuse, NY: Syracuse University Press.

[bibr594-0269881120959637] Whitaker R (2011) Anatomy of an Epidemic: Magic Bullets, Psychiatric Drugs, and the Astonishing Rise of Mental Illness in America. New York: Random House Digital. 10.3109/01612840.2012.713447 23017048

[bibr595-0269881120959637] White WL (2004) Transformational change: A historical review. J Clin Psychol 60: 461–470. 15048693 10.1002/jclp.20001

[bibr596-0269881120959637] Wilkinson ST Wright D Fasula MK, et al. (2017) Cognitive behavior therapy may sustain antidepressant effects of intravenous ketamine in treatment-resistant depression. Psychother Psychosom 86: 162–167. 28490030 10.1159/000457960 PMC5516265

[bibr597-0269881120959637] Willard AK Norenzayan A (2017) ‘Spiritual but not religious’: Cognition, schizotypy, and conversion in alternative beliefs. Cognition 165: 137–146. 28544975 10.1016/j.cognition.2017.05.018

[bibr598-0269881120959637] Wilson B Cohen A (2015) The Big Book of Alcoholics Anonymous (Including 12 Steps, Guides & Prayers). New York: Alcoholics Anonymous World Services.

[bibr599-0269881120959637] Wimbush VL Valantasis R (eds) (2002) Asceticism. Oxford: Oxford University Press.

[bibr600-0269881120959637] Winkelman MJ White D (1987) A cross-cultural study of magico-religious practitioners and trance states: Database (HRAF Research Series in Quantitative Cross-Cultural Data III). Human Relations Area Files. doi:10.13140/RG.2.1.4381.2720.

[bibr601-0269881120959637] Winnicott D (1935) Manic defense. In: Winnicott D (ed.) Collected Papers: Through Pediatric to Psychoanalysis. New York: Basic Books, pp. 129–144.

[bibr602-0269881120959637] Woodsworth W (1884) Ode Intimations of Immortality from Recollections of Early Childhood. Boston: D. Lothrop and Company.

[bibr603-0269881120959637] Wong C Davidson L Anglin D, et al. (2009) Stigma in families of individuals in early stages of psychotic illness: Family stigma and early psychosis. Early Interv Psychia 3: 108–115. 10.1111/j.1751-7893.2009.00116.x PMC2748954 19777087

[bibr604-0269881120959637] World Health Organization (2017) Depression and Other Common Mental Disorders: Global Health Estimates (No. WHO/MSD/MER/2017.2). Geneva: World Health Organization.

[bibr605-0269881120959637] Wright IK Ismail H Upton N, et al. (1991) Effect of isolation rearing on 5-HT agonist-induced responses in the rat. Psychopharmacology 105: 259–263. 1839066 10.1007/BF02244319

[bibr606-0269881120959637] Xiang M Jiang Y Hu Z, et al. (2019) Serotonin receptors 2A and 1A modulate anxiety-like behavior in post-traumatic stress disordered mice. Am J Transl Res 11: 2288–2303. 31105836 PMC6511758

[bibr607-0269881120959637] Xu C Ma XM Chen HB, et al. (2016) Orbitofrontal cortex 5-HT2A receptor mediates chronic stress-induced depressive-like behaviors and alterations of spine density and Kalirin7. Neuropharmacology 109: 7–17. 26921771 10.1016/j.neuropharm.2016.02.020

[bibr608-0269881120959637] Xu ZH Yang Q Ma L, et al. (2012) Deficits in LTP induction by 5-HT2A receptor antagonist in a mouse model for fragile X syndrome. PLoS One 7: e48741. 23119095 10.1371/journal.pone.0048741 PMC3485341

[bibr609-0269881120959637] Yamada S Watanabe A Nankai M, et al. (1995) Acute immobilization stress reduces (±DOI)-induced 5-HT 2A receptor-mediated head shakes in rats. Psychopharmacology 119: 9–14. 7675955 10.1007/BF02246047

[bibr610-0269881120959637] Yanakieva S Polychroni N Family N, et al. (2019) The effects of microdose LSD on time perception: A randomised, double-blind, placebo-controlled trial. Psychopharmacology 236: 1159–1170. 30478716 10.1007/s00213-018-5119-x PMC6591199

[bibr611-0269881120959637] Yee JY Lee TS Lee J (2018) Levels of serum brain-derived neurotropic factor in individuals at ultra-high risk for psychosis—findings from the Longitudinal Youth at Risk Study (LYRIKS). Int J Neuropsychoph 21: 734–739. 10.1093/ijnp/pyy036 PMC6070044 29584866

[bibr612-0269881120959637] Yoshioka M Matsumoto M Togashi H (1995) Effects of conditioned fear stress on 5-HT release in the rat prefrontal cortex. Pharmacol Biochem Behav 51: 515–519. 7667378 10.1016/0091-3057(95)00045-x

[bibr613-0269881120959637] Young MB Norrholm SD Khoury LM, et al. (2017) Inhibition of serotonin transporters disrupts the enhancement of fear memory extinction by 3, 4-methylenedioxymethamphetamine (MDMA). Psychopharmacology 234: 2883–2895. 28741031 10.1007/s00213-017-4684-8 PMC5693755

[bibr614-0269881120959637] Yu B Becnel J Zerfaoui M, et al. (2008) Serotonin 5-hydroxytryptamine2A receptor activation suppresses tumor necrosis factor-α-induced inflammation with extraordinary potency. J Pharmacol Exp Ther 327: 316–323. 18708586 10.1124/jpet.108.143461

[bibr615-0269881120959637] Zamfir O Broqua P Baudrie V, et al. (1992) Effects of cold stress on some 5-HT1A, 5-HT1C and 5-HT2 receptor-mediated responses. Eur J Pharmacol 219: 261–269. 1385172 10.1016/0014-2999(92)90304-m

[bibr616-0269881120959637] Zammit S Lewis G Rasbash J, et al. (2010) Individuals, schools, and neighborhood: A multilevel longitudinal study of variation in incidence of psychotic disorders. Arch Gen Psychiat 67: 914–922. 20819985 10.1001/archgenpsychiatry.2010.101

[bibr617-0269881120959637] Zhang CQ Leeming E Smith P, et al. (2018) Acceptance and commitment therapy for health behavior change: A contextually-driven approach. Front Psychol 8: 2350. 10.3389/fpsyg.2017.02350 PMC5769281 29375451

[bibr618-0269881120959637] Zhang G Asgeirsdottir HN Cohen SJ, et al. (2013) Stimulation of serotonin 2A receptors facilitates consolidation and extinction of fear memory in C57BL/6J mice. Neuropharmacology 64: 403–413. 22722027 10.1016/j.neuropharm.2012.06.007 PMC3477617

[bibr619-0269881120959637] Zhang G Cinalli D Cohen SJ, et al. (2016a) Examination of the hippocampal contribution to serotonin 5-HT2A receptor-mediated facilitation of object memory in C57BL/6J mice. Neuropharmacology 109: 332–340. 27114257 10.1016/j.neuropharm.2016.04.033

[bibr620-0269881120959637] Zhang G Stackman RW Jr (2015) The role of serotonin 5-HT2A receptors in memory and cognition. Front Pharmacol 6: 225. 26500553 10.3389/fphar.2015.00225 PMC4594018

[bibr621-0269881120959637] Zhang Y Catts VS Sheedy D, et al. (2016b) Cortical grey matter volume reduction in people with schizophrenia is associated with neuro-inflammation. Transl Psychiat 6: e982. 10.1038/tp.2016.238 PMC5290336 27959331

[bibr622-0269881120959637] Zhang YQ Gao X Ji GC, et al. (2001) Expression of 5-HT2A receptor mRNA in rat spinal dorsal horn and some nuclei of brainstem after peripheral inflammation. Brain Res 900: 146–151. 11325358 10.1016/s0006-8993(01)02283-1

[bibr623-0269881120959637] Zhao X Meyers KT Marballi KK, et al. (2019) Environment rapidly upregulates serotonin 2A receptor expression via the immediate early gene Egr3. bioRxiv, 634410.

[bibr624-0269881120959637] Zuo W Wu L Mei Q, et al. (2019) Adaptation in 5-HT2 receptors-CaMKII signaling in lateral habenula underlies increased nociceptive-sensitivity in ethanol-withdrawn rats. Neuropharmacology 158: 107747. 31445991 10.1016/j.neuropharm.2019.107747

